# The bladder cancer m^6^A landscape is defined by global methylation dilution and focal 3′-UTR hypermethylation

**DOI:** 10.1038/s44319-026-00739-y

**Published:** 2026-03-23

**Authors:** Jonas Koch, Jinyun Xu, Felix Bormann, Vitor Coutinho Carneiro, Manuel Neuberger, Katja Nitschke, Malin Nientiedt, Philipp Erben, Maurice Stephan Michel, Manuel Rodriguez-Paredes, Frank Lyko

**Affiliations:** 1 Division of Epigenetics, DKFZ-ZMBH Alliance, German Cancer Research Center, 69120 Heidelberg, Germany; 2 Faculty of Biosciences, Heidelberg University, 69120 Heidelberg, Germany; 3 Bioinformatics.Expert UG, 12305 Berlin, Germany; 4 Department of Urology and Urosurgery, Medical Faculty Mannheim, University of Heidelberg, 68167 Mannheim, Germany; 5 DKFZ Hector Cancer Institute at the University Medical Center Mannheim, Mannheim, Germany

**Keywords:** Cancer, Epitranscriptomics, m^6^A, GLORI, VIRMA, Cancer, Chromatin, Transcription & Genomics, RNA Biology

## Abstract

N^6^-Methyladenosine (m^6^A) is the most abundant internal modification of eukaryotic mRNAs and regulates target transcripts throughout the mRNA life cycle. Although changes in m^6^A have been reported in human cancers, technical limitations have hindered a comprehensive understanding of the cancer-associated m^6^A landscape. Here, we use GLORI-sequencing to establish the first transcriptome-wide, single-nucleotide resolution maps of m^6^A in bladder cancer. Comparing bladder cancer and healthy bladder samples, we discover two key m^6^A signatures: a global dilution of methylation and a focal hypermethylation at 3′-UTRs. The global methylation dilution results from an increased expression of unmethylated transcripts and a decreased expression of methylated transcripts. In contrast, focal 3’-UTR hypermethylation is associated with the overexpression of VIRMA, a component of the m^6^A writer complex. A functional role of VIRMA is confirmed in knockdown experiments that reveal reduced 3’-UTR methylation and oncogenic phenotypes of bladder cancer cells. Our study is the first to describe the m^6^A epitranscriptomic landscape of cancer at single-base resolution and provides first insights into the processes that generate its characteristic signatures.

## Synopsis

Single-nucleotide quantitative m^6^A profiling reveals that bladder cancer is characterized by global methylation dilution and focal 3′-UTR hypermethylation driven by VIRMA. The findings uncover mechanisms shaping cancer-associated m^6^A patterns and link altered m^6^A deposition to oncogenic phenotypes.

- Establishment of the first base-resolution and quantitative m^6^A maps in human cancer.
- Identification of a global methylation dilution signature that is caused by expression changes of methylated and unmethylated transcripts.
- Identification of a focal 3′-UTR hypermethylation signature associated with VIRMA overexpression.

## Abstract (web summary)

Single-nucleotide quantitative m^6^A profiling reveals that bladder cancer is characterized by global methylation dilution and focal 3′-UTR hypermethylation driven by VIRMA. The findings uncover mechanisms shaping cancer-associated m^6^A patterns and link altered m^6^A deposition to oncogenic phenotypes.

## Graphical abstract

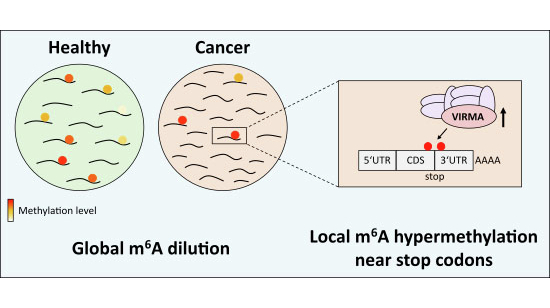

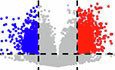

## Introduction

N^6^-Methyladenosine (m^6^A) is the most frequent internal modification of mammalian mRNAs and is present in 0.15–0.6% of all adenosine residues (He and He, 2021). The modification is deposited in the nucleus by the m^6^A writer complex, which consists of the METTL3, METTL14, WTAP, VIRMA, ZC3H13, CBLL1, RBM15, and RBM15B proteins (Zaccara et al, 2019). METTL3 represents the catalytically active writer enzyme, with the other components of the complex being responsible for RNA binding and additional regulatory functions (Zaccara et al, 2019). While the mechanisms underlying m^6^A patterning across the transcriptome remain poorly understood, results from HeLa cells have suggested that VIRMA acts as a regulatory subunit that recruits the m^6^A writer complex to 3′-UTRs and stop codon regions (Yue et al, 2018). m^6^A is predominantly found in the DRAC(H) consensus sequence, in which D = A, G or T; R = A or G; and H = A, C or U (Dominissini et al, 2012; Linder et al, 2015). The modification is read by the proteins of the YTH family, which comprises YTHDC1, YTHDF1, YTHDF2, and YTHDF3. These proteins were also shown to mediate m^6^A-dependent downstream functions (Flamand et al, 2023; Zaccara et al, 2019). In this context, different molecular mechanisms of m^6^A-dependent transcript regulation were described, including alternative splicing, alternative polyadenylation, transport, stability, and translation (Boulias and Greer, 2023; Flamand et al, 2023; Koch and Lyko, 2024). Importantly, deregulation of m^6^A has been found to affect physiological and pathophysiological processes, including cancer development (Lan et al, 2019).

Bladder cancer is a major global health problem and the 10th most frequent cancer worldwide (Saginala et al, 2020). As 90-95% of bladder cancers originate from urothelial cells, urothelial carcinoma of the bladder (UCB) represents the most common form of bladder cancer (Dyrskjøt et al, 2023; Siegel et al, 2024). About 75% of all patients are diagnosed with non-muscle-invasive bladder cancer (NMIBC). 10–20% of NMIBC cases further progress to muscle-invasive bladder cancer (MIBC), which is characterized by low survival rates and a high metastatic potential (Berdik, 2017; Lopez-Beltran et al, 2024; Sylvester et al, 2006). Approved early detection biomarkers for UCB are currently not available (Batista et al, 2020; Gill and Perks, 2024). Also, UCB incidence and prevalence numbers are expected to increase due to population growth and aging (Richters et al, 2020). Therefore, novel therapeutic drug targets and biomarkers are urgently needed for improving patient prognosis.

Multiple studies reported evidence for cancer-associated changes in the m^6^A epitranscriptome (Deng et al, 2022; Deng et al, 2023). This includes aberrant expression of m^6^A regulators and changes in the m^6^A methylation of individual transcripts related to UCB development (Yang et al, 2024b). Using LC-MS/MS analysis, we have shown recently that m^6^A levels were strongly reduced in UCB when compared to matched paratumoral tissue (Koch et al, 2023). This presents an apparent contradiction to other published findings where local hypermethylation of cancer-associated transcripts was described (Cheng et al, 2019). To resolve this inconsistency and to provide detailed maps of m^6^A in cancer, novel and robust m^6^A detection techniques are required. Previous studies relied on antibody-dependent mapping approaches with limited resolution and insufficient specificity (Helm et al, 2019; Koch and Lyko, 2024; McIntyre et al, 2020). Also, they were originally designed to be qualitative rather than quantitative. However, these methods were often used in a quantitative manner without appropriate spike-in standards or improved methodologies such as m^6^A-seq2 (Dierks et al, 2021). Similarly, initial attempts to map m^6^A distribution by direct RNA-sequencing were limited by low sequencing coverage and robustness (Li et al, 2024; Zhang et al, 2024). These methodological constraints have greatly limited our understanding of cancer-associated changes in m^6^A patterning and their functional implications in cancer development and progression.

GLORI-sequencing is a newly established method that allows for the site-specific, absolute quantification of m^6^A through an unbiased chemical deamination protocol (Liu et al, 2023). As similar methods, such as whole-genome bisulfite sequencing (Lister and Ecker, 2009) have been highly successful in mapping the cancer epigenome, we adopted GLORI to generate the first transcriptome-wide, base-resolution maps of m^6^A in cancer. We sequenced and compared a set of 9 clinical UCB samples and 9 independent paratumoral control samples and uncovered systematic differences in the m^6^A landscape of UCB. More specifically, the integration of RNA expression and m^6^A methylation data revealed a global dilution of methylation in UCB tissues. Simultaneously, UCB samples showed local 3’-UTR hypermethylation, which correlated with alternative mRNA polyadenylation. Interestingly, these changes were associated with the overexpression of VIRMA, a component of the m^6^A writer complex, which we found to be clinically relevant for the progression of UCB. Our findings thus uncover key signatures of the cancer m^6^A epitranscriptome and provide first insight into the underlying mechanisms.

## Results

### Establishment of an m^6^A methylation analysis pipeline

**Figure EV1:**
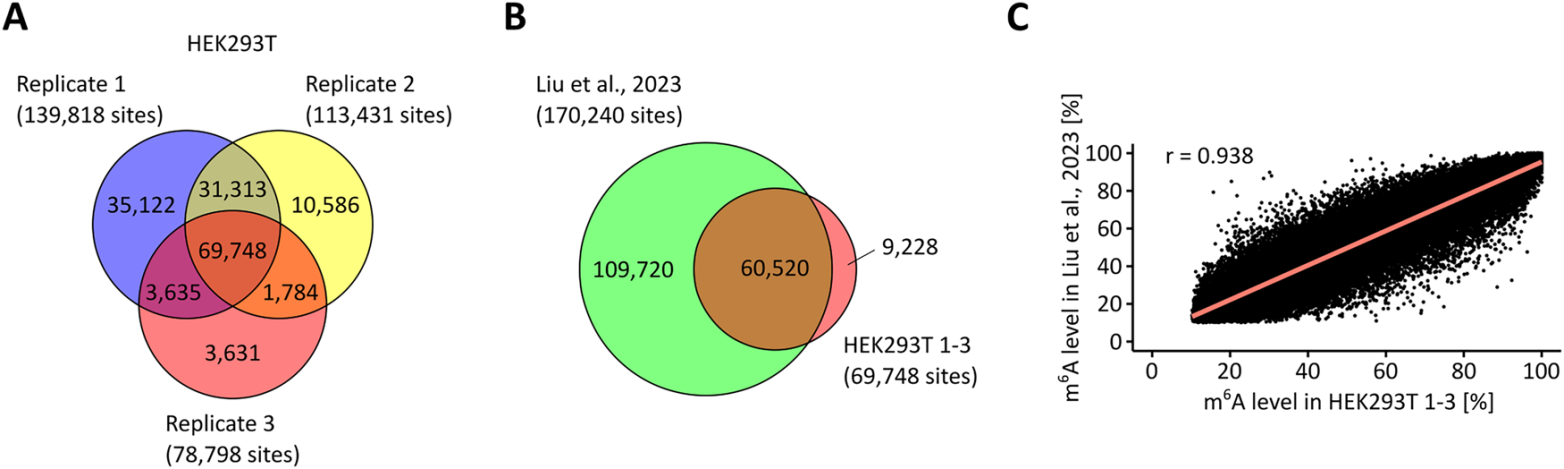
GLORI allows for the reproducible detection and quantification of m^6^A sites in HEK293T cells. (**A**) In the intersection of the three HEK293T cell replicates, roughly 70,000 m^6^A sites were detected by GLORI. (**B**) The majority (87%) of those 70,000 m^6^A sites were also reported by the original study. (**C**) The mean m^6^A methylation levels from the 60,000 m^6^A sites detected in both studies were comparable.

In an initial set of experiments, we attempted to reproduce the published GLORI results from HEK293T cells (Liu et al, 2023). Therefore, we sequenced libraries from three HEK293T cell replicates, with an average yield of 200 million sequencing reads, respectively, and mapping rates of 50% (Table EV1). As the number of detectable m^6^A sites depends on both the coverage and the methylation level, we applied stringent criteria, requiring a minimum coverage of 15 independent reads and a non-conversion rate of at least 10% to call a m^6^A site. With these parameters, we obtained median A-to-G conversion ratios of >99% (Table EV2), which is similar to the reported conversion ratios and strongly limits the influence of false-positive signals (Liu et al, 2023). In total, we detected a shared 69,748 m^6^A sites in the transcriptomes of the three HEK293T cell replicates (Fig. EV1A). The majority (87%) of these sites were also detected previously (Fig. EV1B), with differences being largely due to the higher sequencing depth of the original study (Liu et al, 2023). Also, methylation levels were found to be highly reproducible (Fig. EV1C). Finally, it should be noted that our results are in excellent agreement with results that were obtained by direct RNA-sequencing of the same batch of cells (Hewel et al, 2025), which provides important orthogonal validation for our GLORI-based mapping and quantification of m^6^A sites.

**Figure 1:**
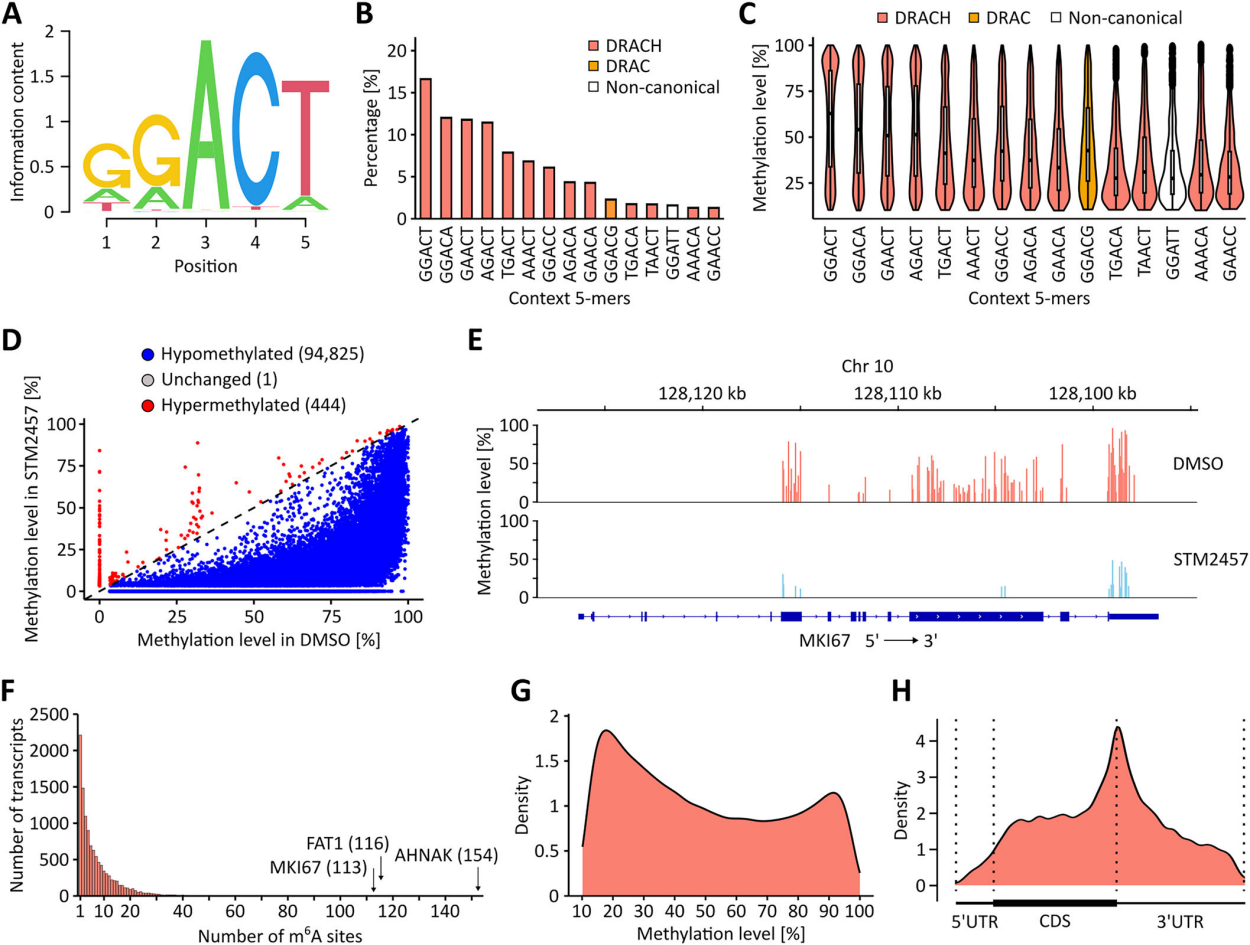
GLORI-based analysis of the m^6^A landscape in T24 UCB cells. (**A**) Sequence motif analysis of m^6^A sites revealing the DRAC(H) consensus motif. (**B**) Frequency of the 15 most detected m^6^A site motifs. DRAC(H) motifs were detected more frequently than non-canonical motifs. (**C**) Quantification of the m^6^A methylation level in the 15 most detected m^6^A site motifs. The lowest m^6^A methylation levels were detected in non-canonical motifs. *n* = 95,200 m^6^A sites shared among the three T24 replicates were used for sequence motif analyses. Boxplots were generated in R using ggplot2. The centre line indicates the median (50th percentile). The box bounds represent the first and third quartiles (25th and 75th percentiles), with box height equal to the interquartile range (IQR). Whiskers extend to the most extreme values within 1.5 * IQR from the quartiles. (**D**) Scatter plot comparing mean m^6^A levels of methylated DRAC(H) sites between STM2457-treated and DMSO-treated T24 cells. STM2457 treatment resulted in reduced methylation levels for 94,825 sites, while 444 sites were found to have increased methylation levels. (**E**) IGV browser tracks showing the detected m^6^A sites in the MKI67 transcript. STM2457 treatment led to a pronounced reduction of m^6^A methylation. (**F**) Quantification of m^6^A sites per transcript. While the median number was *n* = 4, some transcripts harbored very high numbers of m^6^A sites, as indicated. (**G**) Kernel density plot showing that the majority of m^6^A sites have either low or high methylation levels. (**H**) Metaplot showing that detected m^6^A sites predominantly occur in the CDS and 3’-UTR regions with a peak density surrounding the stop codon. These analyses were performed based on *n* = 3 biological replicates.

**Figure EV2:**
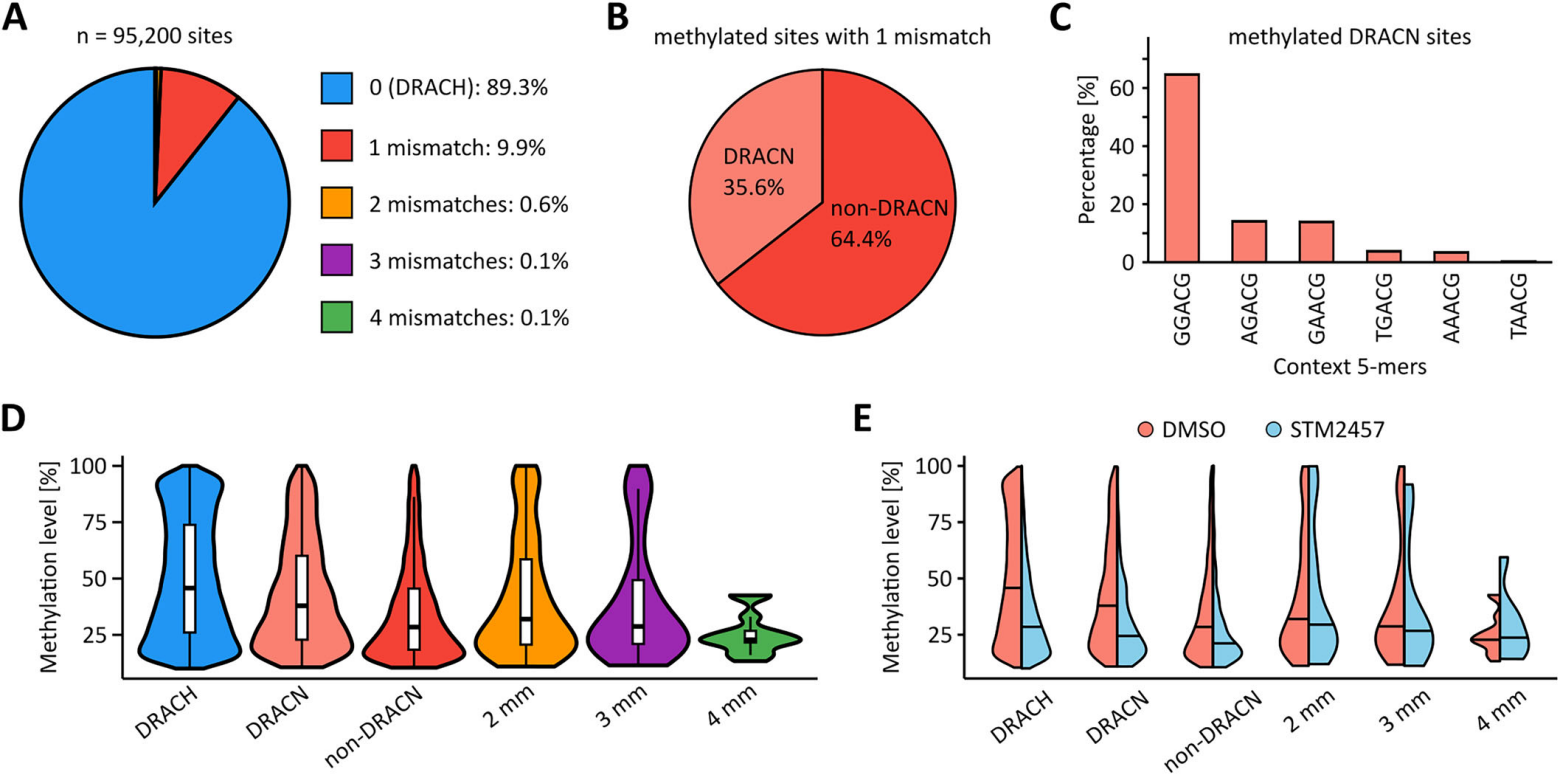
m^6^A sites detected in DRAC(H) motifs are the most robust. (**A**) m^6^A sites detected in different sequence motifs. Numbers describe mismatches compared to the canonical DRAC(H) motif. (**B**) Distribution of m^6^A sites detected in 5-mer motifs with one mismatch compared to DRAC(H). In DRACN motifs, the fifth base of the 5-mer is variable, while the first four bases are DRAC(H)-conform. In non-DRACN motifs, the mismatch occurs in one of the first four bases. (**C**) Frequency of DRACN motifs detected in T24 cells. (**D**) Methylation level distribution of m^6^A sites detected in the different motif categories. *n* = 95,200 m^6^A sites shared among the three T24 replicates were used for sequence motif analyses. Boxplots were generated in R using ggplot2. The centre line indicates the median (50th percentile). The box bounds represent the first and third quartiles (25th and 75th percentiles), with box height equal to the IQR. Whiskers extend to the most extreme values within 1.5 * IQR from the quartiles. (**E**) Methylation level distribution of m^6^A sites detected in the different motif categories in T24 cells treated with DMSO or the METTL3 inhibitor STM2457. mm = mismatch compared to DRAC(H). *n* = 95,200 m^6^A sites shared among the three T24 DMSO replicates and *n* = 17,189 m^6^A sites shared among the three T24 STM2457 replicates were used for this sequence motif analysis.

In the next step, we repeated the protocol for the T24 UCB cell line and again obtained high-quality GLORI datasets (Tables EV1 and EV2). Of note, the majority of reads mapped to mRNAs (>85%), while rRNA loci only accounted for <0.01% (Table EV3), consistent with a highly efficient enrichment of mRNAs during RNA preparations. Data analysis identified more than 95,000 m^6^A sites, which were predominantly found in the DRAC(H) consensus sequence motif (Fig. 1A). Within the 15 most frequent pentanucleotide motifs, canonical DRAC(H) motifs were strongly overrepresented (Fig. 1B). Also, higher and more evenly distributed m^6^A methylation levels were detected in the DRAC(H) motifs, while the non-canonical motifs were lowly methylated (Fig. 1C). Further in-depth motif analyses showed that roughly 90% of all m^6^A sites were detected in the DRAC(H) motif, while 10% of the sites were detected in motifs with one mismatch compared to DRAC(H) (Fig. EV2A). Sites detected in motifs with more than one mismatch were almost not detected (Fig. EV2A). When investigating the motifs with one mismatch, 36% were DRACN motifs, while 64% were non-DRACN motifs (mismatch occurring in the first four bases of motif, Fig. EV2B,C). Additionally, we investigated the distribution of methylation levels in the different motifs and found that the methylation levels in the motifs unrelated to DRAC(H) were substantially lower compared to the DRAC(H) motifs (Fig. EV2D). In further analyses we investigated the effect of the METTL3 inhibitor STM2457 in T24 cells. The results showed strongly reduced methylation, both transcriptome-wide (Fig. 1D) and at the level of specific transcripts (Fig. 1E). Effect sizes progressively decreased from DRAC(H) to DRACN and non-DRACN motifs (Fig. EV2E). Based on these observations, we considered the m^6^A sites in DRAC motifs as high-confidence methylation marks, while the m^6^A sites detected in non-canonical motifs were interpreted as deamination or sequencing artefacts. We therefore restricted all further analyses to m^6^A sites detected in DRAC consensus sequence motifs. Calculating the number of m^6^A sites per transcript showed a median of *n* = 4, but several transcripts were found to have multiple (up to 154) m^6^A sites (Fig. 1F). Overall, DRAC motifs showed a bimodal methylation distribution with peaks at 20% and 95% methylation (Fig. 1G). Metagene plots showed that the majority of m^6^A sites were in the CDS and 3’-UTR of the transcripts with a characteristic peak around the stop codon (Fig. 1H). These findings demonstrate the capacity of GLORI to faithfully map m^6^A sites.

### Comparative analysis of UCB and control samples

**Figure 2:**
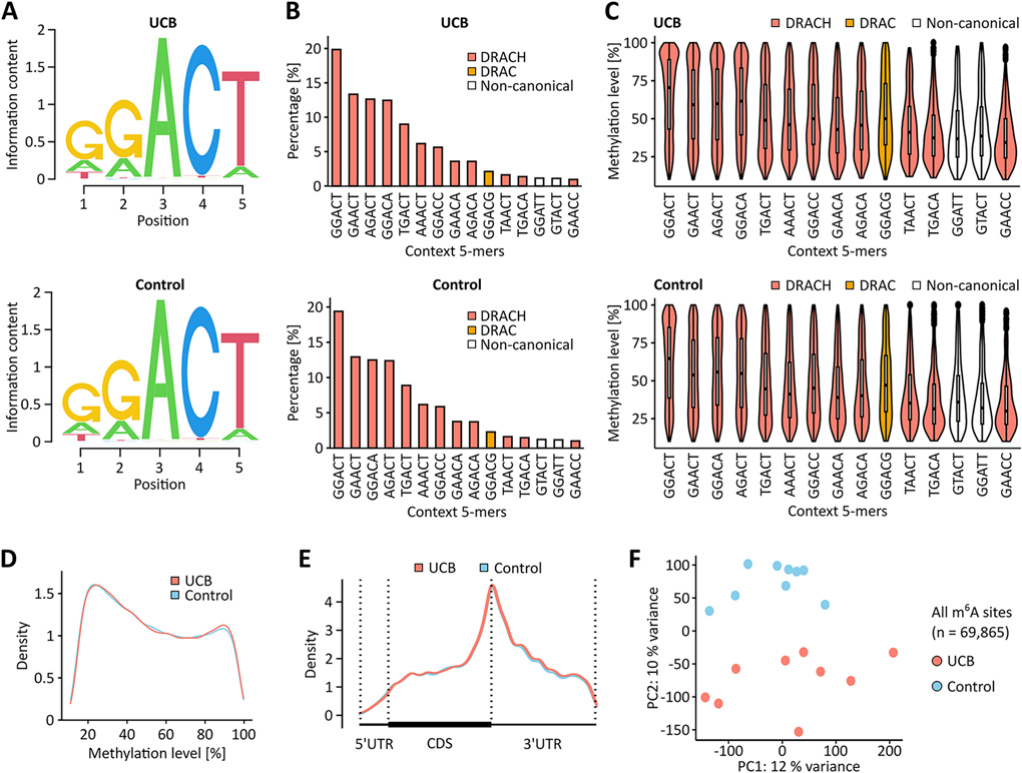
The m^6^A epitranscriptomic landscapes of UCB and non-malignant uroepithelial tissue show moderate, but systematic differences. (**A**) Sequence motif analyses using detected m^6^A sites in UCB and control tissue samples. In both tissue types, m^6^A sites were primarily detected in the DRAC(H) consensus sequence motif. (**B**) Frequencies of the 15 most detected motifs. (**C**) Quantification of the methylation level in the 15 most detected motifs. *n* = 42,339 m^6^A sites shared among the nine UCB tissue replicates were used for sequence motif analyses. *n* = 48,061 m^6^A sites shared among the nine control tissue replicates were used for sequence motif analyses. Boxplots were generated in R using ggplot2. The centre line indicates the median (50th percentile). The box bounds represent the first and third quartiles (25th and 75th percentiles), with box height equal to the IQR. Whiskers extend to the most extreme values within 1.5 * IQR from the quartiles. (**D**) Kernel density plot showing the distribution of m^6^A site methylation levels in the UCB and control datasets. (**E**) Metagene plots demonstrating that the detected m^6^A sites predominantly occur in CDS and 3’-UTR regions independent of the sample group. (**F**) Principal component analysis demonstrating that UCB and control tissue samples can be separated based on their m^6^A signatures. These analyses were performed based on *n* = 9 biological replicates.

**Figure EV3:**
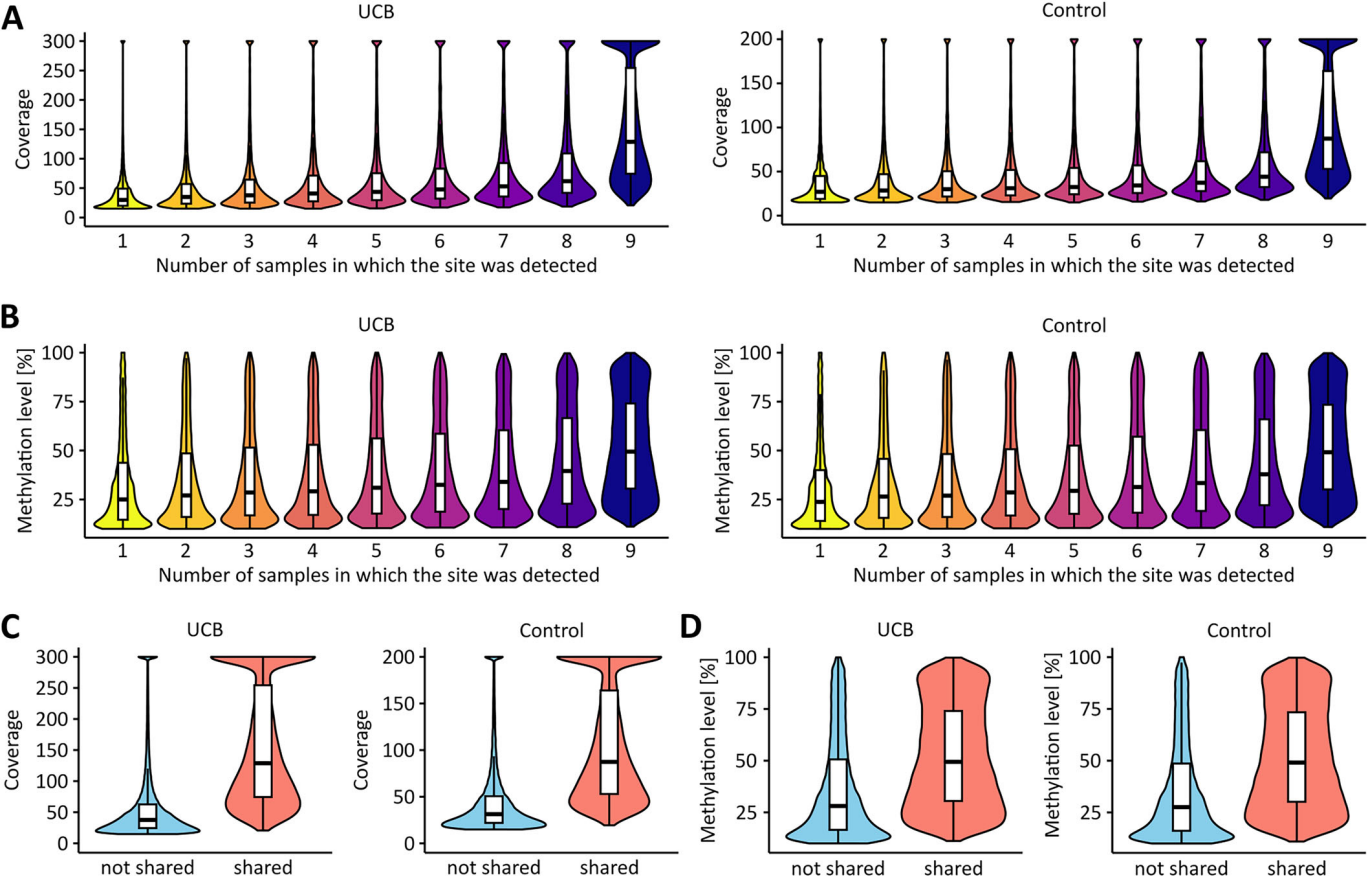
Shared m^6^A sites are characterized by high coverage and high methylation levels. (**A**) m^6^A sites were grouped based on the number of samples in which they were detected (from 1 to 9), and the distributions of sequencing coverage are shown for each detection category. (**B**) m^6^A sites were grouped based on the number of samples in which they were detected (from 1 to 9), and the distributions of methylation levels are shown for each detection category. (**C**) m^6^A sites detected in all nine samples (shared) were compared against those detected in only 1–8 samples (not shared), and the distributions of sequencing coverage are shown. (**D**) m^6^A sites detected in all nine samples (shared) were compared against those detected in only 1–8 samples (not shared) and the distributions of methylation levels are shown. *n* = 242,371 sites detected in nine UCB tissue samples and *n* = 191,722 sites detected in nine control tissue samples were used for these analyses. Boxplots were generated in R using ggplot2. The centre line indicates the median (50th percentile). The box bounds represent the first and third quartiles (25th and 75th percentiles), with box height equal to the IQR. Whiskers extend to the most extreme values within 1.5 * IQR from the quartiles.

**Figure EV4:**
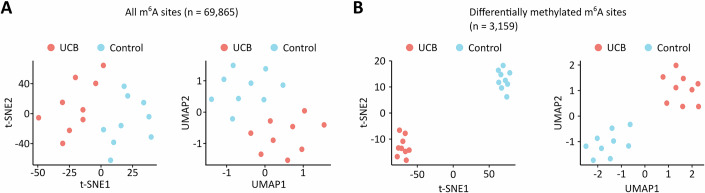
tSNE and UMAP analyses separating control and UCB tissue samples based on their m^6^A signatures. (**A**) Dimensionality reduction analyses considering all m^6^A sites. (**B**) Dimensionality reduction analyses considering differentially methylated m^6^A sites. These analyses were performed based on *n* = 9 biological replicates.

In the next step, we applied our GLORI pipeline to nine UCB and nine independent paratumoral control samples. Clinical information about the patient samples is provided in Table EV4. The quality of the corresponding GLORI datasets matched the high standards obtained with the HEK293T and T24 cell lines (Tables EV1 and EV2). Initial sequence motif analyses confirmed methylation in the consensus DRAC(H) motif for both sample groups (Fig. 2A) and did not reveal any detectable differences in pentanucleotide motif frequencies and methylation levels (Fig. 2B,C). These results strongly suggest that m^6^A motif specificity is retained in UCB. In total, approximately 42,000 detected m^6^A sites were shared among the UCB samples, while roughly 48,000 m^6^A sites were shared among the control samples and used for further downstream analysis. To assess whether differences in m^6^A site detection across samples could be explained by differences in coverage or conversion, we compared coverage and methylation levels between shared and non-shared sites. Indeed, shared sites that were detected in all 9 samples of a group showed higher median coverage (UCB: 129 vs. 37.7 reads; Control: 87.3 vs. 31.3 reads, Fig. EV3A,C) and higher median methylation levels (UCB: 0.495 vs. 0.278; Control: 0.491 vs. 0.273, Fig. EV3B,D) compared to sites detected in fewer samples. These findings indicate that strong signals (i.e., a combination of high coverage and high methylation) are more likely to be shared across samples. When comparing the distribution of methylation levels (Fig. 2D) and the localization of these m^6^A sites (Fig. 2E), we could not identify any major differences between UCB and control samples. Interestingly, however, principal component analysis based on all m^6^A sites showed that the tumor samples could be clearly separated from the controls (Figs. 2F and EV4A). These findings strongly suggest the presence of cancer-specific signatures in the m^6^A epitranscriptomic landscape.

### Differential methylation of cancer-related transcripts

**Figure 3:**
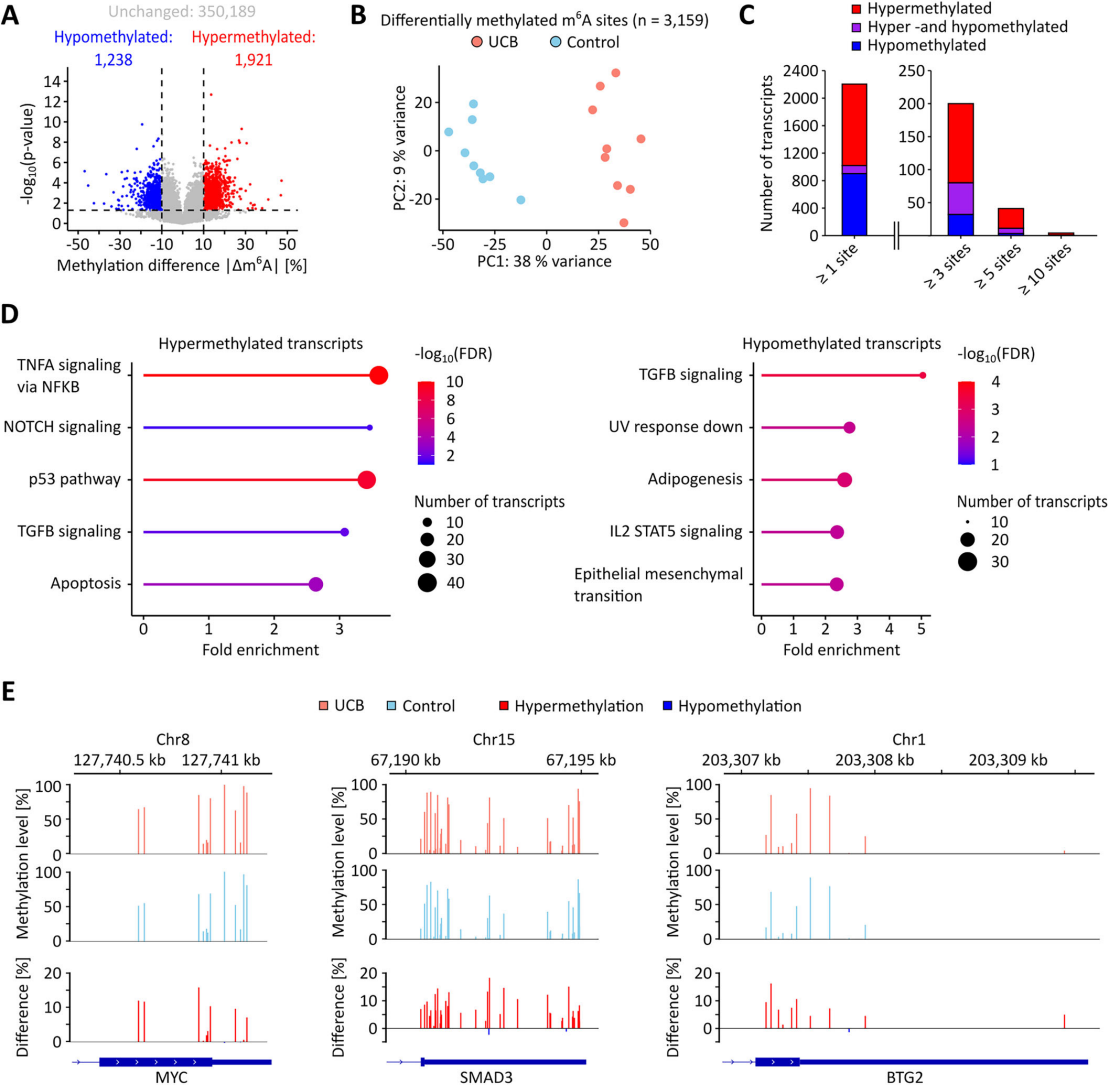
The UCB m^6^A epitranscriptome is characterized by both hypo- and hypermethylation. (**A**) Differential methylation analysis revealed that 1921 m^6^A sites were hypermethylated, while 1238 m^6^A sites were hypomethylated. Thresholds: Absolute difference in methylation level >10%, *p* < 0.05. *n* = 353,348 DRAC(H) sites were analyzed for differential methylation comparing nine UCB and nine control tissue samples. Statistical significance was assessed using beta binomial models. Unmethylated DRAC(H) sites are included in this analysis. (**B**) PCA showing that patient samples can be separated based on the differentially methylated m^6^A sites. (**C**) On the transcript level, 1186 transcripts were found to be hypermethylated, 117 transcripts had both hyper- and hypomethylated m^6^A sites, and 902 transcripts were hypomethylated, indicating that the majority of transcripts are coordinately hyper- or hypomethylated. (**D**) Top 5 enriched pathways based on differentially methylated transcripts. (**E**) Prominent examples for hypermethylated transcripts in UCB. These analyses were performed based on *n* = 9 biological replicates.

**Figure EV5:**
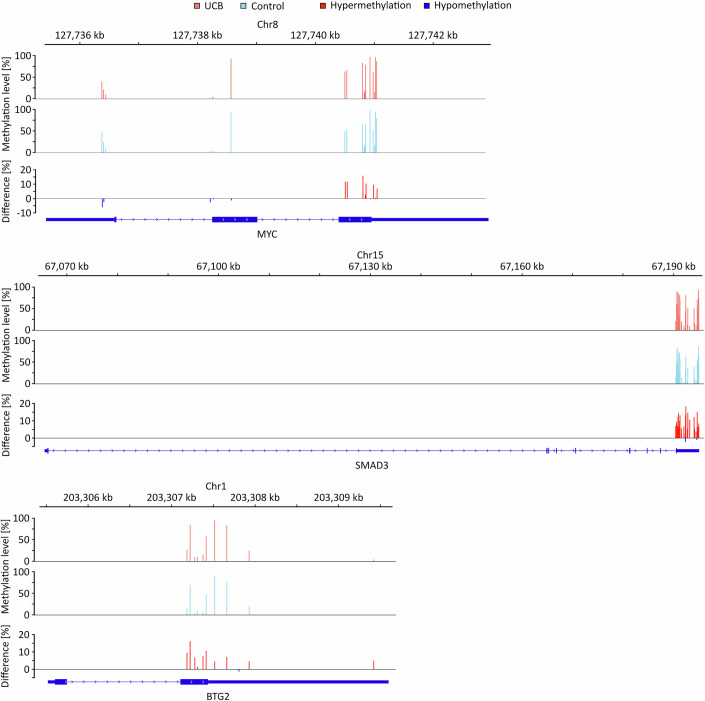
Methylation level changes in selected transcripts. IGV browser tracks of MYC, SMAD3, and BTG2 transcripts showing altered methylation levels in m^6^A sites comparing control and UCB tissues. These analyses were performed based on *n* = 9 biological replicates.

In the next step, we sought to further investigate the cancer-associated m^6^A pattern changes by identifying differentially methylated m^6^A sites using stringent criteria (|Δm^6^A| ≥ 10% and *p* < 0.05). The 10% cutoff was determined by analyzing the standard deviations (SD) of the methylation levels of each DRAC site in both groups. The vast majority of sites showed a SD < 10% in both tissue groups, with a mean of approx. 1% and the 90th percentile at <6% (Table EV5). A methylation level difference of 10% therefore robustly exceeds the observed (stochastic) variability. Transcripts were classified as hypermethylated or hypomethylated if at least one m^6^A site showed increased or decreased methylation, respectively. Transcripts with changes in both directions were ‘labeled hyper- and hypomethylated’. We identified 1921 sites with increased methylation levels in cancer (hypermethylated) and 1238 sites with reduced methylation levels in cancer (hypomethylated, Fig. 3A). Principal component and additional dimensionality reduction analyses based on these 3159 differentially methylated sites again showed a clear separation of the tumor and control sample groups (Figs. 3B and EV4B). On the transcript level, 1186 transcripts were identified to be hypermethylated, while 902 transcripts were hypomethylated (Fig. 3C). Only a small fraction of transcripts had both hyper-and hypomethylated m^6^A sites, indicating that most m^6^A sites change consistently across transcripts (Fig. 3C). Pathway analyses showed that the differentially methylated transcripts were enriched in several pathways that have been linked to UCB development (Goriki et al, 2018; Knowles and Hurst, 2015; Sui et al, 2017), including TNFα, NOTCH, p53, and TGFβ signaling, as well as pathways related to apoptosis and epithelial mesenchymal transition (EMT, Fig. 3D). Additional differentially methylated transcripts included MYC, SMAD3, and BTG2, which have all been linked to UCB (Cheng et al, 2019; Mao et al, 2015; Millet and Zhang, 2007; Yuniati et al, 2019) and were found hypermethylated in their CDS and 3’-UTR regions (Figs. 3E and EV5).

### Cancer-associated hypomethylation is due to cancer-associated changes in transcript abundance

**Figure 4:**
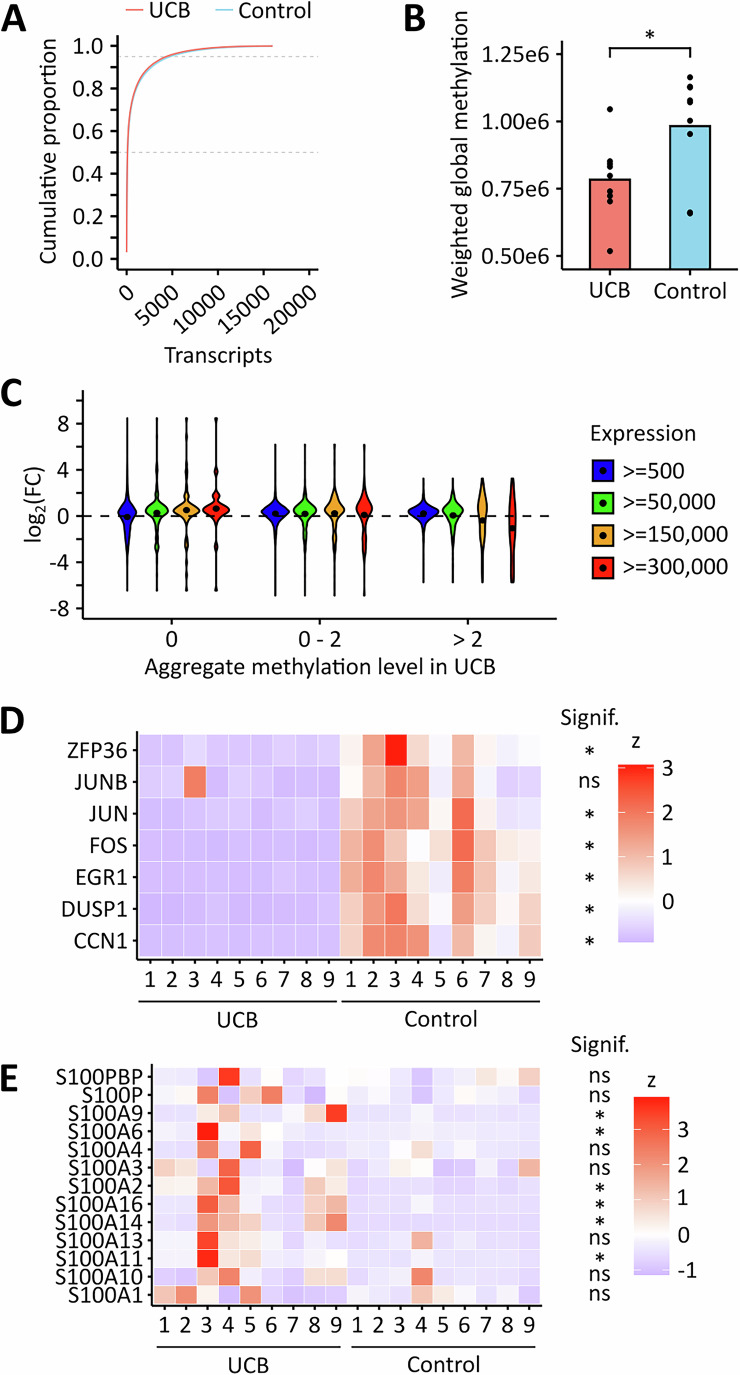
Global m^6^A hypomethylation in UCB results from changes in transcript abundance. (**A**) Cumulative proportion plot of control and UCB tissues. Few transcripts make up a high proportion of the transcriptome in both conditions. (**B**) Weighted global methylation levels of control and UCB tissues. The analysis confirms a global hypomethylation in UCB. *p = 0.026, t-test. (**C**) Global analysis of transcript expression changes in UCB considering the methylation status of the transcripts. When analyzing all transcripts, no major trends were observed. When restricting the analysis to highly abundant transcripts, an upregulation of unmethylated transcripts as well as a downregulation of highly methylated transcripts was observed in UCB. Expression and methylation data from *n* = 13,269 transcripts was used for this analysis. (**D**) Heatmap showing selected transcripts that had highest methylation levels in the lists of the most abundant transcripts in control and UCB tissues. Six out of the seven transcripts were downregulated in UCB. Threshold: q < 0.05. (**E**) Heatmap showing the expression of the S100 transcripts family. Several transcripts were found to be upregulated in UCB. Threshold: q < 0.05. These analyses were performed based on *n* = 9 biological replicates.

**Figure EV6:**
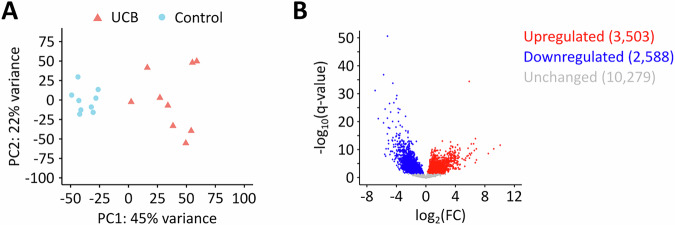
RNA sequencing analysis of clinical samples. (**A**) PCA demonstrating that patient samples can be separated based on their gene expression profile. (**B**) Differential gene expression analysis showing global deregulation of genes in UCB. q-value < 0.05. These analyses were performed based on *n* = 9 biological replicates.

As we observed both hypomethylation and hypermethylation in cancer samples, we performed further analyses to define the signatures of the cancer-associated m^6^A epitranscriptome. To address cancer-related differences in transcript abundance, we performed RNA-sequencing on the same tissue samples that we had used for GLORI. Principal component analysis showed that both sample groups could be separated based on their gene expression profiles (Fig. EV6A). Differential gene expression analysis demonstrated pronounced global deregulation of gene expression in UCB when compared to the control tissue, with 6,091 differentially expressed genes (Fig. EV6B). Cumulative analysis of normalized transcript levels showed that few transcripts made up a high proportion of the total transcriptome (Fig. 4A), with roughly 100 transcripts making up 50% of the transcriptome in both groups. This raised the possibility that methylation and/or expression changes in these highly abundant transcripts could strongly impact the global methylation level. Indeed, integrated analysis of GLORI- and RNA-sequencing data showed a significant global hypomethylation of UCB tissue samples (Fig. 4B), which confirmed the results observed in our previous LC-MS/MS analyses (Koch et al, 2023). Further data analysis revealed that unmethylated, highly abundant transcripts were upregulated, while highly methylated, highly abundant transcripts were downregulated in UCB (Fig. 4C). For example. several highly methylated transcripts (aggregate methylation >2), including EGR1, JUN, JUNB, and FOS, were markedly downregulated in UCB (Fig. 4D). In contrast, several unmethylated transcripts from the S100 family were upregulated in UCB (Fig. 4E) These findings strongly suggest that m^6^A marks become “diluted” in cancer samples, due to the increased presence of unmethylated transcripts.

### Cancer-associated hypermethylation is enriched at 3’-UTRs and associated with increased VIRMA expression

**Figure 5:**
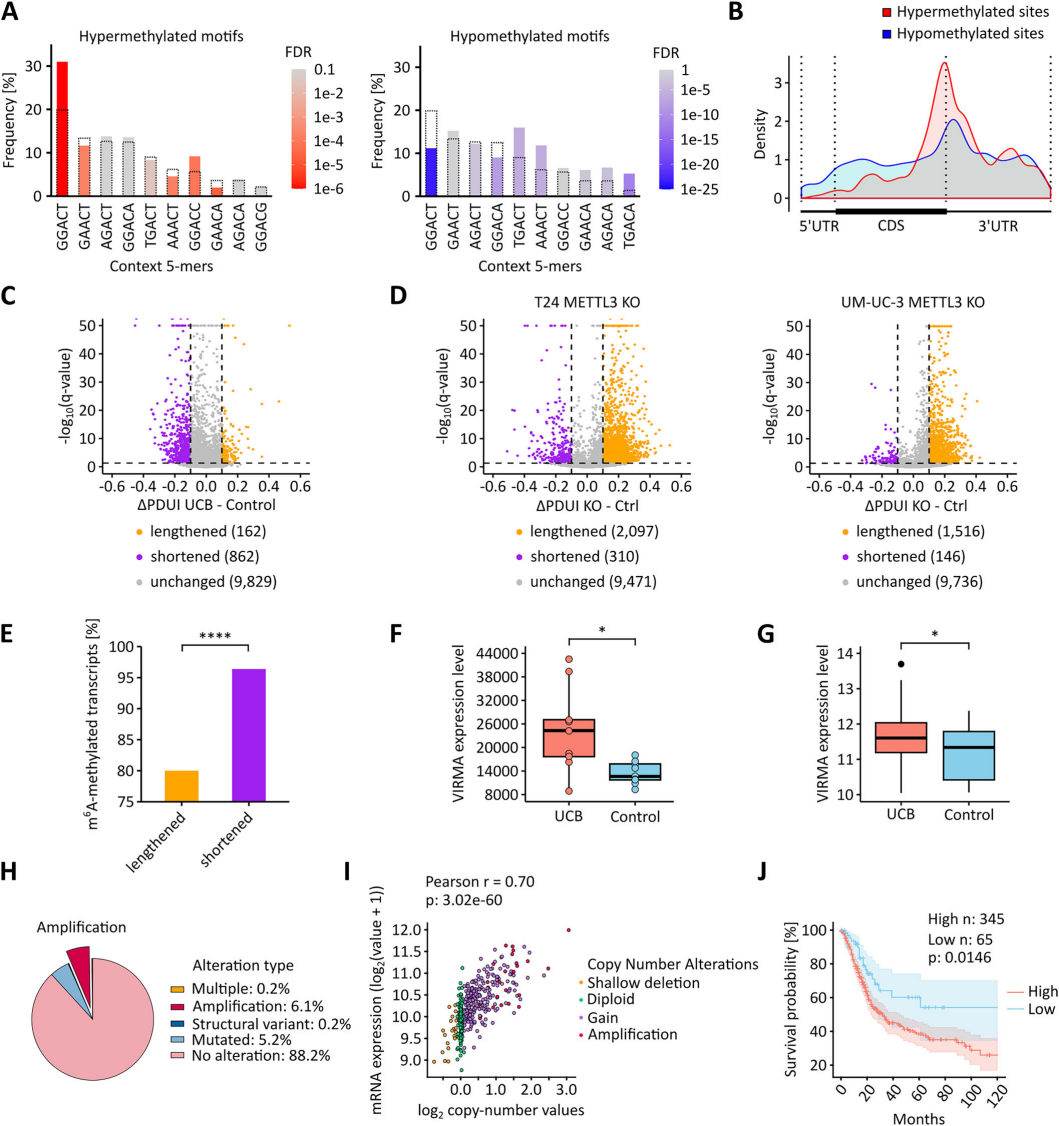
Local 3’-UTR hypermethylation is associated with an upregulation of VIRMA in UCB. (**A**) Frequency of the motifs detected in hypermethylated and hypomethylated m^6^A sites, respectively. Bars with dashed outlines represent the overall frequency of the respective motif in the cancer samples. Colored bars represent the frequency of the respective motif among the differentially methylated m^6^A sites. (**B**) Metagene plot showing that hypermethylated m^6^A sites predominantly occur in regions surrounding the stop codon. (**C**) Detection of alternative polyadenylation events using DaPars. Thresholds: Absolute difference in PDUI > 0.1, FDR < 0.05. The majority of transcripts have a negative ΔPDUI in UCB. *n* = 10,853 dynamic alternative polyadenylation usages were identified comparing nine UCB and nine control tissue samples. (**D**) Detection of alternative polyadenylation events in UCB METTL3 knockout clones. Global reduction of m^6^A leads to lengthening of transcripts. *n* = 11,878 and *n* = 11,398 dynamic alternative polyadenylation usages were identified comparing three METTL3 knockout (KO) and three control cell line samples. (**E**) Comparison of the proportion of m^6^A methylation in 3’-UTR lengthening or shortening. ****p = 6.301e-10, Fisher’s exact test. (**F**) Comparison of VIRMA mRNA expression between nine UCB and nine control tissue samples in our dataset. *p = 0.011, Mann–Whitney-U test. (**G**) Comparison of VIRMA mRNA expression between control (*n*  =  28) and UCB (*n*  =  407) tissue samples. Cancer samples are from the TCGA-BLCA cohort, control samples were combined from the TCGA-BLCA and GTEx cohorts. *p = 0.016, Mann–Whitney-U test. Boxplots were generated in R using ggplot2. The centre line indicates the median (50th percentile). The box bounds represent the first and third quartiles (25th and 75th percentiles), with box height equal to the IQR. Whiskers extend to the most extreme values within 1.5 * IQR from the quartiles. (**H**) Overview of genetic alterations of VIRMA in the TCGA-BLCA dataset. (**I**) Scatter plot depicting the correlation between the log_2_ copy-number values and VIRMA mRNA expression levels. (**J**) 10-year overall survival analysis of the TCGA-BLCA cohort. Patients were stratified into VIRMA-high (*n *= 345) and VIRMA-low (*n* = 65) groups. *p* = 0.0146, log-rank test.

**Figure EV7:**
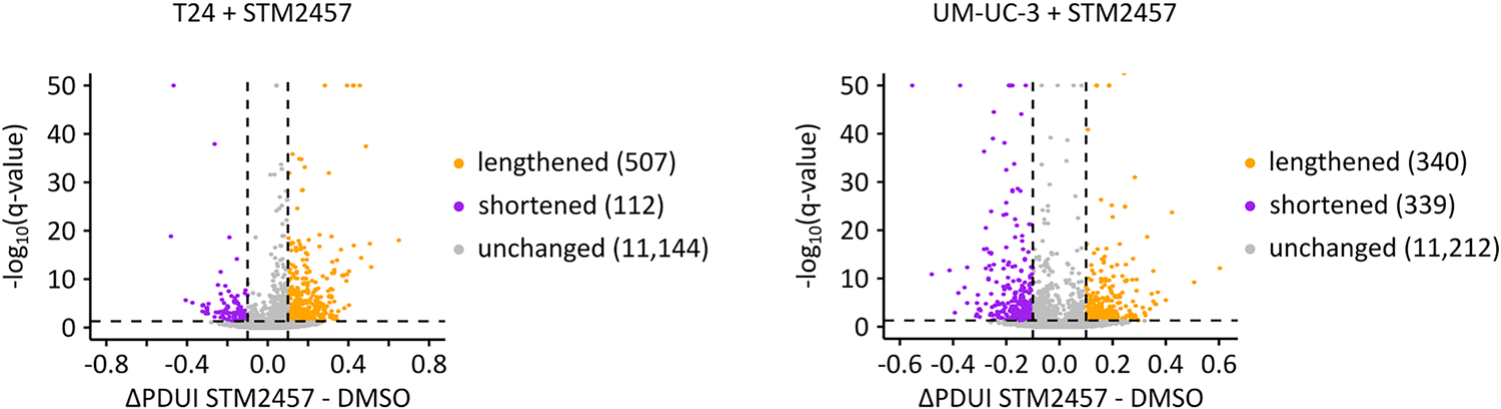
Detection of alternative polyadenylation events in two UCB cell lines treated with the METTL3 inhibitor STM2457. In T24 cells, most transcripts were found to be lengthened, while no clear tendency was observed in UM-UC-3 cells. These analyses were performed based on *n* = 3 biological replicates.

To further characterize the contrasting signature, i.e., cancer-associated hypermethylation, we performed sequence motif analyses. The results showed that hypermethylation was frequently detected in the GGACT motif, while hypomethylated m^6^A sites were often found in the TGACT and AAACT motifs (Fig. 5A). Furthermore, metagene analyses found that hypermethylated m^6^A sites were predominantly located in the 3’-UTR, especially in proximity to the stop codon, while no clear patterns were detectable for hypomethylated sites (Fig. 5B). As m^6^A methylation changes have been shown to affect mRNA polyadenylation (Yue et al, 2018), we analyzed our datasets for evidence of alternative polyadenylation. Indeed, the majority of transcripts had negative difference of Percentage of Distal polyA site Usage Indices (PDUIs) indicating that they were shortened (Fig. 5C). To test whether m^6^A could affect alternative polyadenylation in UCB, we expanded our alternative polyadenylation analyses to UCB METTL3 knockout cell clones and to UCB cell lines treated with the METTL3 inhibitor STM2457. RNA-seq data analysis showed that most transcripts had positive ΔPDUIs upon METTL3 knockout and inhibition (Figs. 5D and EV7). The comparison of the proportion of m^6^A-methylated transcripts that are 3’-UTR lengthened or shortened also showed that shortened transcripts are more likely to be m^6^A-methylated (Fig. 5E). Our results thus establish hypermethylation near stop codons as a second important signature of the cancer-associated m^6^A epitranscriptome and indicate that it may affect alternative polyadenylation.

Hypermethylation of m^6^A sites in 3’-UTR and stop codon regions has been associated with VIRMA in HeLa cells (Yue et al, 2018). We therefore determined the mRNA expression levels of VIRMA in our sample set. The results showed that VIRMA was significantly upregulated in UCB (Fig. 5F). Combined TCGA and GTEx cohort analyses further confirmed that VIRMA is significantly overexpressed in UCB when compared to healthy bladder tissue (Fig. 5G). To explore the underlying mechanism driving VIRMA overexpression, we examined genetic alterations in UCB patients. Notably, VIRMA gene amplification was observed in approximately 6% of tumors (Fig. 5H), which is consistent with the known amplification rates of other UCB-associated genes, including MYC (2.9–3.3% (Kluth et al, 2023; Zaharieva et al, 2005)), FGFR1 (3.7–7% (Bou Zerdan et al, 2023; Helsten et al, 2016)), and HER2 (8–8.7% (Bou Zerdan et al, 2023; Fleischmann et al, 2011)). The link between VIRMA amplification and overexpression was further supported by a correlation analysis of VIRMA copy number values and mRNA expression levels, which demonstrated a highly significant (*p* = 3.02e-60, Pearson correlation) positive association (Fig. 5I). Finally, survival analysis revealed that UCB patients with higher VIRMA expression had a significantly worse prognosis (Fig. 5J). These findings suggest that VIRMA can play an important role in UCB progression, through increased expression and hypermethylation of 3′-UTRs.

### VIRMA depletion reduces m^6^A methylation and oncogenic phenotypes of UCB cells

**Figure 6:**
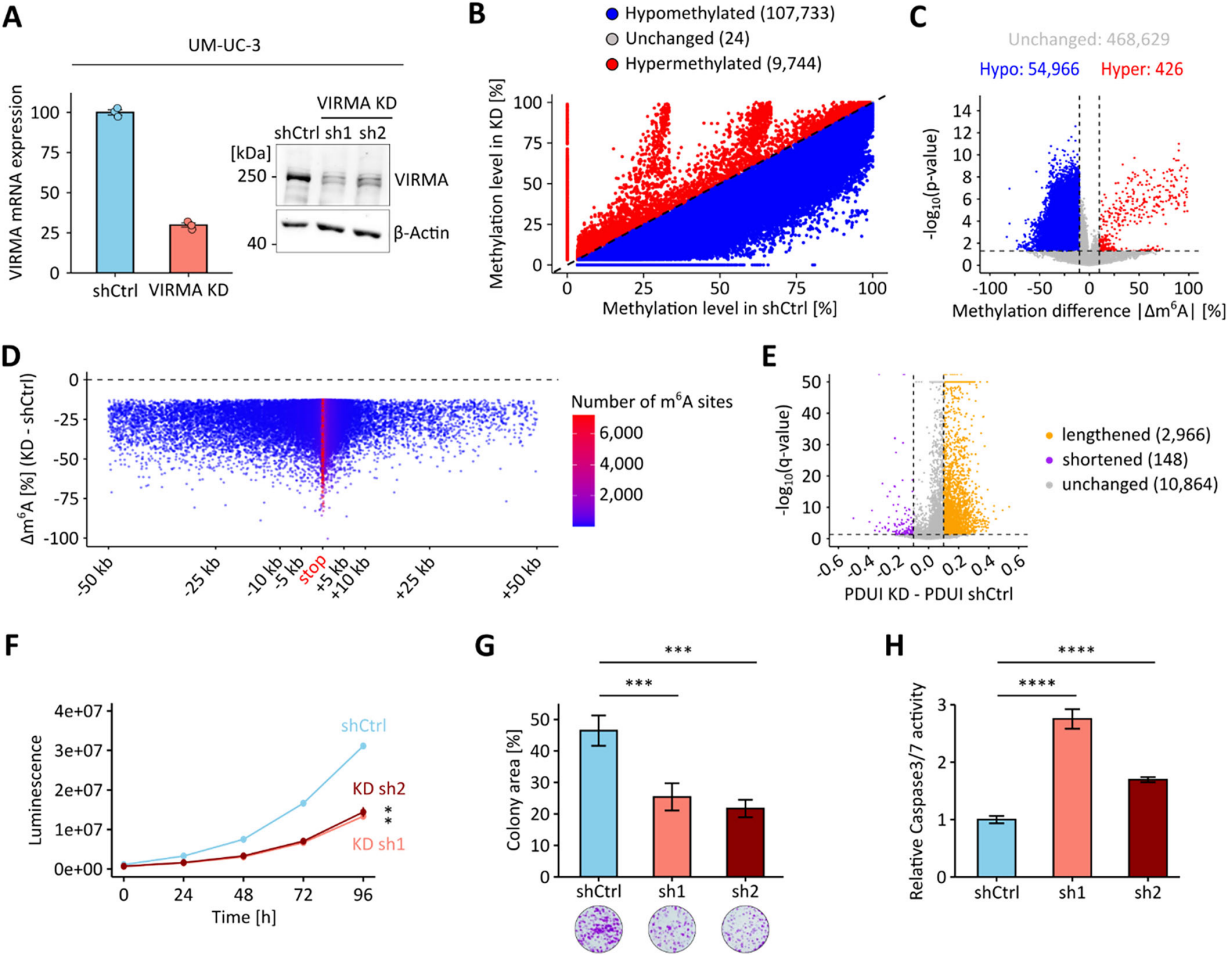
VIRMA depletion reduces m^6^A methylation and impairs the oncogenic phenotype of UM-UC-3 cells. (**A**) RNA-seq and Western blot analyses of VIRMA expression levels in UM-UC-3 VIRMA KD and shCtrl cells. (**B**) Scatter plot comparing mean m^6^A levels of methylated DRAC(H) sites between UM-UC-3 VIRMA KD and shCtrl cell lines. (**C**) Overview of differentially methylated DRAC(H) sites in VIRMA-depleted UM-UC-3 cells, based on |Δmethylation| > 10% and *p* < 0.05 thresholds. Statistical significance was assessed using beta binomial models. Unmethylated DRAC(H) sites are included in this analysis. (**D**) Δm^6^A levels (UM-UC-3 VIRMA KD - shCtrl) for differentially hypomethylated m^6^A sites were plotted across transcript regions surrounding the stop codon. (**E**) DaPars-based analysis of APA showing ΔPDUI for UM-UC-3 VIRMA-depleted cells compared to shCtrl cells. These analyses were performed based on *n* = 3 biological replicates. (**F**) Cell proliferation of UM-UC-3 VIRMA KD and shCtrl cells. sh1*p = 0.033, sh2*p = 0.045, two-way analysis of variance. (**G**) Colony formation results from UM-UC-3 VIRMA KD and shCtrl cells. sh1***p = 0.0007, sh2***p = 0.0006, two-tailed Student’s t test. (**H**) Caspase 3/7 activity measurements in UM-UC-3 VIRMA KD and shCtrl cells. ****p < 0.0001, two-tailed Student’s t test. Data are represented as mean ± SD; *n* = 4 biological replicates. Source data are available online for this figure.

**Figure EV8:**
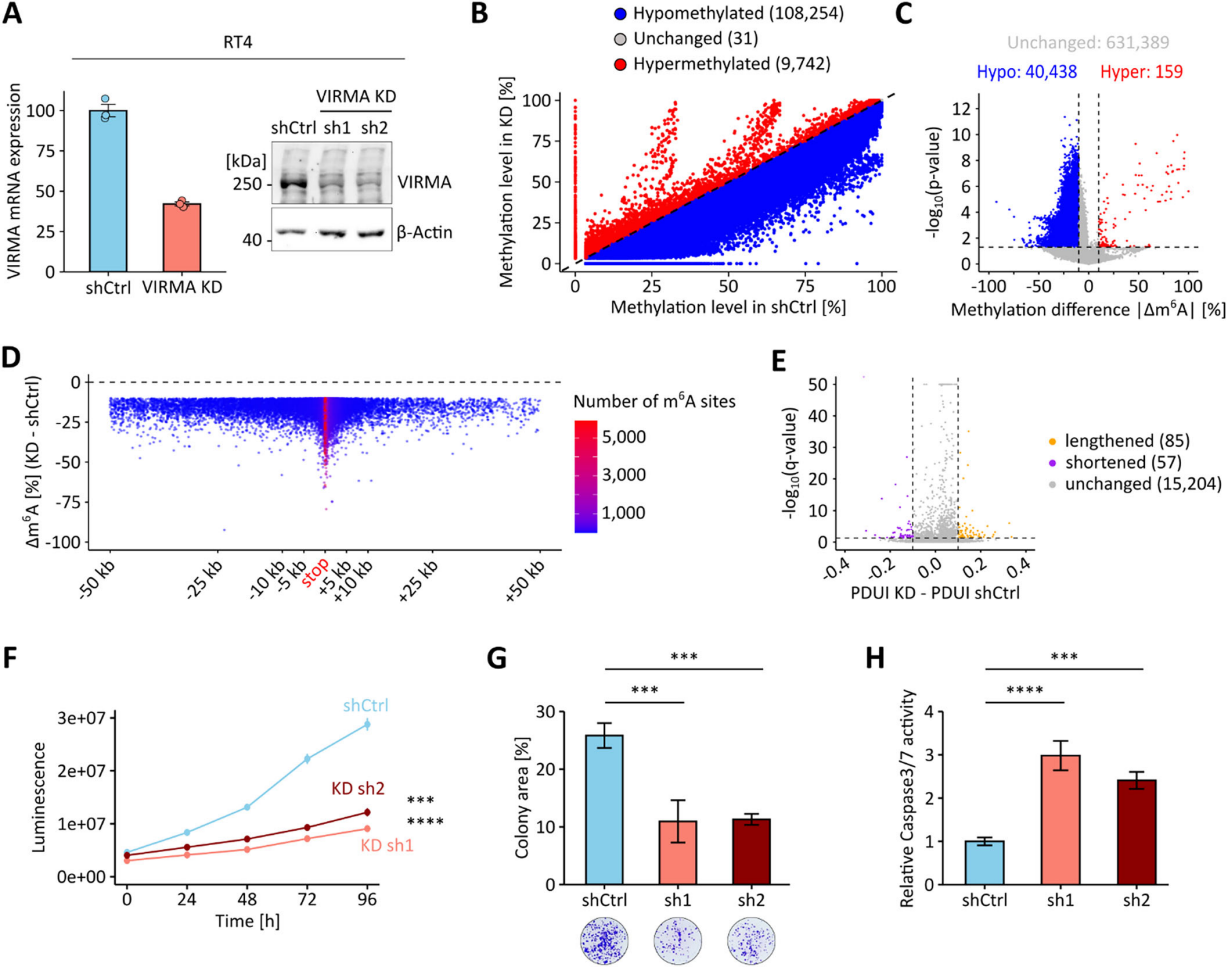
VIRMA depletion reduces m^6^A methylation and impairs the oncogenic phenotype of RT4 cells. (**A**) RNA-seq and Western blot analyses of VIRMA expression levels in RT4 VIRMA KD and shCtrl cells. (**B**) Scatter plot comparing mean m^6^A levels of methylated DRAC(H) sites between RT4 VIRMA KD and shCtrl cell lines. (**C**) Overview of differentially methylated DRAC(H) sites in VIRMA-depleted RT4 cells, based on |Δmethylation| > 10% and *p* < 0.05 thresholds. Statistical significance was assessed using beta binomial models. Unmethylated DRAC(H) sites are included in this analysis. (**D**) Δm^6^A levels (RT4 VIRMA KD - shCtrl) for differentially hypomethylated m^6^A sites were plotted across transcript regions surrounding the stop codon. (**E**) DaPars-based analysis of APA showing ΔPDUI for RT4 VIRMA-depleted cells compared to shCtrl cells. These analyses were performed based on *n* = 3 biological replicates. (**F**) Cell proliferation of RT4 VIRMA KD and shCtrl cells. sh1****p < 0.0001, sh2***p = 0.0008, two-way analysis of variance. (**G**) Colony formation results from RT4 VIRMA KD and shCtrl cells. ***p = 0.0007, two-tailed Student’s t test. (**H**) Caspase 3/7 activity measurements in RT4 VIRMA KD and shCtrl cells. sh1****p < 0.0001, sh2***p = 0.0001, two-tailed Student’s t test. Data are represented as mean ± SD; *n* = 4 biological replicates. Source data are available online for this figure.

**Figure EV9:**
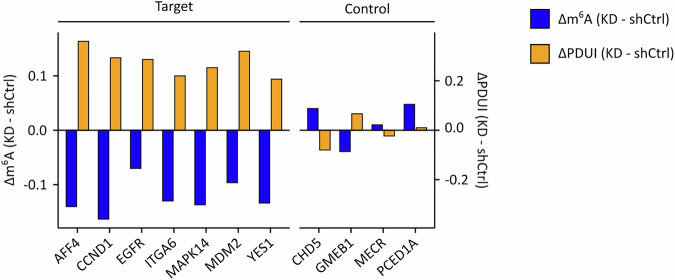
VIRMA KD-associated changes in m^6^A methylation and alternative polyadenylation at the transcript level. Bar plots show changes in m^6^A methylation (Δm^6^A; left y-axis) and polyadenylation site usage (ΔPDUI; right y-axis) upon VIRMA KD in UM-UC-3 cells. Δm^6^A was calculated as the mean m^6^A methylation level across all identified m^6^A sites within the terminal exon and 3′-UTR of each transcript. Target genes show reduced m^6^A methylation accompanied by increased ΔPDUI values, indicative of transcript lengthening. Δm^6^A and ΔPDUI values are shown relative to shCtrl conditions, *n* = 3 biological replicates.

To further analyze the functional relevance of VIRMA in UCB, we used shRNAs to knock down VIRMA expression. This resulted in a pronounced reduction of VIRMA mRNA and protein expression in UM-UC-3 cells (Fig. 6A) and are more moderate reduction in RT4 cells (Fig. EV8A). Subsequent GLORI-seq analysis revealed a marked global reduction in m^6^A levels in both cell lines when comparing the mean methylation levels of methylated DRAC(H) sites across all replicates (Fig. 6B and Fig. EV8B). To identify VIRMA-dependent m^6^A sites, we performed differential methylation analysis (|Δm^6^A| ≥10% and *p* < 0.05), which revealed widespread hypomethylation upon VIRMA KD (Figs. 6C and EV8C). Notably, when examining the positional distribution of these hypomethylation events, we observed that the strongest and densest methylation level reductions localized to regions surrounding the stop codon (Figs. 6D and EV8D), consistent with a role of VIRMA in 3’-UTR methylation. Global alternative polyadenylation profiling in the VIRMA KD models also showed an effect on transcript lengthening, which appeared pronounced in UM-UC-3 VIRMA KD cells (Fig. 6E) and more moderate in RT4 VIRMA KD cells (Fig. EV8E). The observed differences may be related to differences in KD efficiency, which was more pronounced in UM-UC-3 cells. At the individual transcript level, selected target transcripts showed both a loss of m^6^A methylation within terminal exons and 3′-UTRs and preferential distal polyadenylation site usage, while control transcripts did not display a consistent trend in either direction (Fig. EV9). Notably, m^6^A-dependent regulation of AFF4 and ITGA6 has previously been described in bladder cancer (Cheng et al, 2019; Jin et al, 2019), while the remaining target genes are known cancer-associated transcripts reported to be m^6^A-regulated in other tumor entities (Cai et al, 2021; Hirayama et al, 2020; Sang et al, 2022; Wang et al, 2023; Zhao et al, 2023). Control genes lack reported roles of m^6^A-dependent regulation in cancer. Finally, we also analyzed the effect of VIRMA KD on cancer cell phenotypes. The results showed that VIRMA KD strongly impaired cell proliferation and colony-forming capacity, accompanied by elevated Caspase-3/7 activity, indicating increased apoptotic signaling in both cell lines (Figs. 6F–H and EV8F–H). These findings are consistent with our observations in UCB patients and support a functional role for VIRMA in promoting tumor cell growth and survival.

## Discussion

We have used GLORI-sequencing of nine UCB tumor samples and nine independent paratumoral control samples to establish the first base-resolution maps of the m^6^A in UCB. Sequence motif analyses showed that GLORI detected m^6^A sites predominantly in DRAC(H) consensus sequence motifs, consistent with the known specificity of the m^6^A writer complex (Dominissini et al, 2012; Linder et al, 2015; Meyer et al, 2012). While cancer-related mutations in the m^6^A writer complex have been suggested to induce m^6^A methylation in non-canonical motifs (Zhang et al, 2023), our GLORI analysis of UCB samples showed that m^6^A sites were predominantly detected in the context of the DRAC(H) motif. These findings support earlier studies reporting relatively robust m^6^A profiles across different physiological cell and tissue types (Liu et al, 2020; Schwartz et al, 2014), and expand them to cancer. However, we also identified systematic alterations between the m^6^A profiles of UCB and control tissues, which allowed their clear and unambiguous separation in dimensionality reduction analyses. A prominent example was the MYC oncogenic transcript, which is known to be regulated in an m^6^A-dependent manner in UCB (Cheng et al, 2019), and which we found to be hypermethylated in its CDS and 3’-UTR regions. Furthermore, we found pronounced hypermethylation in transcripts from other cancer genes, such as SMAD3 and BTG2 (Mao et al, 2015; Millet and Zhang, 2007; Yuniati et al, 2019). These findings suggest the possibility to develop novel biomarkers from the m^6^A profile of UCB, which would address a major unmet clinical need for this tumor entity (Batista et al, 2020).

By integrating information about mRNA abundances from standard RNA-sequencing into our GLORI analyses, we determined methylation levels relative to transcript abundance. This novel computational approach allows for a more accurate and comprehensive assessment of the m^6^A epitranscriptomic landscape by avoiding bias from highly abundant or underrepresented transcripts. When comparing the correspondingly adjusted methylation levels of UCB and control tissues, we found a global reduction of m^6^A in UCB, which is consistent with our previous data obtained by LC-MS/MS (Koch et al, 2023). Further analyses suggested that this global reduction was primarily caused by the upregulation of abundant, unmethylated transcripts, which diluted the global methylation level, and by the downregulation of abundant, highly methylated transcripts. These observations are consistent with the hypothesis that changes in m^6^A levels across different subcellular compartments, treatments, or tissue samples result from changes in the mRNA metabolism that affect transcript abundance (Shachar et al, 2024). Furthermore, our results highlight the limits of standard m^6^A quantification and/or mapping approaches that do not consider transcript abundances.

Simultaneously, our analysis identified a high density of hypermethylated sites in UCB near stop codons, a feature that has been linked to VIRMA and alternative polyadenylation (Yue et al, 2018). The depletion of VIRMA resulted in a pronounced global loss of m^6^A, in line with LC-MS/MS measurements from four VIRMA-depleted breast cancer cell lines (Lee et al, 2023). The strongest reduction was shown to occur at 3’-UTR sites, consistent with the existing model in which VIRMA guides the writer complex toward these regions (Yue et al, 2018).

Alternative polyadenylation is the process by which different isoforms of the same transcript can be formed based on the usage of proximal or distant polyadenylation sites, which can affect transcript stability, localization, and translation (Tian and Manley, 2017), with implications for tumor formation (Yuan et al, 2021). When analyzing alternative polyadenylation events in our samples, we found pronounced 3’-UTR shortening of transcripts in UCB, which is consistent with previous findings in UCB and other cancer entities (Xia et al, 2014). Conversely, VIRMA depletion promoted 3’-UTR lengthening, which supports published data describing a link between m^6^A and 3’-UTR shortening (Molinie et al, 2016; Yue et al, 2018). Furthermore, RNA-dependent interactions between VIRMA and the polyadenylation factors CPSF5 and CPSF6 have been reported (Yue et al, 2018). Together, these findings raise the possibility that m^6^A deposition by the writer complex and VIRMA cooperate with the polyadenylation machinery to promote proximal polyadenylation site selection. Because widespread transcript shortening is a common feature of cancer transcriptomes, often enabling escape from post-transcriptional repression (Xia et al, 2014), the frequent upregulation of METTL3 and VIRMA in tumors (Destefanis et al, 2024; Koch and Lyko, 2024) may contribute to oncogenic 3′-UTR remodeling.

Our findings also show that VIRMA KD reduced cell proliferation as well as colony formation, and enhanced apoptosis signaling in two UCB cell lines, demonstrating that VIRMA promotes an oncogenic cellular phenotype. Similar results have also been described in breast cancer (Lee et al, 2023; Li et al, 2023), pancreatic cancer (Yang et al, 2024a), head and neck squamous cell carcinoma (Zhu et al, 2024), and nasopharyngeal carcinoma (Zheng et al, 2023). Furthermore, VIRMA has been reported to be upregulated and amplified across many cancer entities, and its elevated expression has been associated with poor patient prognosis (Destefanis et al, 2024; Zhu et al, 2021). Our findings are in agreement with these studies and provide important mechanistic support for a functional role of VIRMA in UCB progression through m^6^A deposition.

However, the precise molecular mechanism underlying the association between m^6^A, its regulators, and polyadenylation factors remains unresolved, also given the inconsistent findings that were reported previously (Ke et al, 2015; Molinie et al, 2016; Ries et al, 2023; Yue et al, 2018). For example, it is currently unclear whether m^6^A directly modulates the recruitment or stability of polyadenylation factors or alters RNA structure to expose specific polyadenylation sites. This could be addressed by combining VIRMA or METTL3 interference with CLIP-seq of core polyadenylation factors and structure-probing approaches, like SHAPE-MaP (Siegfried et al, 2014) around (alternative) polyadenylation sites. The impact of VIRMA-dependent m^6^A on alternative polyadenylation could be investigated by integrating VIRMA and METTL3 perturbation models with 3′-end-targeted sequencing methods (Yu et al, 2020; Zheng et al, 2016), and/or reporter assays with mutated 3′-UTR m^6^A sites. Furthermore, a direct transcript-level assessment of how m^6^A methylation influences alternative polyadenylation would require robust multi-modal single-molecule sequencing approaches capable of simultaneously resolving m^6^A modifications and mRNA 3′-end usage. Current long-read sequencing technologies, including nanopore sequencing, are not yet ideally suited for this purpose due to their limited accuracy in m^6^A basecalling.

**Figure 7:**
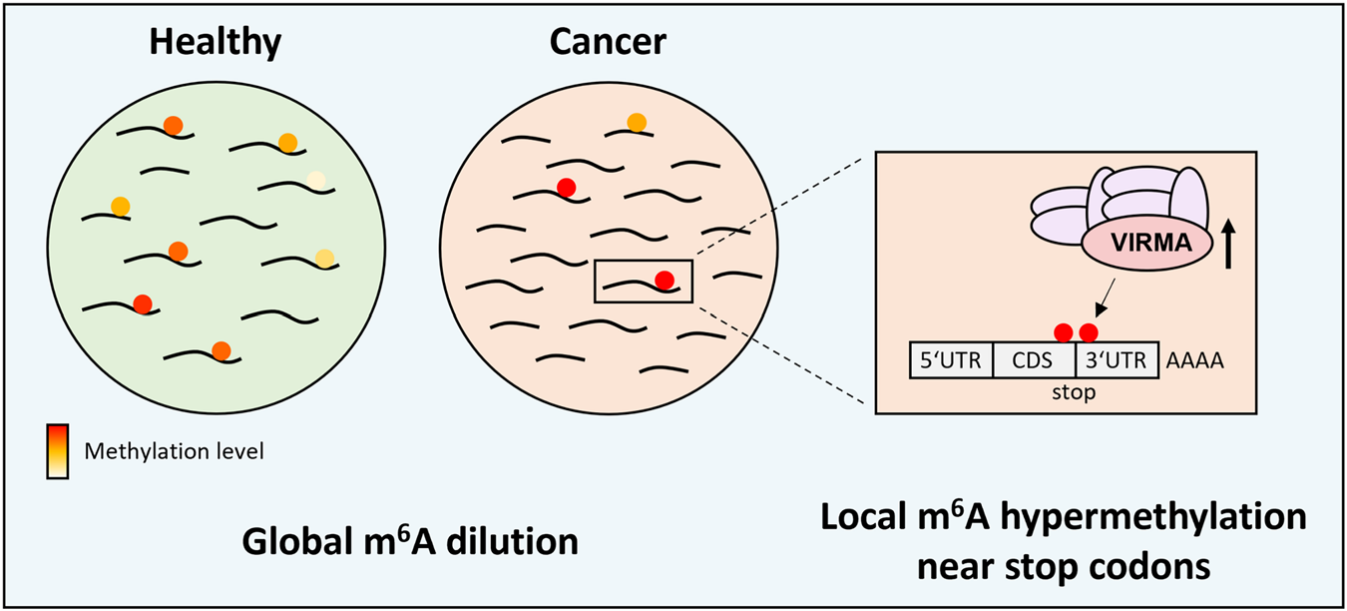
The m^6^A epitranscriptomic landscape of bladder cancer is characterized by global methylation dilution and focal 3’-UTR hypermethylation. Globally, the upregulation of unmethylated transcripts and the downregulation of highly methylated transcripts resulted in the dilution of m^6^A methylation levels in bladder cancer. Our findings further suggest that the upregulation of VIRMA causes the local hypermethylation of m^6^A modified target transcripts in regions close to the stop codon.

Altogether, our study identifies two separate signatures and mechanisms that alter the m^6^A epitranscriptomic landscape in cancer (Fig. 7). Global m^6^A methylation levels become diluted due to the upregulation of unmethylated transcripts and the downregulation of methylated transcripts, while focal m^6^A hypermethylation in 3’-UTR regions is associated with overexpression of VIRMA. The highly precise and quantitative results generated by GLORI were critical for uncovering these signatures. Further work will be needed to elucidate the potential of these signatures for identifying biomarkers for early detection of bladder cancer.

## Methods

**Table:** Reagents and tools table

Reagent/Resource	Reference or Source	Identifier or Catalog Number
**Experimental Models**		
HEK293T (*Homo sapiens*)	European Collection of Authenticated Cell Cultures (ECACC)	RRID: CVCL_0063
RT4 (*Homo sapiens*)	ECACC	RRID: CVCL_0036
T24 (*Homo sapiens*)	ECACC	RRID: CVCL_0554
UM-UC-3 (*Homo sapiens*)	ECACC	RRID: CVCL_1783
Human bladder tumor and paratumoral patient samples	Department of Urology and Urosurgery, Medical Faculty Mannheim	2015-549N-MA
**Recombinant DNA**		
psPAX	Addgene	Cat #12260
pMD2.G	Addgene	Cat #87360
pLentiCRISPR v2	Addgene	Cat #52961
**Antibodies**		
β-Actin monoclonal antibody	Sigma-Aldrich	Cat A5316
VIRMA polyclonal antibody	Proteintech	Cat 25712-1-AP
Donkey anti-rabbit IgG-HRP	Santa Cruz Biotechnology	Cat sc-2313
Donkey anti-mouse IgG-HRP	Thermo Fisher Scientific	Cat A16011
**Oligonucleotides and other sequence-based reagents**		
Anti-METTL3 sgRNA and anti-VIRMA shRNA sequences	This study and Horizon Discovery	Table EV6
**Chemicals, Enzymes and other reagents**		
RNAlater solution	Thermo Fisher Scientific	Cat AM7021
DMEM, high glucose, pyruvate medium	Gibco	Cat 41966052
McCoy’s 5A (Modified) medium	Gibco	Cat 26600023
Fetal bovine serum (FBS)	Gibco	Cat 10500064
Penicillin-Streptomycin (P/S)	Gibco	Cat 15140-122
TRIzol	Thermo Fisher Scientific	Cat 15596026
MEGAclear Transcription Clean-Up Kit	Thermo Fisher Scientific	Cat AM1908
Dynabeads mRNA Purification Kit	Thermo Fisher Scientific	Cat 61006
NEBNext Magnesium RNA Fragmentation Module	New England Biolabs	Cat E6150S
RNA Clean & Concentrator-5 kit (DNase I included)	Zymo Research	Cat R1013
Glyoxal solution	Sigma-Aldrich	Cat 50649
DMSO	AppliChem	Cat A3672,0250
Boric acid	Sigma-Aldrich	Cat B0394
Sodium nitrite	Thermo Fisher Scientific	Cat 15633430
MES	Thermo Fisher Scientific	Cat J60763.AP
Ethanol	Sigma-Aldrich	Cat 32205-M
Triethylammonium acetate	Thermo Fisher Scientific	Cat 90358
Deionized formamide	Roth	Cat P040.1
Antarctic phosphatase	New England Biolabs	Cat M0289S
T4 Polynucleotide Kinase	New England Biolabs	Cat M0201S
NEBNext Small RNA Library Prep Set for Illumina	New England Biolabs	Cat E7330S
NEBNext Multiplex Oligos for Illumina (Index Primer Sets 1 and 3)	New England Biolabs	Cat E7335S and E7710S
8% TBE polyacrylamide gels	Thermo Fisher Scientific	Cat EC6215BOX
Lipofectamine 2000	Thermo Fisher Scientific	11668019
STM2457	MedChemExpress	Cat HY-134836
Tris-HCl	Sigma-Aldrich	Cat T1503
Sodium chloride	Thermo Fisher Scientific	Cat 15855188
EDTA	Gerbu	Cat 1034
Triton X-100	Sigma-Aldrich	Cat X100
Complete Protease Inhibitor Cocktail	Roche	Cat 11697498001
Trans-Blot Turbo Transfer Pack	Bio-Rad	Cat 1704158
Milk powder	Gerbu	Cat 1602.0500
Immobilon Western HRP Substrate	Merck	Cat WBKLS0500
Cell Titer-Glo Luminescent Cell Viability Assay kit	Promega	Cat G7571
Caspase-Glo 3/7 Assay kit	Promega	Cat G8091
Methanol	Thermo Fisher Scientific	M-4000-PC17
Crystal violet	Sigma-Aldrich	C3886
**Software**		
Trim Galore	https://github.com/FelixKrueger/TrimGalore	version 0.6.6
GLORI-tools	Liu et al, 2023	version 1.0
Python		version 3.10.1
Samtools	Li et al, 2009	version 1.19
STAR	Dobin et al, 2013	version 2.7.10a
Bowtie	Langmead et al, 2009	version 1.3.0
bedtools getfasta	Quinlan and Hall, 2010	version 2.25.0
HOMER	Heinz et al, 2010	version 4.11
DiffLogo	Nettling et al, 2015	version 2.20.0
methylSig	Park et al, 2014	version 1.7.0
ShinyGO	Ge et al, 2020	version 0.81
HISAT2	Kim et al, 2019	version 2.2.1
featureCounts	Liao et al, 2013	version 2.0.6
DESeq2	Love et al, 2014	version 1.42.0
DaPars	Xia et al, 2014	version 1.0.0
Lifelines	https://joss.theoj.org/papers/10.21105/joss.01317	version 0.30.0
ImageJ	https://imagej.nih.gov/ij/index.html	
ColonyArea	Guzmán et al, 2014	
**Other**		
TissueRuptor II	Qiagen	
2200 TapeStation	Agilent Technologies	
Thermocycler T3000	Biometra	
NovaSeq 6000	Illumina	
Trans-Blot Turbo Transfer System	Bio-Rad	
M6 ECL Chemostar fluorescence imaging system	Intas	
GloMax Explorer Multimode Microplate Reader	Promega	

### Urothelial carcinoma patients and sample acquisition

This study was performed in adherence to the Declaration of Helsinki. All patients provided informed consent to participate in the molecular characterization of their tissue samples. Additionally, approval of the institutional ethics review board (Ethical Committee II, University of Heidelberg, Germany, reference number: 2015-549N-MA) was taken. Patient information is provided in Table EV4.

Nine tumor samples were obtained from transurethral resection of the bladder (TURB) specimens. Nine independent paratumoral control tissues were taken from cystectomy specimens. Samples were diagnosed by an uropathologist, and tumors were characterized according to the TNM classification for bladder cancer by the Union for International Cancer Control (UICC 2017). Bladder tumors with variant histopathological findings other than urothelial carcinoma were excluded. Patient tissue samples were stored at -20 °C in RNAlater solution until further processing.

### Cell culture

Cell lines were authenticated by single nucleotide polymorphism-profiling, tested for mycoplasma and cultured based on ATCC guidelines. HEK293T (RRID: CVCL_0063) and UM-UC-3 (RRID: CVCL_1783) cell lines were cultured in DMEM high glucose medium supplemented with 10% FBS and 1% P/S. RT4 (RRID: CVCL_0036) and T24 (RRID: CVCL_0554) cell lines were cultured in McCoy’s 5 A (modified) medium supplemented with 10% FBS and 1% P/S. All cell lines were cultivated as adherent monolayers at 37 °C in a humidified incubator with an atmosphere of 5% CO_2_.

### Isolation and preparation of RNA samples for GLORI-sequencing

RNA isolation and preparation for GLORI were performed as described (Liu et al, 2023; Shen et al, 2024). Patient tissue samples were homogenized by disruption using the TissueRuptor II (Qiagen). Total RNA from homogenized tissue samples and HEK293T cells was isolated with TRIzol. Small RNA fractions were depleted using the MEGAclear Transcription Clean-Up Kit (Thermo Fisher Scientific). Enrichment of mRNA was performed twice using the Dynabeads mRNA Purification Kit (Thermo Fisher Scientific). mRNA was then fragmented by incubation for 3 min at 94 °C using the NEBNext Magnesium RNA Fragmentation Module (New England Biolabs). The fragmentation step was performed differently from the published version of the protocol which described mRNA fragmentation for 4 min at 94 °C. Fragmented mRNA was then DNase I-treated and purified by the RNA Clean & Concentrator-5 kit (Zymo Research). The concentration as well as the average and peak mRNA fragment lengths were determined by TapeStation (Agilent Technologies).

### Protection, deamination, and deprotection of RNA samples for GLORI-sequencing

All steps were performed as described (Liu et al, 2023; Shen et al, 2024). For RNA protection, 100–200 ng of fragmented mRNA supplemented with a synthetic Spike-In RNA oligonucleotide were added into protection buffer (1.32 M Glyoxal solution, 50% DMSO, prepared in water) and incubated for 30 min at 50 °C in a thermal cycler. The sequence of the Spike-In RNA oligonucleotide is listed in Table EV6. The RNA was further stabilized by adding 10 µL of freshly prepared saturated boric acid at RT, followed by incubation for 30 min at 50 °C in a thermal cycler. Protected RNA was then added into 50 µL of freshly prepared deamination buffer (1.5 M NaNO_2_, 80 mM MES (pH 6.0), 1.76 M Glyoxal solution, prepared in water), and incubated for 8 h at 16 °C in a thermal cycler. After deamination, RNA was purified by ethanol precipitation overnight at −80 °C. Pelleted RNA was washed twice using 75% ethanol and air-dried for 5 min at RT. Then, the RNA pellet was resuspended in 50 µL of deprotection buffer (500 mM triethylammonium acetate (pH 8.6), 47.5% deionized formamide, prepared in water) and incubated for 10 min at 95 °C in a thermal cycler. Deprotected RNA was purified by ethanol precipitation for at least 30 min at −80 °C. Pelleted RNA was washed twice using 75% ethanol and air-dried for 5 min at RT. The RNA pellet was then resuspended in 50 µL of water and further purified using the RNA Clean & Concentrator-5 kit (Zymo Research).

### Preparation of GLORI libraries and sequencing

For sequencing library preparation, a two-step RNA end-repair protocol was used. RNA 3’-end dephosphorylation was performed by Antarctic phosphatase (New England Biolabs) treatment in a total reaction volume of 20 µL. The reaction was incubated for 30 min at 37 °C and inactivated for 2 min at 80 °C. Subsequently, RNA 5’-end phosphorylation was performed by T4 Polynucleotide Kinase (New England Biolabs) treatment in a total reaction volume of 50 µL. The reaction was incubated for 30 min at 37 °C, inactivated for 20 min at 65 °C, and purified using the RNA Clean & Concentrator-5 kit (Zymo Research). GLORI-sequencing libraries were then prepared using the NEBNext Small RNA Library Prep Set for Illumina in combination with the NEBNext Multiplex Oligos for Illumina (Index Primer Sets 1 and 3) (New England Biolabs). Size selection of the libraries was performed via TBE-PAGE using 8% TBE polyacrylamide gels (Thermo Fisher Scientific) selecting sequencing competent molecules in a size range of 160–250 bp. Average peak size and concentration of the libraries were determined by TapeStation (Agilent Technologies). Libraries were sequenced on a NovaSeq 6000 platform (Illumina) applying a 100 bp paired-end sequencing protocol. Sequencing was performed by the Next Generation Sequencing Core Facility of the German Cancer Research Center, Heidelberg. Raw sequencing data were then trimmed using Trim Galore (version 0.6.6, https://github.com/FelixKrueger/TrimGalore) and further processed by the GLORI-tools pipeline as described (Liu et al, 2023; Shen et al, 2024). GLORI-tools is available on GitHub: https://github.com/liucongcas/GLORI-tools. Parameters for the selection of m^6^A sites were set as follows: ≥5 variant nucleotides, ≥15 coverage of A and G bases, ≥0.1 A rate (methylation level >10%). All steps of the GLORI-tools pipeline were executed using the following software: python (version 3.10.1), samtools (version 1.19 (Li et al, 2009)), STAR (version 2.7.10a (Dobin et al, 2013)), bowtie (version 1.3.0 (Langmead et al, 2009)). The human genome (GRCh38) and transcriptome (GCF_000001405.39) reference files were downloaded from UCSC.

### Motif analysis

The coordinates and flanking sequences of GLORI-derived m^6^A sites were extracted using bedtools getfasta (version 2.25.0 (Quinlan and Hall, 2010)) and the human genome reference file (GRCh38). Sequence motifs were then identified using Hypergeometric Optimization of Motif EnRichment (HOMER, version 4.11 (Heinz et al, 2010)). For visualization, the motif representations were converted into position weight matrices using DiffLogo (version 2.20.0 (Nettling et al, 2015)).

### Differential methylation analysis

The R package methylSig (version 1.7.0 (Park et al, 2014)) was used to identify differentially methylated sites comparing control and cancer tissues. For the analysis, an absolute methylation level cutoff of 10% and a *p*-value cutoff of 0.05 were selected. The statistical significance was determined using beta binomial models. Gene ontology analyses were performed using ShinyGO (version 0.81 (Ge et al, 2020)).

### Bulk RNA-sequencing

Total RNA from tissue samples and cell lines was isolated using TRIzol. To remove residual gDNA contaminants, total RNA samples were DNase I-digested and further purified using the RNA Clean & Concentrator-5 kit (Zymo Research). Library preparation and sequencing were performed by the Next Generation Sequencing Core Facility of the German Cancer Research Center, Heidelberg. The samples were sequenced on a NovaSeq 6000 platform (Illumina) applying a 100 bp paired-end sequencing protocol. Reads were trimmed using Trim Galore (version 0.6.6) and mapped to the human reference genome (GRC38) using HISAT2 (version 2.2.1 (Kim et al, 2019)).

### Differential gene expression analysis

Aligned reads from bulk RNA-sequencing experiments were counted by the featureCounts function (2.0.6 (Liao et al, 2013)), normalized, and differential gene expression analysis performed via DESeq2 (version 1.42.0 (Love et al, 2014)). Only transcripts with a read count >10 in every sample were included in downstream analyses.

### Weighted global methylation level analysis

For the determination of the weighted global methylation level in the individual samples, m^6^A methylation data from GLORI-sequencing and transcript expression data from RNA-sequencing were integrated. The methylation level of each transcript was aggregated and then multiplied by the TPM-normalized expression level of the same transcript. These individual, weighted methylation levels were then summed up to compute the weighted global methylation level.

### Analysis of alternative polyadenylation events

For the de novo identification of alternative polyadenylation sites, aligned reads from bulk RNA-sequencing experiments were analyzed using the DaPars algorithm, version 1.0.0 (Xia et al, 2014). For the analyses, a coverage cutoff of 30, an FDR cutoff of 0.05, an absolute PDUI difference cutoff of 0.1, and an absolute fold change cutoff of 0.59 were selected.

### Establishment of T24 and UM-UC-3 METTL3 knockout clones

HEK293T cells were transfected with the lentiviral packaging vectors psPAX (Plasmid #12260, Addgene) and pMD2.G (Plasmid #87360, Addgene) as well as the pLentiCRISPR v2 vector (Plasmid #52961, Addgene) using Lipofectamine 2000 (Thermo Fisher Scientific). Anti-METTL3 and scramble sgRNA sequences are listed in Table EV6. Transfected HEK293T cells were incubated for 48 h. T24 and UM-UC-3 cells were transduced for 48 h and further cultivated for the establishment of clonal populations.

### STM2457 treatment of urothelial carcinoma cells

T24 and UM-UC-3 cells were cultured until reaching a confluency of approximately 80%. The cells were then treated for 48 h with DMSO or 50 µM of the METTL3 inhibitor STM2457 (MedChemExpress).

### Databank VIRMA expression analysis

VIRMA mRNA expression data were downloaded from the UCSC database for the combined The Cancer Genome Atlas bladder urothelial carcinoma (TCGA-BLCA) cohort and the Genotype-Tissue Expression (GTEx) project (https://xenabrowser.net/). Expression data were DESeq2-normalized and log_2_-transformed (log_2_(value + 1)). Mann–Whitney U test was assessed to test for statistical significance between control and UCB tissues.

### Association analysis of VIRMA genomic alterations and expression

Genetic alteration data and the corresponding mRNA expression data for VIRMA were downloaded from the cBioPortal database (https://www.cbioportal.org/) for TCGA-BLCA cohort. VIRMA mRNA expression values were preprocessed from RNA-sequencing by Expectation-Maximization (RSEM) data generated by the TCGA RNASeqV2 pipeline (Illumina HiSeq), which were batch-normalized and log_2_-transformed (log_2_(value + 1)). Different copy-number alterations (shallow deletion, diploid, gain, amplification) were identified based on the Genomic Identification of Significant Targets in Cancer (GISTIC) method. Pearson correlation analysis was performed to assess the association between log_2_ copy-number values and VIRMA mRNA expression levels.

### Survival analysis

Kaplan–Meier overall survival analysis was performed using the lifelines python package. Patients were stratified into VIRMA-high and VIRMA-low mRNA expression groups using maximally selected log-rank statistics.

### Establishment of VIRMA knockdown cell lines

Lentiviral shRNA constructs for VIRMA KD were purchased from Horizon Discovery (clone_96736 (RHS4430-200156255) and clone_96733 (RHS4430-200172168). The sequences of the anti-VIRMA shRNAs are listed in Table EV6. Sequence information for the non-targeting shRNA control was not provided by the manufacturer. Transfection and transduction were performed as described for the establishment of METTL3 KO cells.

### Western blot

Cells were lysed in 20 mM Tris-HCl (pH 7.5), 150 mM NaCl, 1 mM EDTA and 1% Triton X-100 supplemented with the Complete Protease Inhibitor Cocktail (Roche, Basel, Switzerland). Proteins were then separated by SDS-PAGE and transferred to nitrocellulose membranes using a Trans-Blot Turbo Transfer System (Bio-Rad). Membranes were blocked in 0.1% PBST containing 5% milk powder for 1 h at room temperature. Primary antibody incubation was performed overnight at 4 °C using the β-Actin monoclonal antibody (Sigma, A5316) and the VIRMA polyclonal antibody (Proteintech, 25712-1-AP). Secondary antibody incubation occurred for 1 h at room temperature. Finally, membranes were imaged using the Immobilon Western HRP Substrate (Merck), and signals detected using an M6 ECL Chemostar fluorescence imaging system (Intas).

### Cancer cell phenotypic assays

For cell proliferation assays, VIRMA KD and shCtrl cells were seeded in 96-well plates with the following densities: UM-UC-3 cells: 1000 cells/well. RT4 cells: 1500 cells/well. Cell proliferation was quantified by Cell Titer-Glo (Promega) measurements for 5 consecutive days in time intervals of 24 h. Two-way analysis of variance was used to test for statistical significance between VIRMA KD and shCtrl cells. For colony formation assays, VIRMA KD and shCtrl cells were seeded in 6-well plates with the following densities: UM-UC-3 cells: 500 cells/well. RT4 cells: 1000 cells/well. Then, cells were incubated for 1.5 weeks and fixed with ice-cold methanol for 10 min. Staining was conducted using a 0.5% crystal violet solution for 10 min at room temperature. Colonies were counted using the ImageJ plugin “ColonyArea” (Guzmán et al, 2014). Two-tailed Student’s t tests were used to test for statistical significance between VIRMA KD and shCtrl cells. For apoptosis assays, VIRMA KD and shCtrl cells were seeded in 96-well plates with 10,000 cells/well. Caspase activity was quantified by the Caspase-Glo 3/7 Assay kit (Promega) 24 h after seeding. Also, a Cell Titer-Glo (Promega) measurement was conducted to determine the number of cells for normalization. Two-tailed Student’s t tests were used to test for statistical significance between VIRMA KD and shCtrl cells.

## Supplementary information

Table EV1

Table EV2

Table EV3

Table EV4

Table EV5

Table EV6

Peer Review File

Source data Fig. 6

Figure EV8 Source Data

Expanded View Figures

## Data Availability

Data generated in this study have been deposited in the GEO database under the accession numbers GSE281749 and GSE281750. The source data of this paper are collected in the following database record: biostudies:S-SCDT-10_1038-S44319-026-00739-y.
