# Supplementary material for: The bladder cancer m6A landscape is defined by global methylation dilution and focal 3′-UTR hypermethylation
Source: EMBO Rep. 2026 Mar 23;27(8):2118–43. doi: 10.1038/s44319-026-00739-y (PMC13121636; doi:10.1038/s44319-026-00739-y)
Supplement: Supplementary file 1 — Table EV1 [file 44319_2026_739_MOESM1_ESM.docx]

**Table EV1. Seqencing yield and mapping efficiencies for samples sequenced by GLORI.**

| Sample | Number of input reads | Mapping efficiency [%] |
| --- | --- | --- |
| HEK293T I | 300922280 | 52.18 |
| HEK293T II | 222200609 | 50.84 |
| HEK293T III | 111741441 | 51.8 |
| Paratumoral I | 210134684 | 50.05 |
| Paratumoral II | 114142354 | 50.9 |
| Paratumoral III | 194060112 | 50.95 |
| Paratumoral IV | 202742613 | 50.41 |
| Paratumoral V | 252247624 | 48.63 |
| Paratumoral VI | 163084610 | 48.56 |
| Paratumoral VII | 210997476 | 49.52 |
| Paratumoral VIII | 149997503 | 50.07 |
| Paratumoral IX | 125859823 | 49.83 |
| RT4 shCtrl I | 307603811 | 50.85 |
| RT4 shCtrl II | 345624521 | 51.16 |
| RT4 shCtrl III | 301014538 | 50.67 |
| RT4 VIRMA KD I | 273857674 | 47 |
| RT4 VIRMA KD II | 192310642 | 52.12 |
| RT4 VIRMA KD III | 169403065 | 50.71 |
| T24 DMSO I | 260940137 | 46.67 |
| T24 DMSO II | 309220350 | 45.85 |
| T24 DMSO III | 266191689 | 46.28 |
| T24 STM2457 I | 233509215 | 46.2 |
| T24 STM2457 II | 137268107 | 46.94 |
| T24 STM2457 III | 180422209 | 46.18 |
| UCB I | 224895670 | 49.42 |
| UCB II | 230506398 | 49.71 |
| UCB III | 207737049 | 50.33 |
| UCB IV | 269625826 | 51.4 |
| UCB V | 301150528 | 50.86 |
| UCB VI | 292801112 | 51.33 |
| UCB VII | 546199419 | 46.21 |
| UCB VIII | 104346379 | 48.29 |
| UCB IX | 179928871 | 47.86 |
| UM-UC-3 shCtrl I | 269840589 | 50.25 |
| UM-UC-3 shCtrl II | 204550065 | 51.12 |
| UM-UC-3 shCtrl III | 193087828 | 51.22 |
| UM-UC-3 VIRMA KD I | 210084132 | 51.20 |
| UM-UC-3 VIRMA KD II | 144803795 | 51.27 |
| UM-UC-3 VIRMA KD III | 127291683 | 51.20 |
