# Supplementary material for: The bladder cancer m6A landscape is defined by global methylation dilution and focal 3′-UTR hypermethylation
Source: EMBO Rep. 2026 Mar 23;27(8):2118–43. doi: 10.1038/s44319-026-00739-y (PMC13121636; doi:10.1038/s44319-026-00739-y)
Supplement: Supplementary file 2 — Table EV2 [file 44319_2026_739_MOESM2_ESM.docx]

**Table EV2: Conversion rates for samples sequenced by GLORI.**

| Sample | Loci | Median conversion [%] |
| --- | --- | --- |
| HEK293T I | Spike-in | 99 |
|  | All | 100 |
| HEK293T II | Spike-in | 99 |
|  | All | 100 |
| HEK293T III | Spike-in | 99 |
|  | All | 100 |
| Paratumoral I | Spike-in | 99.5 |
|  | All | 100 |
| Paratumoral II | Spike-in | 100 |
|  | All | 98.9 |
| Paratumoral III | Spike-in | 99.5 |
|  | All | 98.9 |
| Paratumoral IV | Spike-in | 99 |
|  | All | 100 |
| Paratumoral V | Spike-in | 99 |
|  | All | 100 |
| Paratumoral VI | Spike-in | 99 |
|  | All | 100 |
| Paratumoral VII | Spike-in | 99 |
|  | All | 100 |
| Paratumoral VIII | Spike-in | 99 |
|  | All | 100 |
| Paratumoral IX | Spike-in | 99 |
|  | All | 100 |
| RT4 shCtrl I | Spike-in | 98.2 |
|  | All | 99.3 |
| RT4 shCtrl II | Spike-in | 98.3 |
|  | All | 99.2 |
| RT4 shCtrl III | Spike-in | 98.4 |
|  | All | 99.3 |
| RT4 VIRMA KD I | Spike-in | 98.4 |
|  | All | 99.4 |
| RT4 VIRMA KD II | Spike-in | 98.4 |
|  | All | 99.2 |
| RT4 VIRMA KD III | Spike-in | 98.4 |
|  | All | 99.6 |
| T24 DMSO I | Spike-in | 98.8 |
|  | All | 100 |
| T24 DMSO II | Spike-in | 98.8 |
|  | All | 100 |
| T24 DMSO III | Spike-in | 98.9 |
|  | All | 100 |
| T24 STM2457 I | Spike-in | 98.9 |
|  | All | 100 |
| T24 STM2457 II | Spike-in | 98.8 |
|  | All | 100 |
| T24 STM2457 III | Spike-in | 99 |
|  | All | 100 |
| UCB I | Spike-in | 99.5 |
|  | All | 98.9 |
| UCB II | Spike-in | 99.5 |
|  | All | 98.9 |
| UCB III | Spike-in | 99.5 |
|  | All | 98.9 |
| UCB IV | Spike-in | 99.5 |
|  | All | 98.9 |
| UCB V | Spike-in | 99.5 |
|  | All | 99 |
| UCB VI | Spike-in | 99.4 |
|  | All | 98.9 |
| UCB VII | Spike-in | 99 |
|  | All | 100 |
| UCB VIII | Spike-in | 99 |
|  | All | 100 |
| UCB IX | Spike-in | 99 |
|  | All | 100 |
| UM-UC-3 shCtrl I | Spike-in | 98.4 |
|  | All | 99.2 |
| UM-UC-3 shCtrl II | Spike-in | 98.2 |
|  | All | 99 |
| UM-UC-3 shCtrl III | Spike-in | 98.2 |
|  | All | 98.8 |
| UM-UC-3 VIRMA KD I | Spike-in | 98.4 |
|  | All | 99 |
| UM-UC-3 VIRMA KD II | Spike-in | 98.3 |
|  | All | 99 |
| UM-UC-3 VIRMA KD III | Spike-in | 98.5 |
|  | All | 99.2 |
