# Supplementary material for: The bladder cancer m6A landscape is defined by global methylation dilution and focal 3′-UTR hypermethylation
Source: EMBO Rep. 2026 Mar 23;27(8):2118–43. doi: 10.1038/s44319-026-00739-y (PMC13121636; doi:10.1038/s44319-026-00739-y)
Supplement: Supplementary file 3 — Table EV3 [file 44319_2026_739_MOESM3_ESM.docx]

**Table EV3: Fraction of GLORI-sequenced reads from T24 cells mapping to different transcriptomic features.** featureCounts was used to map and count aligned sequencing reads to transcriptomic coordinates.

| Feature | Average read count | Percentage [%] |
| --- | --- | --- |
| protein-coding | 119,588,441 | 85.4 |
| pseudogene (all types) | 17,502,095.3 | 12.5 |
| long non-coding RNA (lncRNA) | 2,791,594.3 | 2.0 |
| (small) non-coding RNA | 77,803 | < 0.1 |
| TEC (to be experimentally confirmed) | 66,698.7 | < 0.1 |
| ribosomal RNA (rRNA) | 5,804.3 | < 0.01 |
| Immunoglobulin/T-cell receptor genes | 3,510.7 | < 0.01 |
| artifact / low-confidence | 2,650 | < 0.01 |
| mitochondrial RNA (mtRNA) | 0 | 0 |
