# Supplementary material for: The bladder cancer m6A landscape is defined by global methylation dilution and focal 3′-UTR hypermethylation
Source: EMBO Rep. 2026 Mar 23;27(8):2118–43. doi: 10.1038/s44319-026-00739-y (PMC13121636; doi:10.1038/s44319-026-00739-y)
Supplement: Supplementary file 4 — Table EV4 [file 44319_2026_739_MOESM4_ESM.docx]

**Table EV4: Clinicopathological data from the cohort of 18 patients.** Urothelial carcinoma of the bladder tissue samples were obtained by transurethral resection of a bladder (TURB). Non-malignant uroepithelial tissue samples were obtained by radical cystectomy. All samples were collected at the Department of Urology and Urosurgery at the University Hospital of Mannheim.

| Clinical data | Cohort (n=18) |
| --- | --- |
| Age |  |
| mean | 76.9 |
| min-max | 71-85 |
| Tissue |  |
| n (uroepithelial) | 9 |
| n (tumoral) | 9 |
| T-Stage |  |
| Ta | 6 |
| T1 | 0 |
| T2 | 3 |
| T3 | 0 |
| T4 | 0 |
