# Supplementary material for: The bladder cancer m6A landscape is defined by global methylation dilution and focal 3′-UTR hypermethylation
Source: EMBO Rep. 2026 Mar 23;27(8):2118–43. doi: 10.1038/s44319-026-00739-y (PMC13121636; doi:10.1038/s44319-026-00739-y)
Supplement: Supplementary file 5 — Table EV5 [file 44319_2026_739_MOESM5_ESM.docx]

**Table EV5: Within-group variability analysis of detected DRAC sites.** The numbers show that methylation levels in each sample group fluctuate by 1.2%. Additionally, 90% of all sites vary less than 6% in SD across replicates. On average, the variation between the highest and lowest methylation level is 3-4% across replicates. Therefore, between-group differences ≥ 10% exceed normal variability within each group.

| Group | Number of DRAC sites | Mean SD [%] | P90 SD [%] | Mean range [%] |
| --- | --- | --- | --- | --- |
| UCB | 353,348 | 1.21 | 5.92 | 3.53 |
| Control | 353,348 | 1.15 | 5.52 | 3.32 |
