# Supplementary material for: The bladder cancer m6A landscape is defined by global methylation dilution and focal 3′-UTR hypermethylation
Source: EMBO Rep. 2026 Mar 23;27(8):2118–43. doi: 10.1038/s44319-026-00739-y (PMC13121636; doi:10.1038/s44319-026-00739-y)
Supplement: Supplementary file 6 — Table EV6 [file 44319_2026_739_MOESM6_ESM.docx]

**Table EV6: Sequence and source information for oligos, sgRNAs and shRNAs used in this study.**

| Oligo / sgRNA / shRNA | Sequence (5’→3’) | Source, Identifier |
| --- | --- | --- |
| Spike-In RNA (GLORI) | AGCGCAGCGAACAUGACACGUGC  UCAACAGUGUAGUUGGACUUCCU  CGCAUCUAGCCGCUAUGCACGUG  CAGCU | Liu *et al*., 2023 |
| Anti-METTL3 sgRNA1_F | AGACTAGGATGTCGGACACG | Koch *et al*., 2023 |
| Anti-METTL3 sgRNA1_R | CGTGTCCGACATCCTAGTCT | Koch *et al*., 2023 |
| Anti-METTL3 sgRNA2_F | CTGGTGGCCCTAAGCCCAGC | Koch *et al*., 2023 |
| Anti-METTL3 sgRNA2_R | GCTGGGCTTAGGGCCACCAG | Koch *et al*., 2023 |
| Anti-METTL3 sgRNA3_F | ATGCTGACCATTCCAAGCTC | Koch *et al*., 2023 |
| Anti-METTL3 sgRNA3_R | GAGCTTGGAATGGTCAGCAT | Koch *et al*., 2023 |
| Anti-METTL3 sgRNA4_F | AAGTGCAAGAATTCTGTGAC | Koch *et al*., 2023 |
| Anti-METTL3 sgRNA4_R | GTCACAGAATTCTTGCACTT | Koch *et al*., 2023 |
| Non-targeting Scr sgRNA_F | GCTGACGGCGAGCTTTAGGC | Koch *et al*., 2023 |
| Non-targeting Scr sgRNA_R | GCCTAAAGCTCGCCGTCAGC | Koch *et al*., 2023 |
| Anti-VIRMA shRNA1 | TTCTTCTAAAGCTGTTACC | Horizon Discovery, clone_96736 (RHS4430-200156255) |
| Anti-VIRMA shRNA2 | ATTCTCATCATATTCCAGG | Horizon Discovery, clone_96733 (RHS4430-200172168) |
