# Supplementary figures and images for: The bladder cancer m6A landscape is defined by global methylation dilution and focal 3′-UTR hypermethylation
Source: EMBO Rep. 2026 Mar 23;27(8):2118–43. doi: 10.1038/s44319-026-00739-y (PMC13121636; doi:10.1038/s44319-026-00739-y)

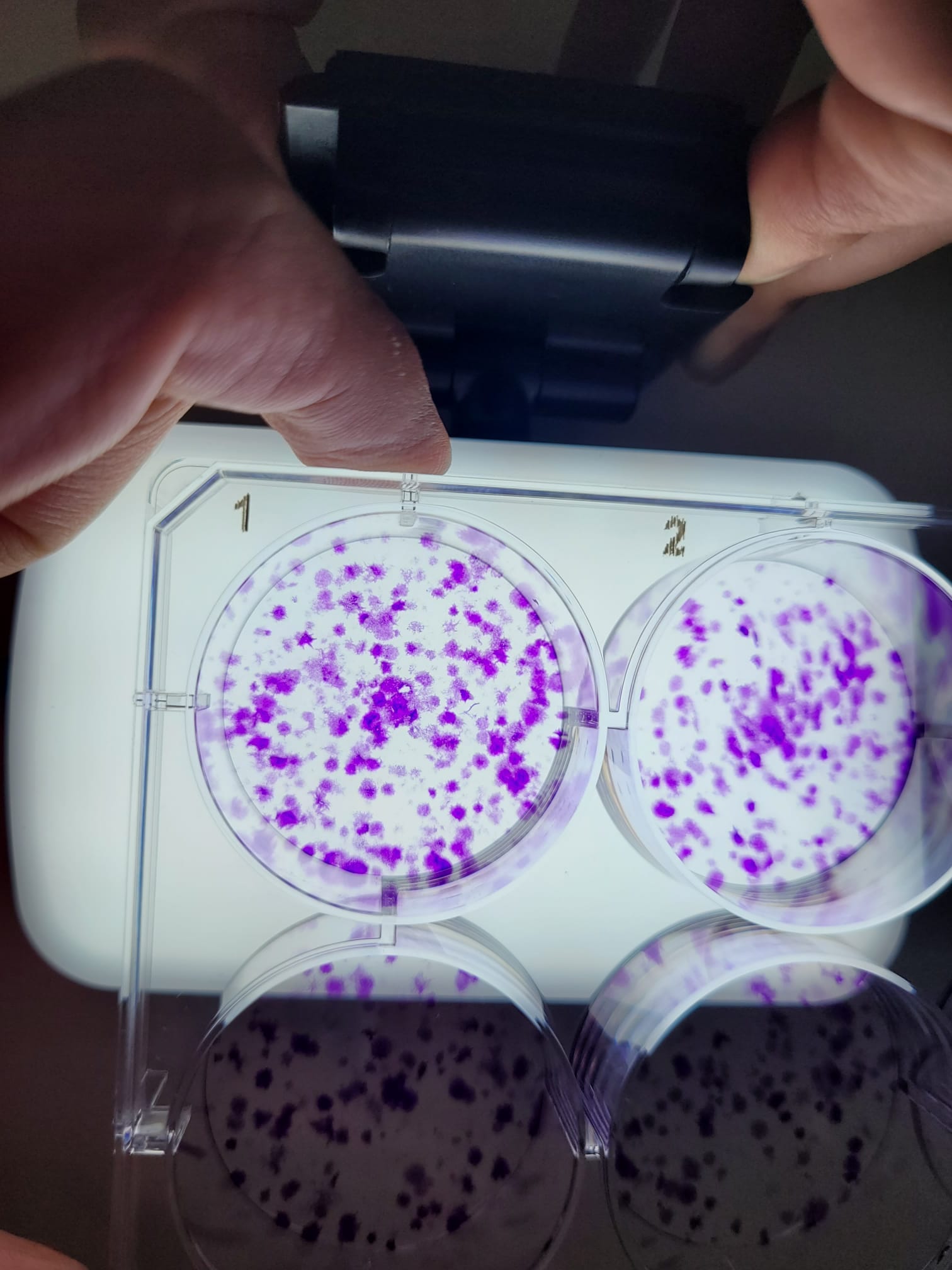

Supplement: Supplementary file 8 — Source data Fig. 6 [file 44319_2026_739_MOESM8_ESM.zip › Figure 6 Panel G/ctrl1.jpeg]

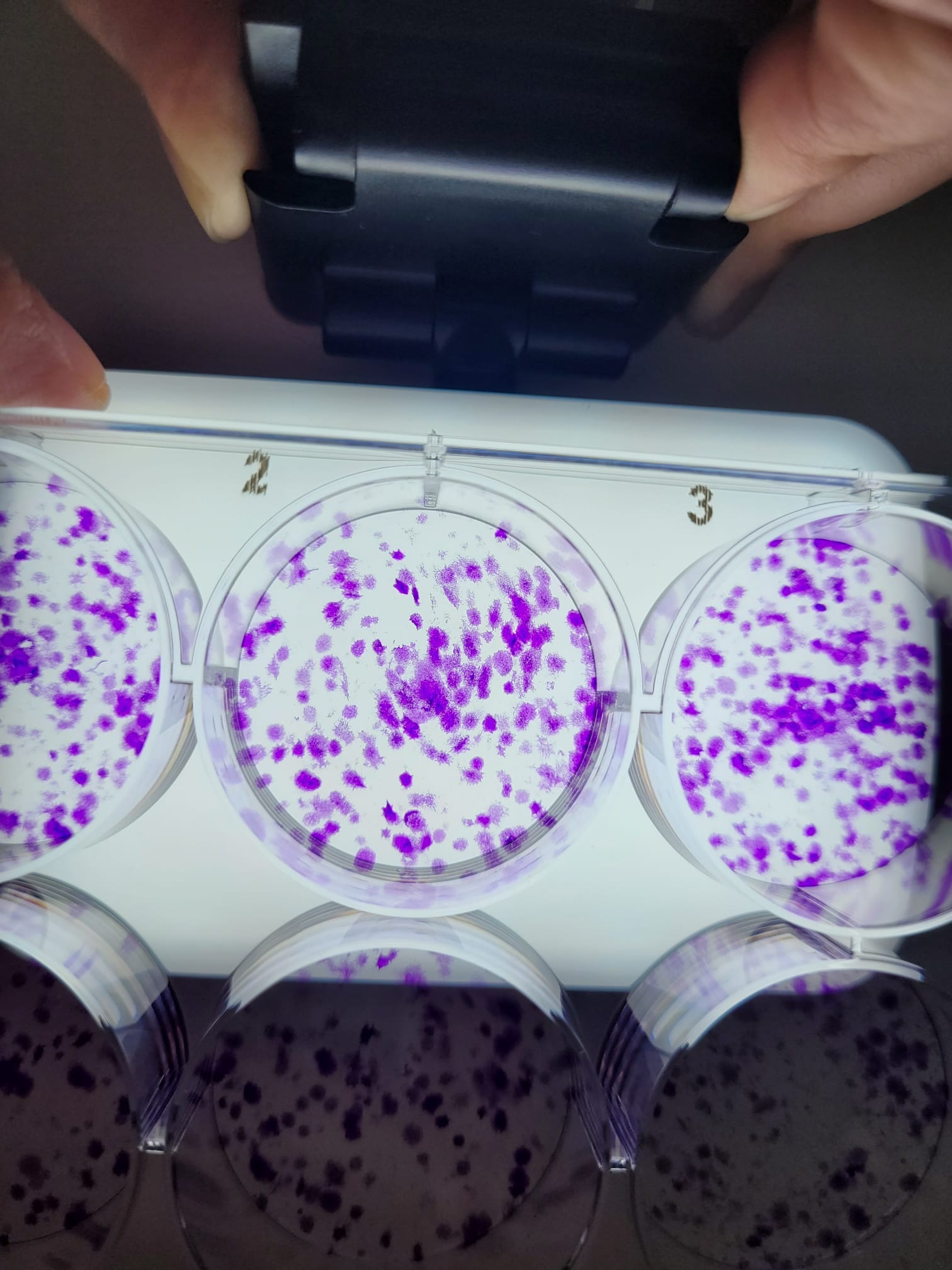

Supplement: Supplementary file 8 — Source data Fig. 6 [file 44319_2026_739_MOESM8_ESM.zip › Figure 6 Panel G/ctrl2.jpeg]

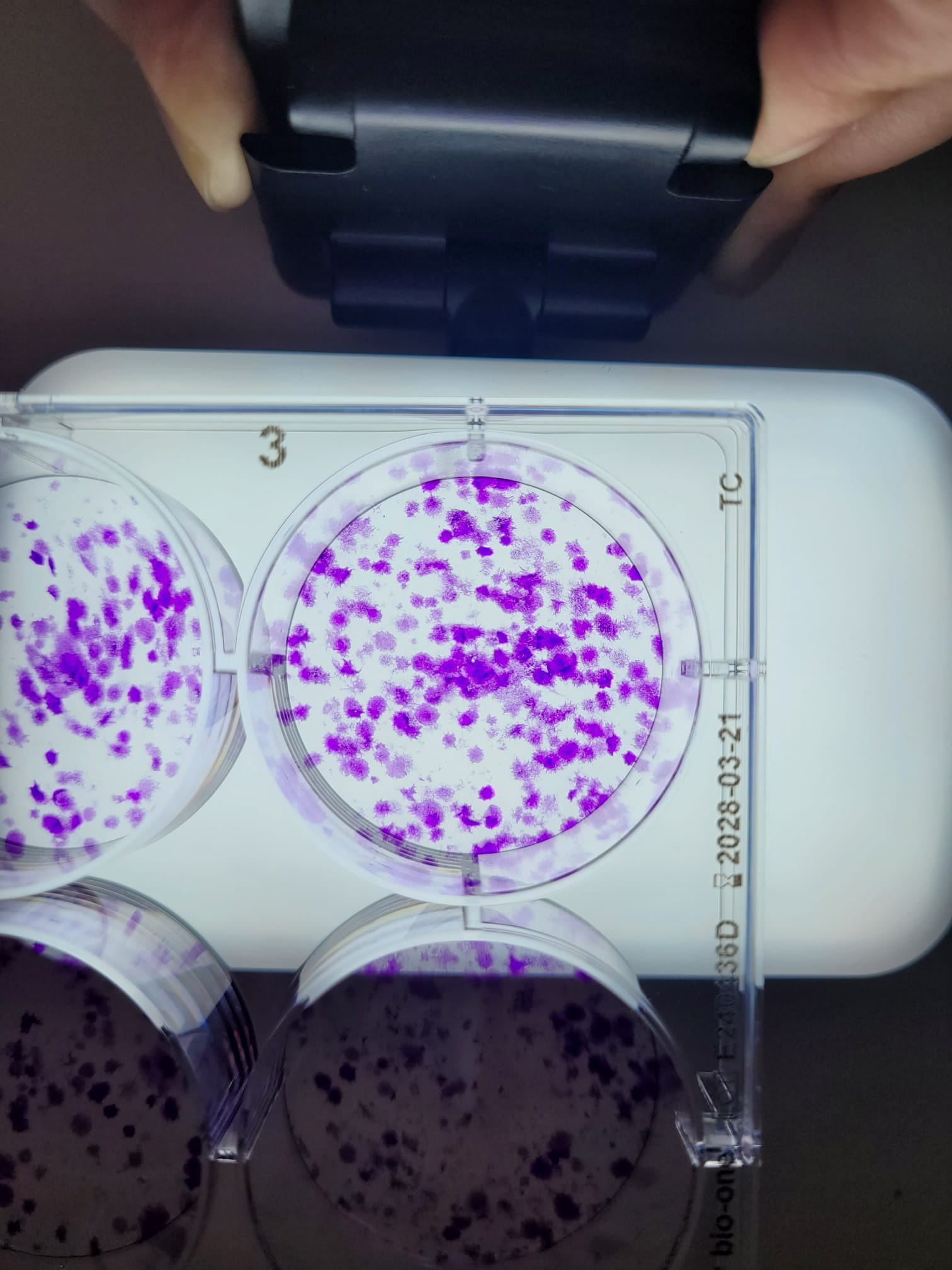

Supplement: Supplementary file 8 — Source data Fig. 6 [file 44319_2026_739_MOESM8_ESM.zip › Figure 6 Panel G/ctrl3.jpeg]

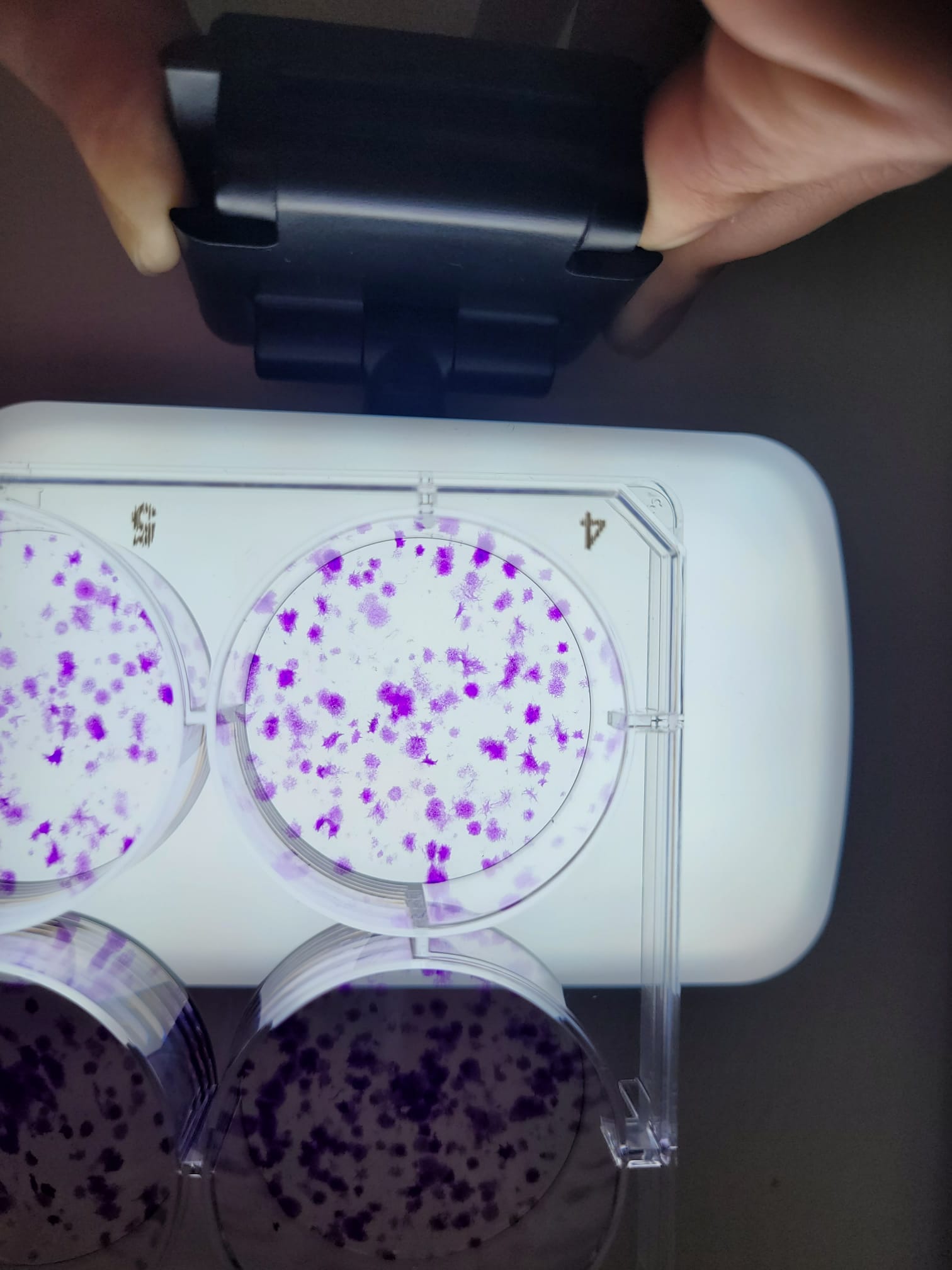

Supplement: Supplementary file 8 — Source data Fig. 6 [file 44319_2026_739_MOESM8_ESM.zip › Figure 6 Panel G/sh1_1.jpeg]

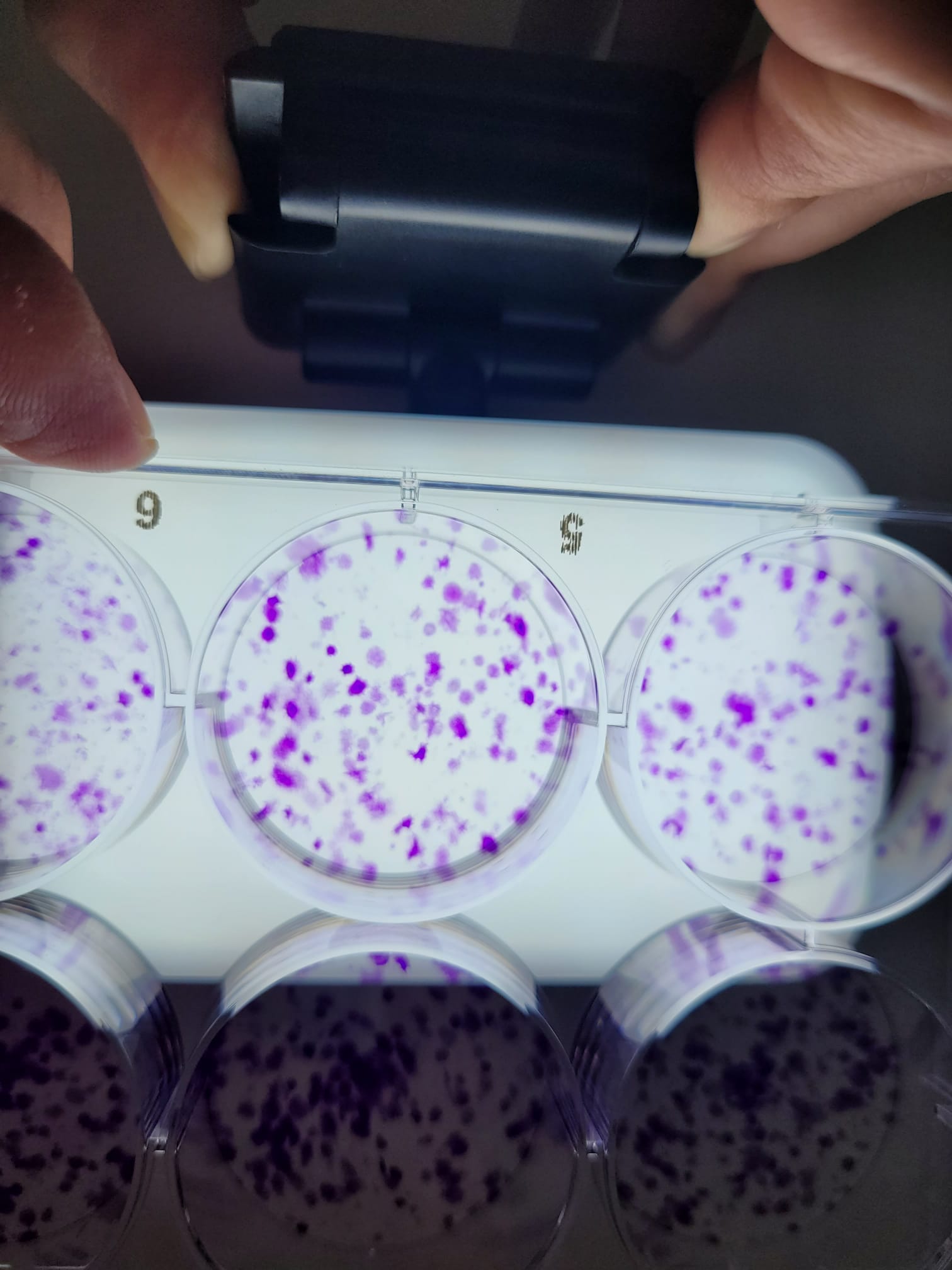

Supplement: Supplementary file 8 — Source data Fig. 6 [file 44319_2026_739_MOESM8_ESM.zip › Figure 6 Panel G/sh1_2.jpeg]

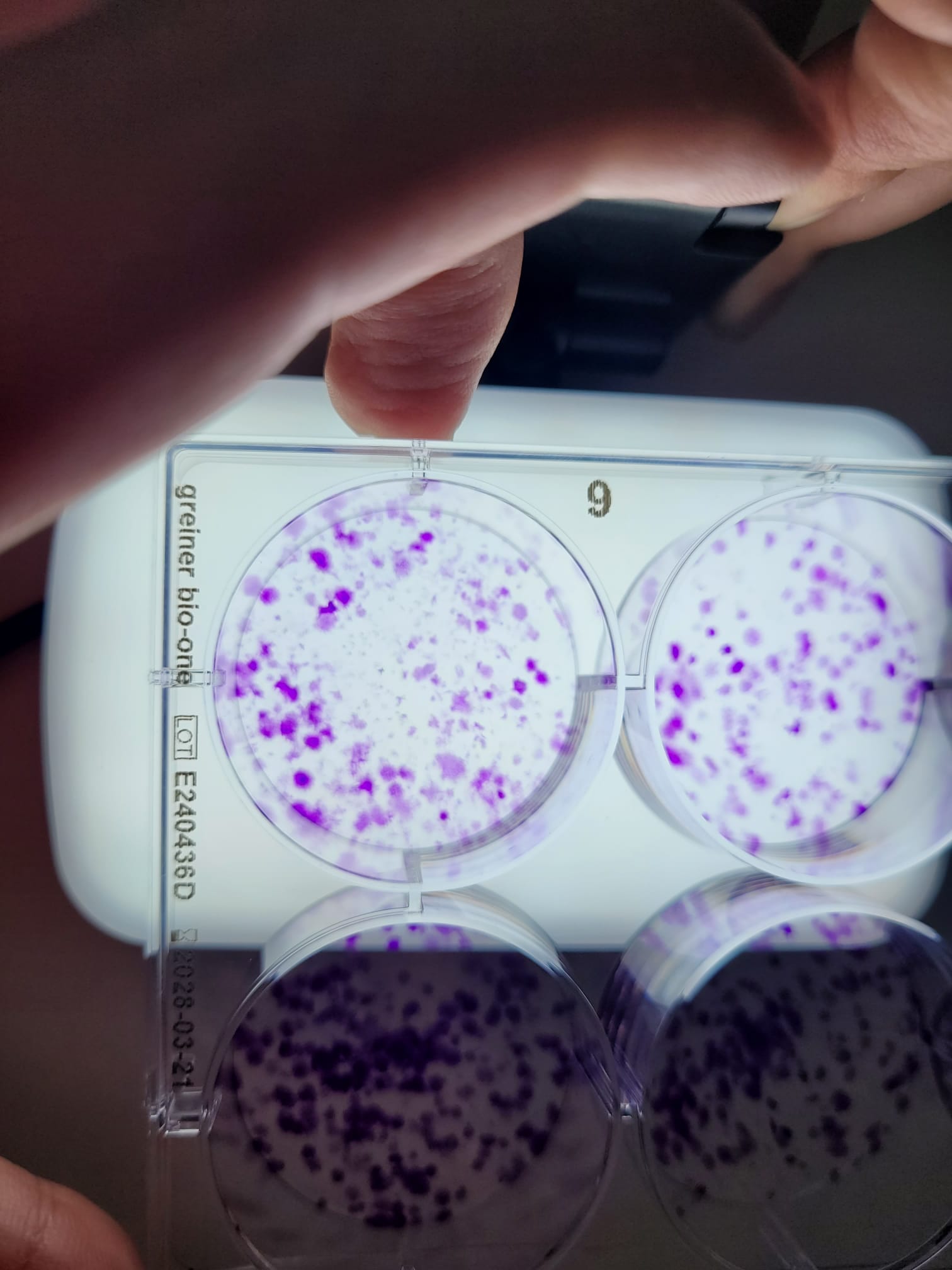

Supplement: Supplementary file 8 — Source data Fig. 6 [file 44319_2026_739_MOESM8_ESM.zip › Figure 6 Panel G/sh1_3.jpeg]

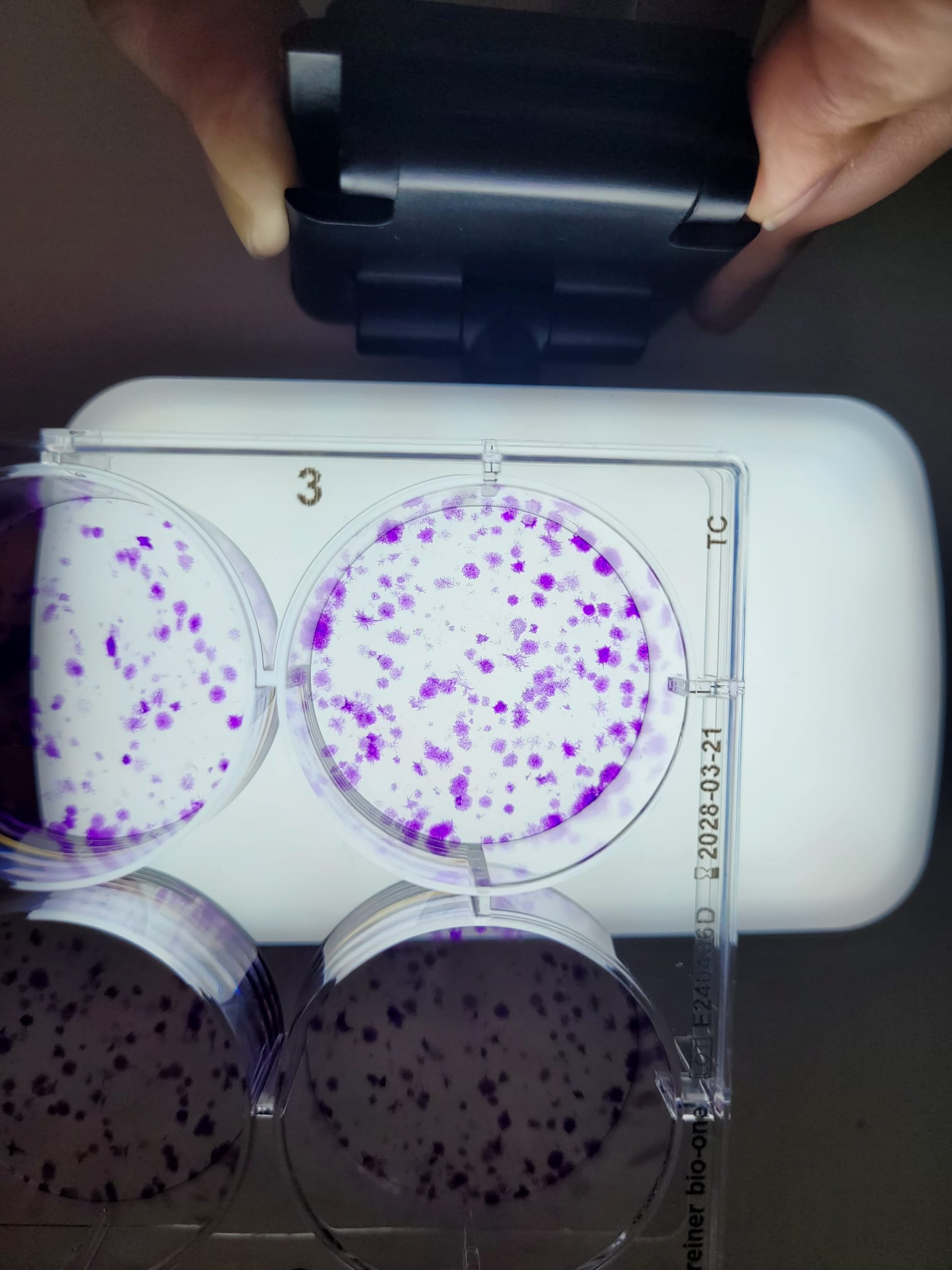

Supplement: Supplementary file 8 — Source data Fig. 6 [file 44319_2026_739_MOESM8_ESM.zip › Figure 6 Panel G/sh2_1.jpeg]

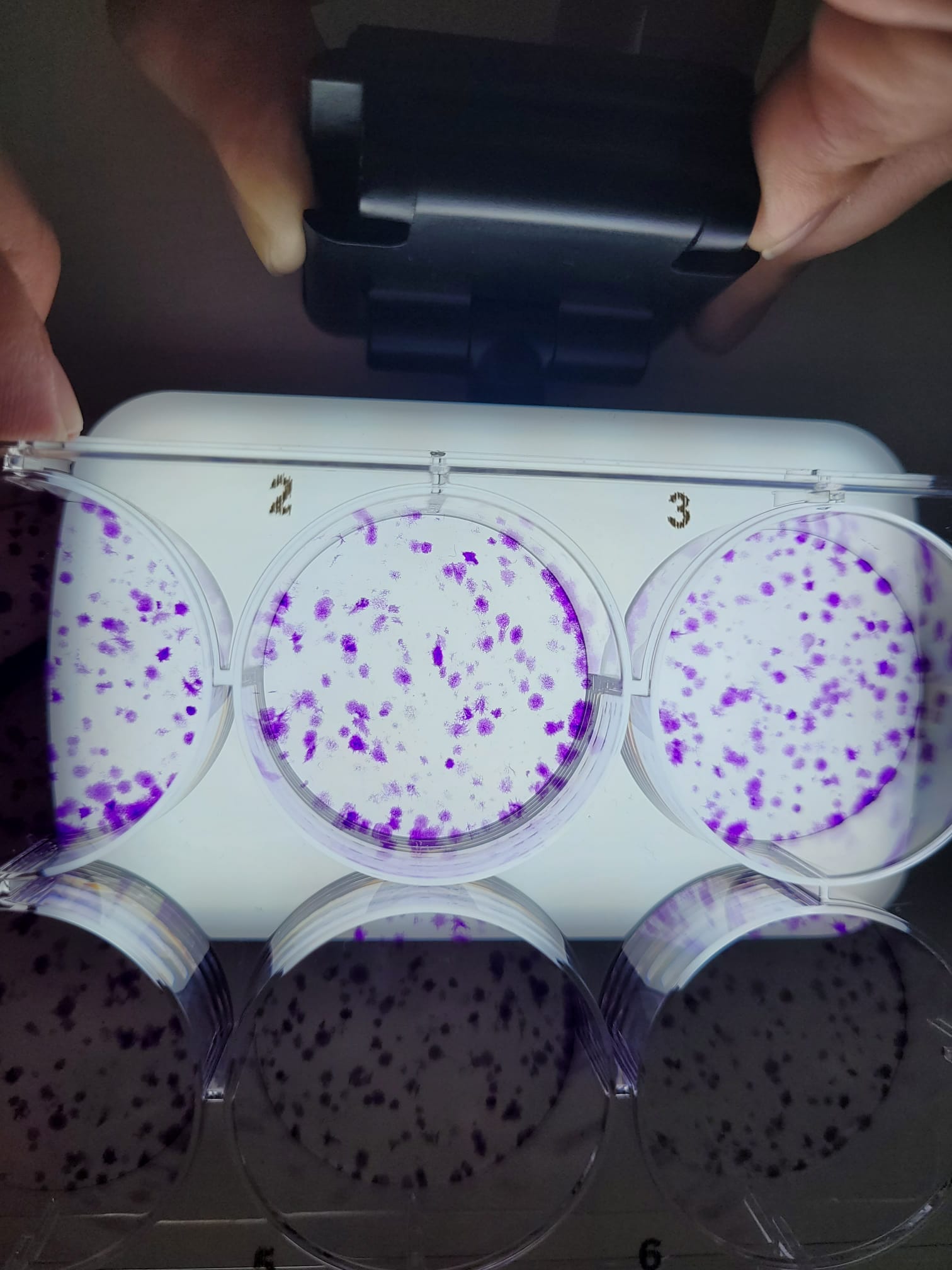

Supplement: Supplementary file 8 — Source data Fig. 6 [file 44319_2026_739_MOESM8_ESM.zip › Figure 6 Panel G/sh2_2.jpeg]

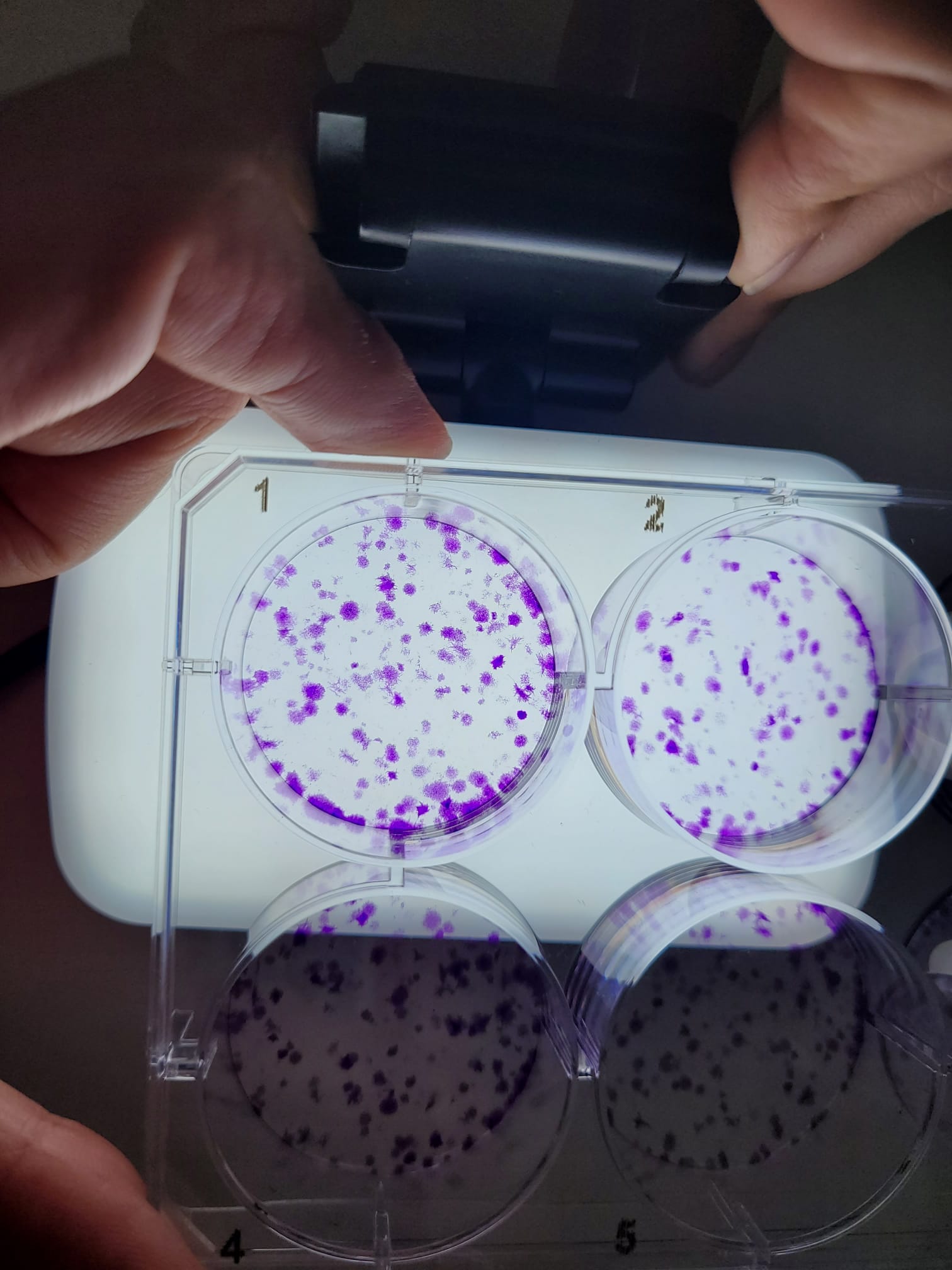

Supplement: Supplementary file 8 — Source data Fig. 6 [file 44319_2026_739_MOESM8_ESM.zip › Figure 6 Panel G/sh2_3.jpeg]

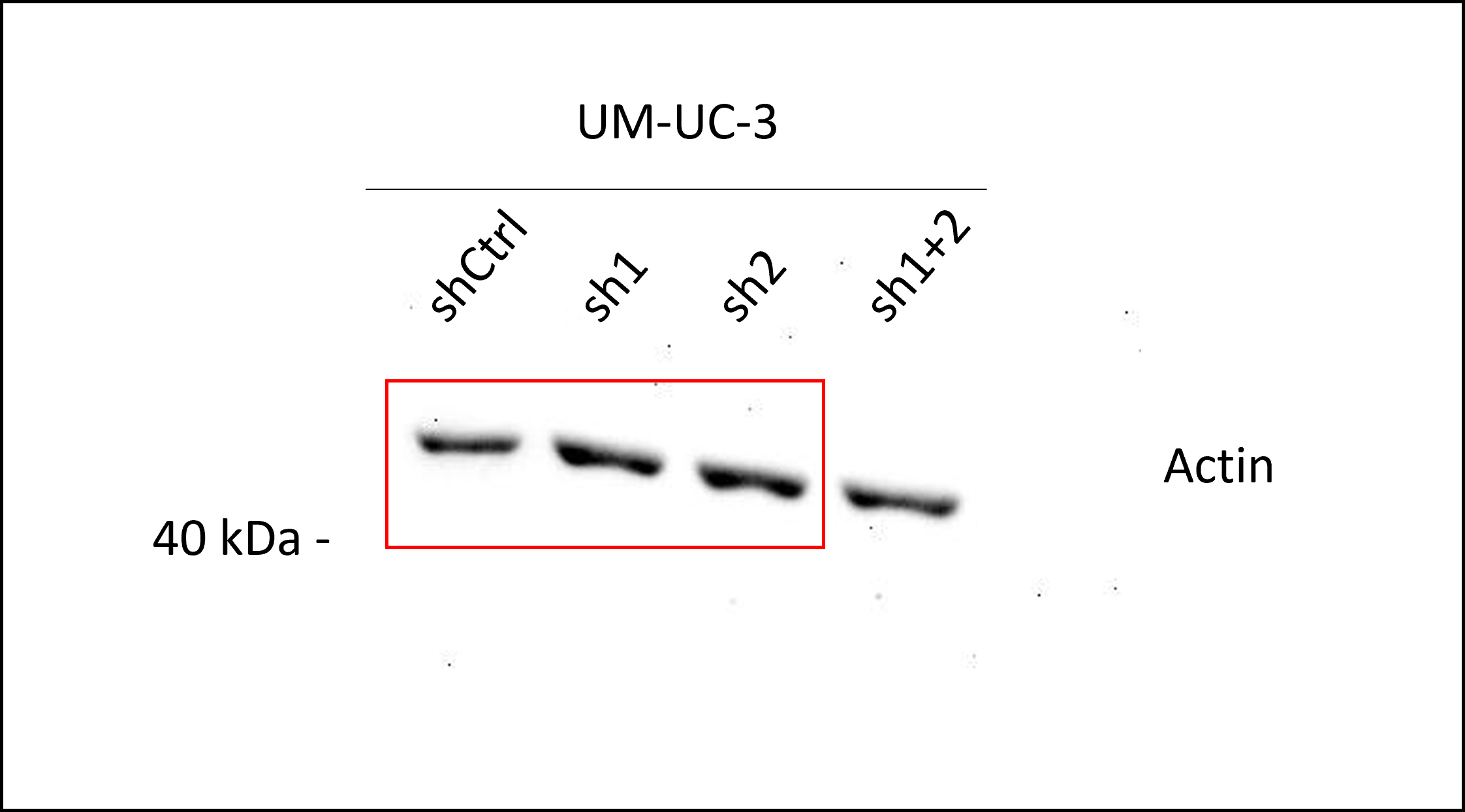

Supplement: Supplementary file 8 — Source data Fig. 6 [file 44319_2026_739_MOESM8_ESM.zip › Figure 6 Panel A/Western Actin UM-UC-3 annotated.png]

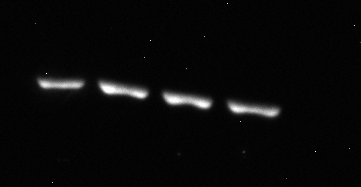

Supplement: Supplementary file 8 — Source data Fig. 6 [file 44319_2026_739_MOESM8_ESM.zip › Figure 6 Panel A/Western Actin UM-UC-3 Raw TIFF.tif]

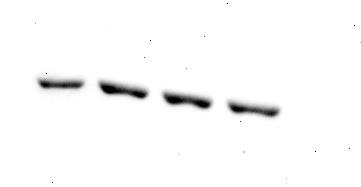

Supplement: Supplementary file 8 — Source data Fig. 6 [file 44319_2026_739_MOESM8_ESM.zip › Figure 6 Panel A/Western Actin UM-UC-3.jpg]

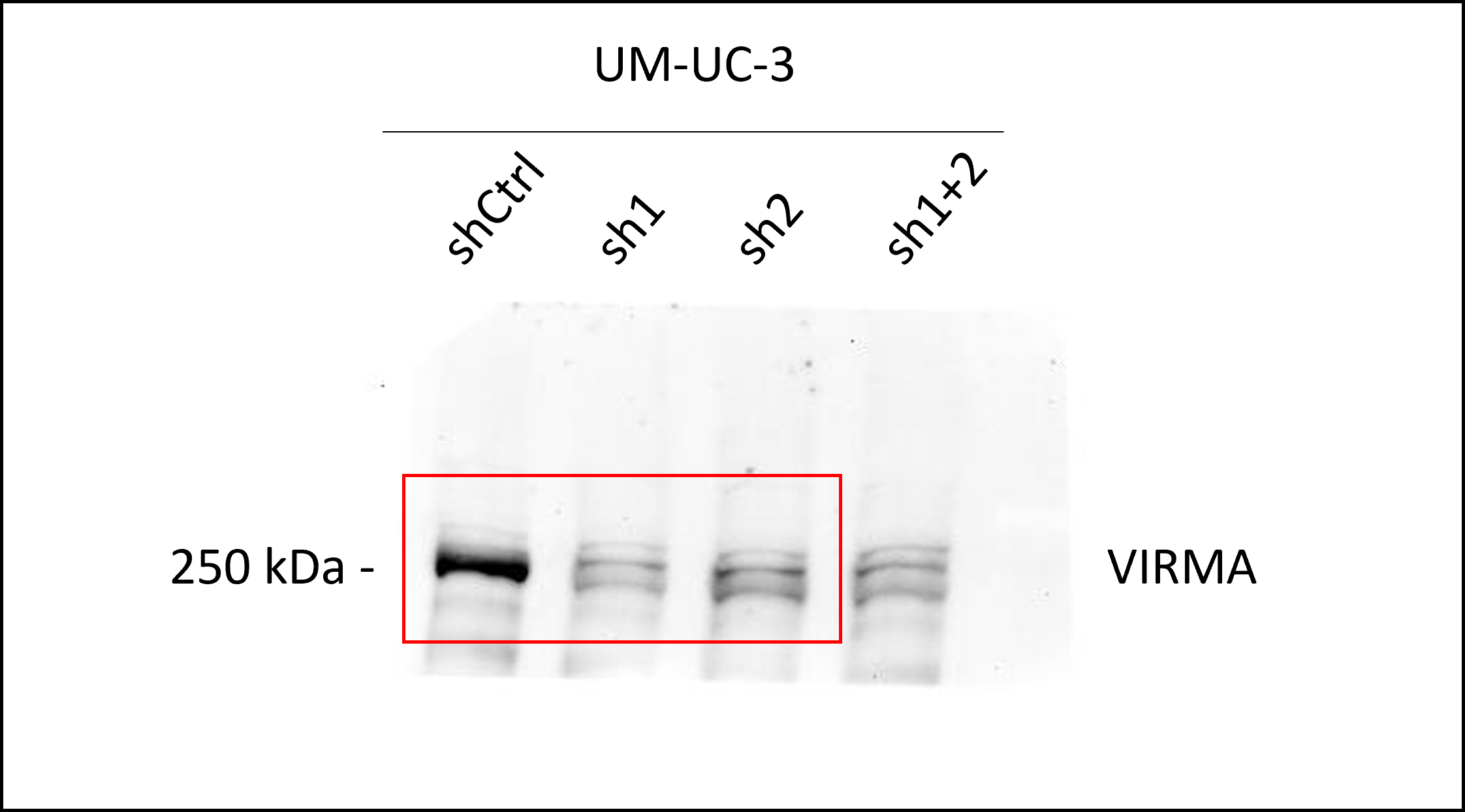

Supplement: Supplementary file 8 — Source data Fig. 6 [file 44319_2026_739_MOESM8_ESM.zip › Figure 6 Panel A/Western VIRMA UM-UC-3 annotated.png]

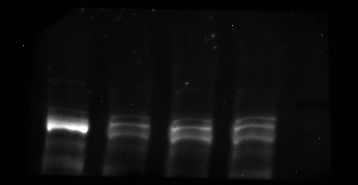

Supplement: Supplementary file 8 — Source data Fig. 6 [file 44319_2026_739_MOESM8_ESM.zip › Figure 6 Panel A/Western VIRMA UM-UC-3 Raw TIFF.tif]

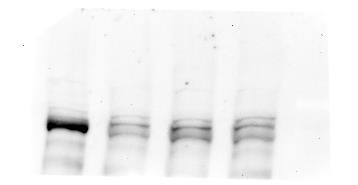

Supplement: Supplementary file 8 — Source data Fig. 6 [file 44319_2026_739_MOESM8_ESM.zip › Figure 6 Panel A/Western VIRMA UM-UC-3.jpg]

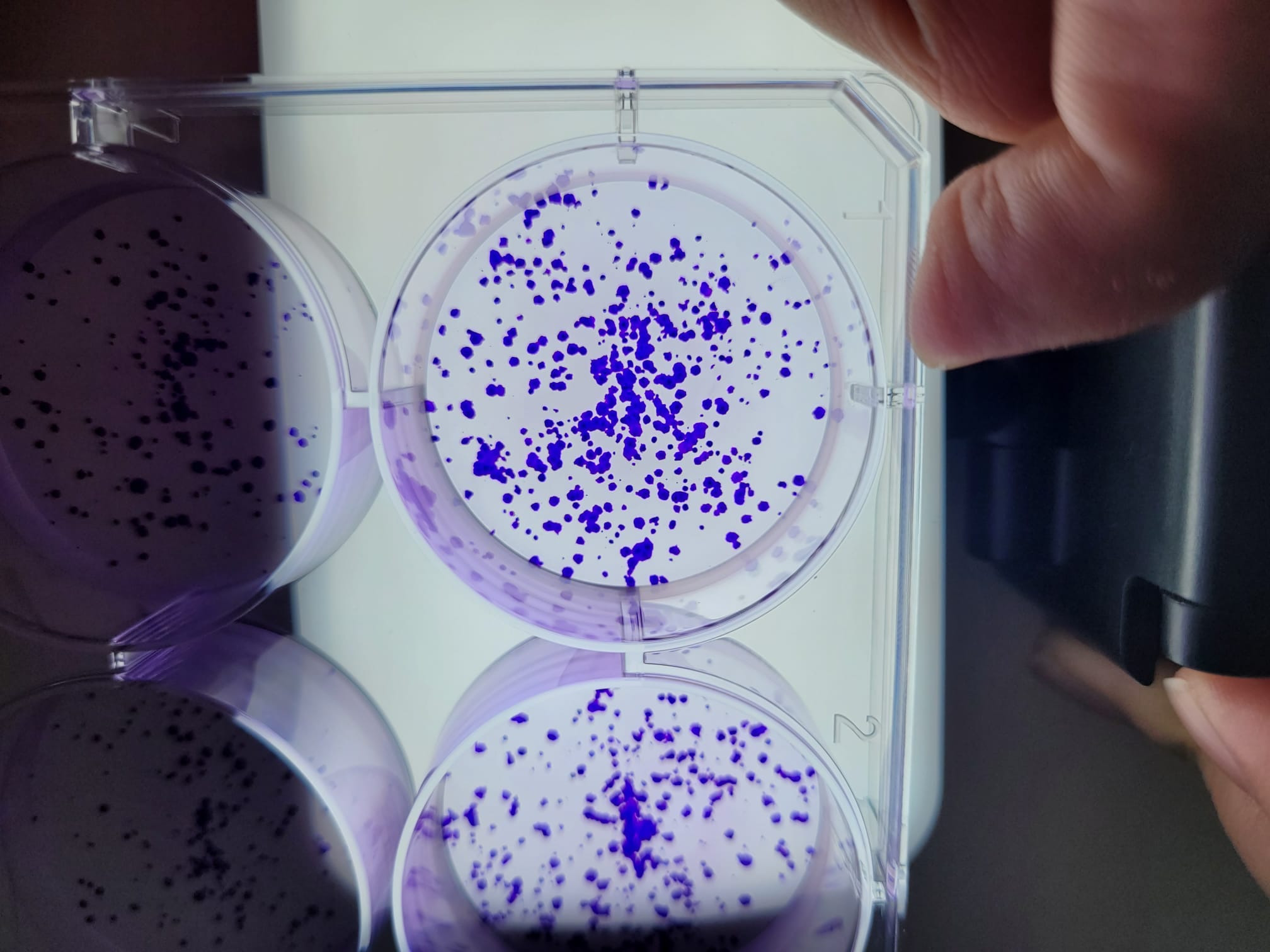

Supplement: Supplementary file 9 — Figure EV8 Source Data [file 44319_2026_739_MOESM9_ESM.zip › Figure EV8 Panel G/ctrl1.jpeg]

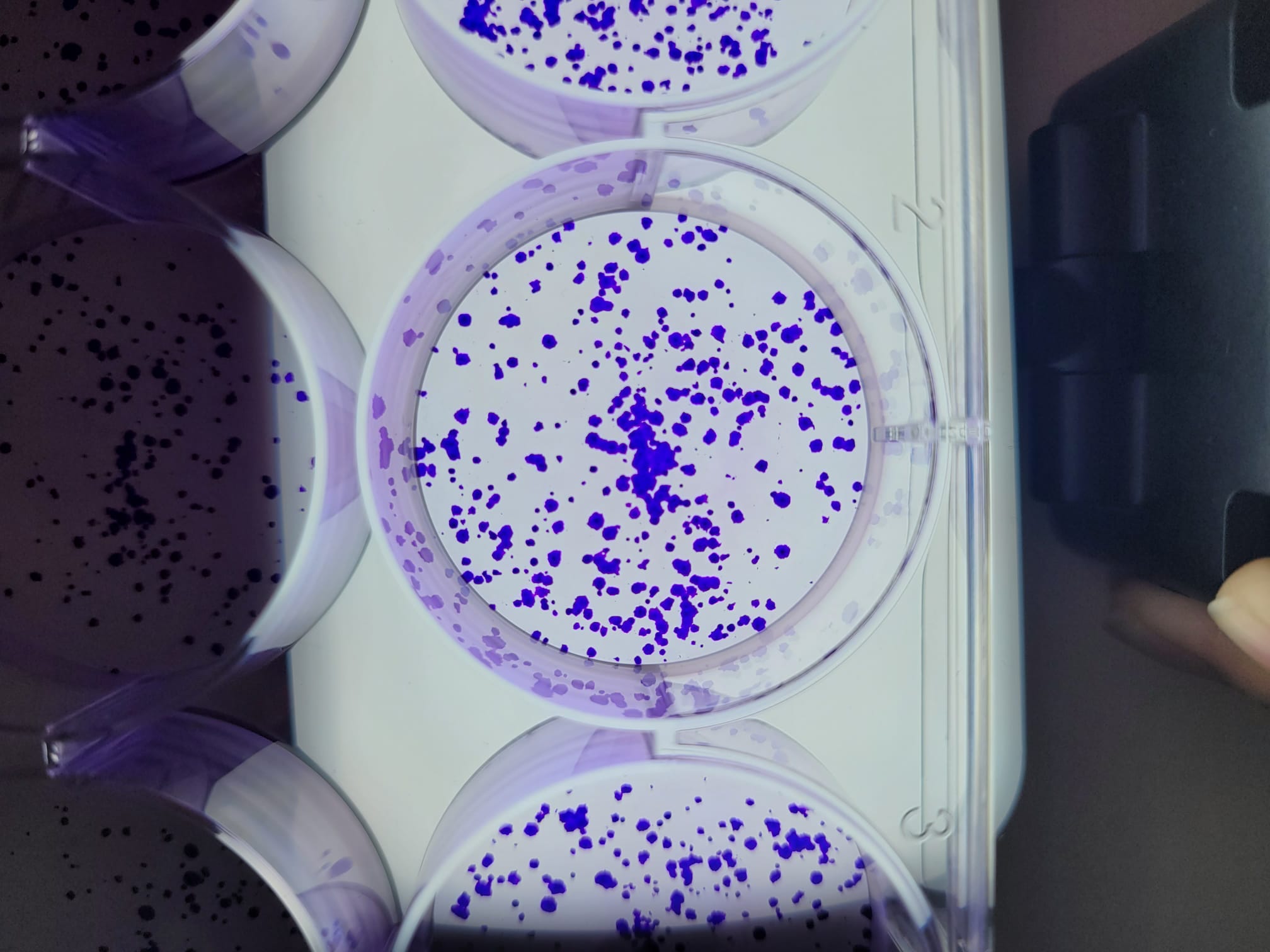

Supplement: Supplementary file 9 — Figure EV8 Source Data [file 44319_2026_739_MOESM9_ESM.zip › Figure EV8 Panel G/ctrl2.jpeg]

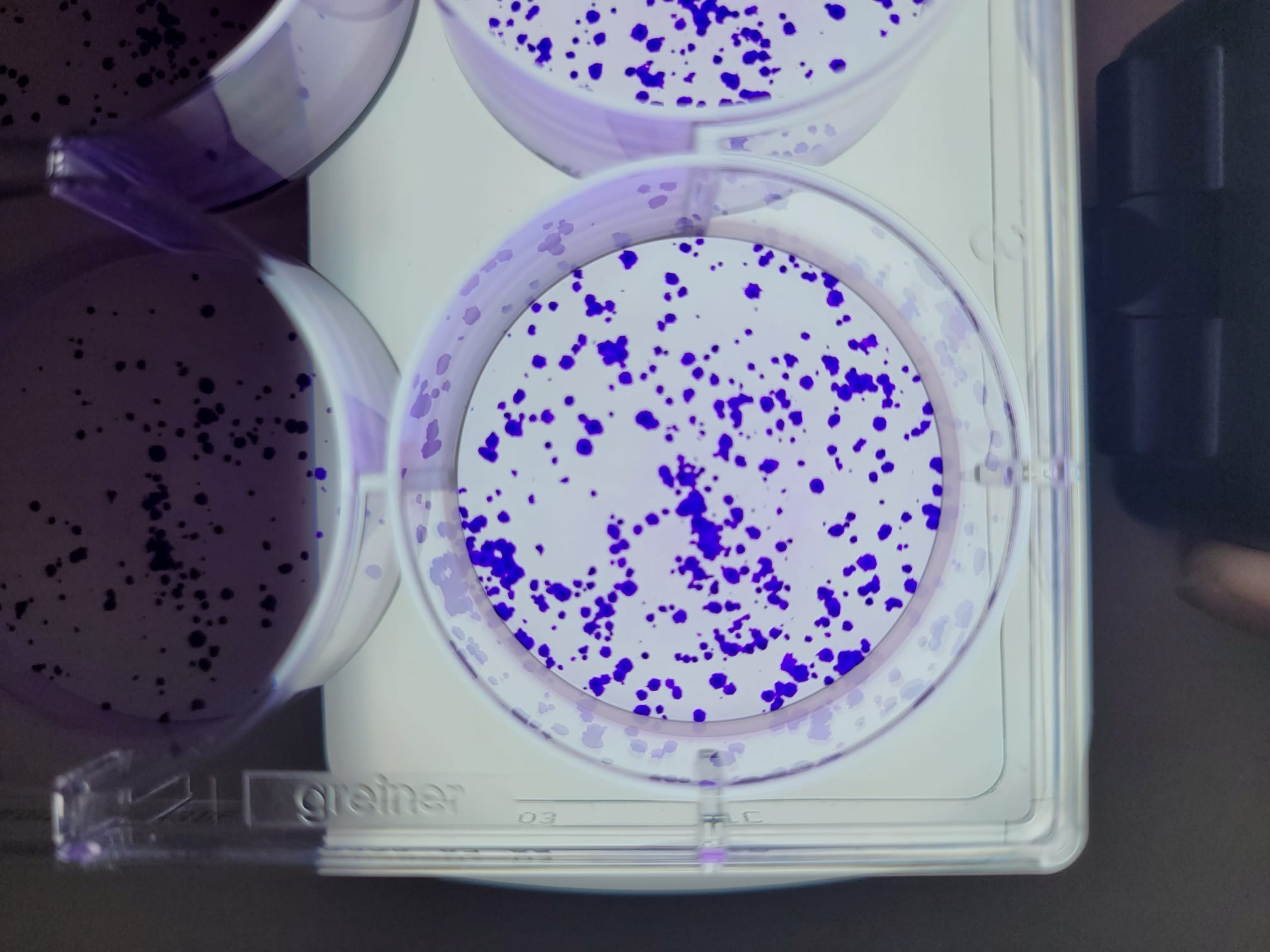

Supplement: Supplementary file 9 — Figure EV8 Source Data [file 44319_2026_739_MOESM9_ESM.zip › Figure EV8 Panel G/ctrl3.jpeg]

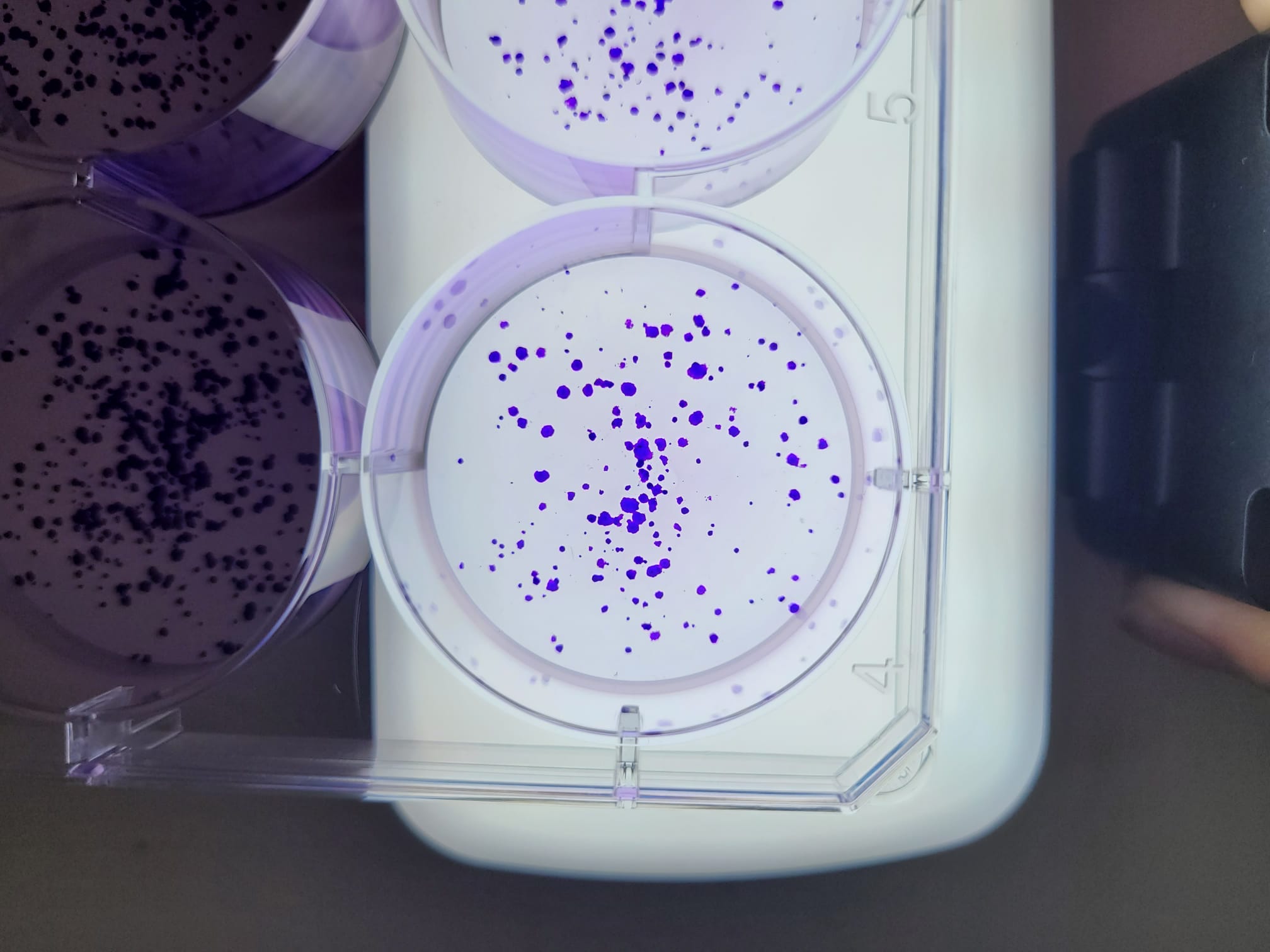

Supplement: Supplementary file 9 — Figure EV8 Source Data [file 44319_2026_739_MOESM9_ESM.zip › Figure EV8 Panel G/sh1_1.jpeg]

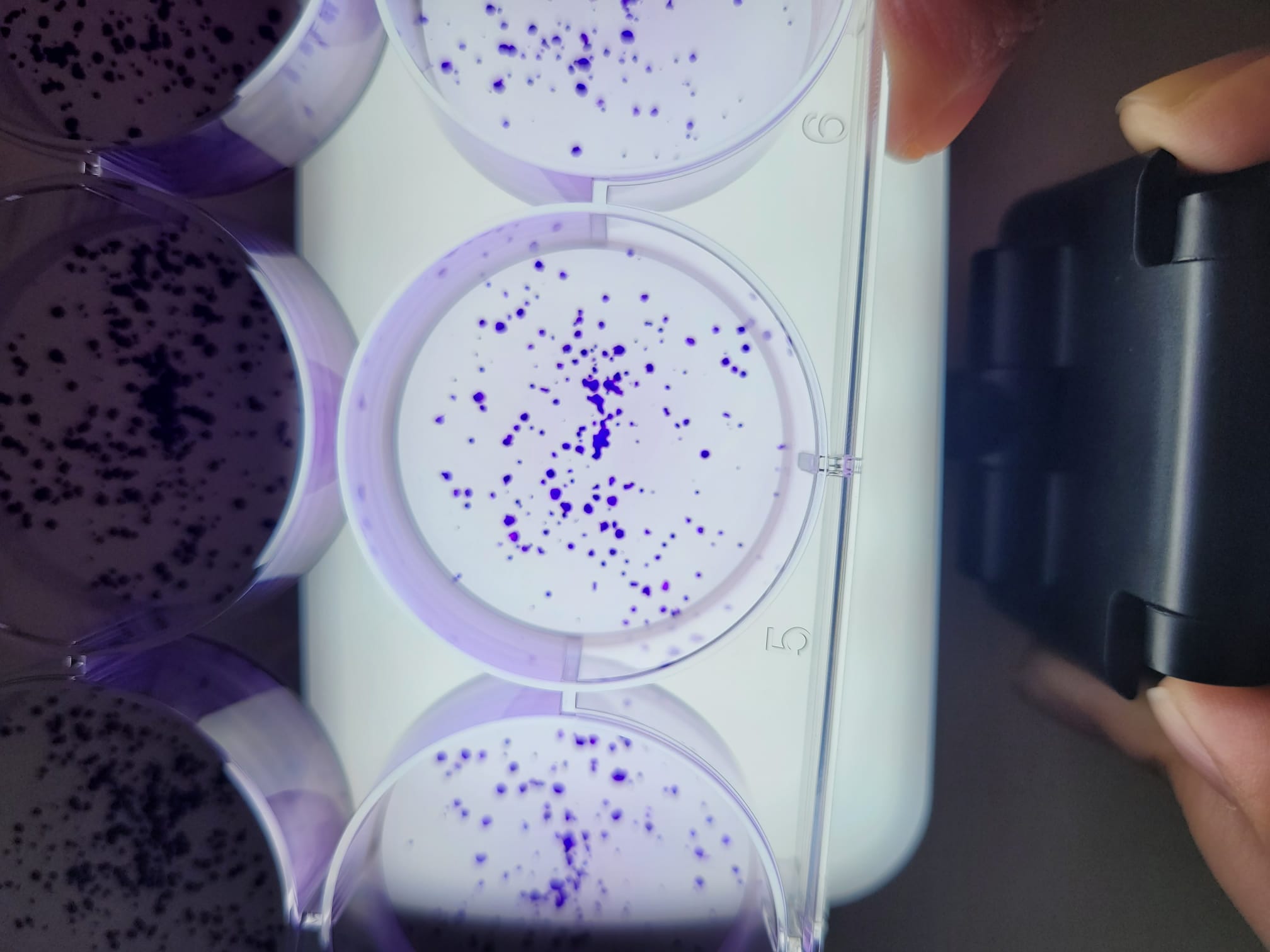

Supplement: Supplementary file 9 — Figure EV8 Source Data [file 44319_2026_739_MOESM9_ESM.zip › Figure EV8 Panel G/sh1_2.jpeg]

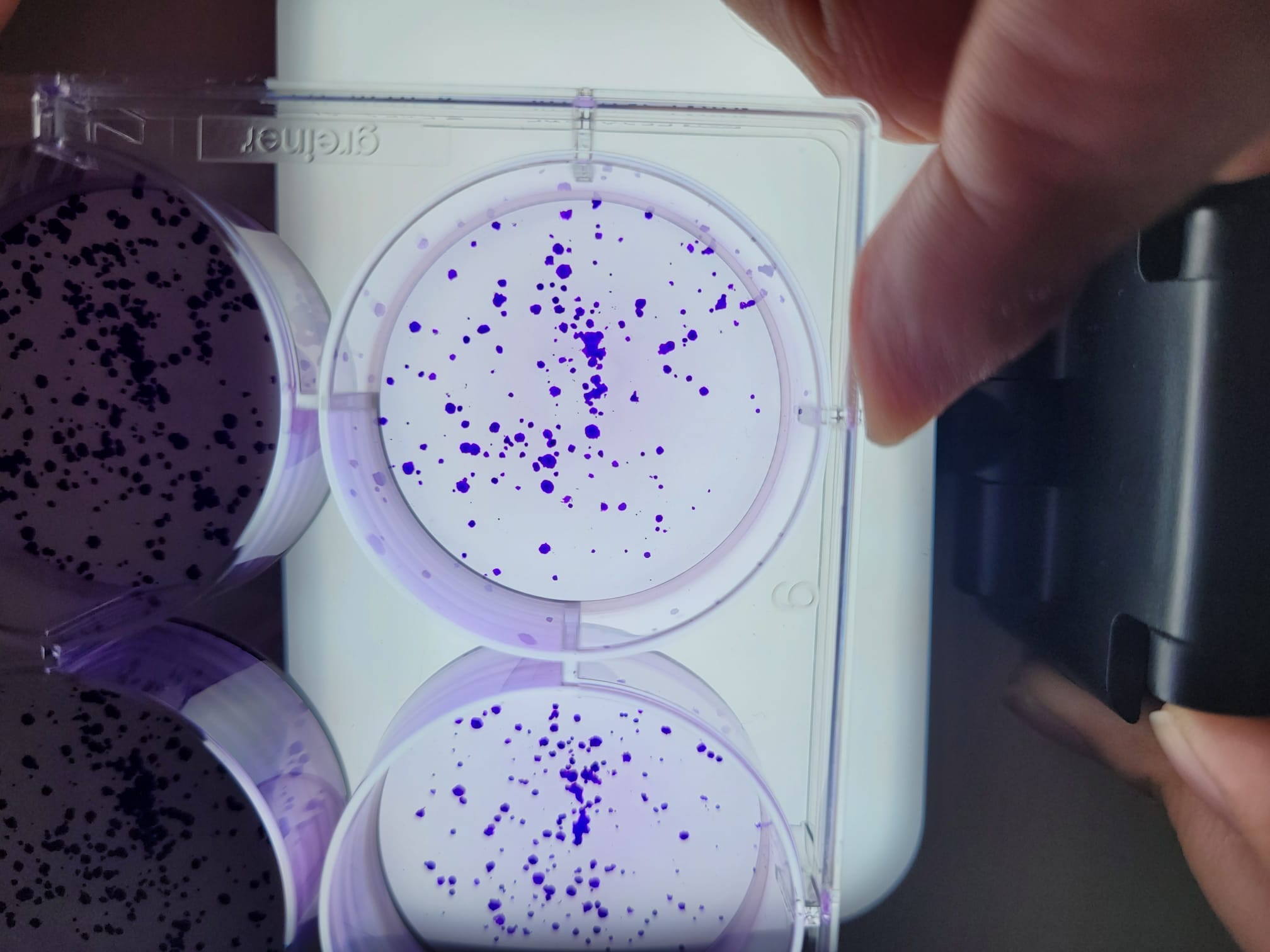

Supplement: Supplementary file 9 — Figure EV8 Source Data [file 44319_2026_739_MOESM9_ESM.zip › Figure EV8 Panel G/sh1_3.jpeg]

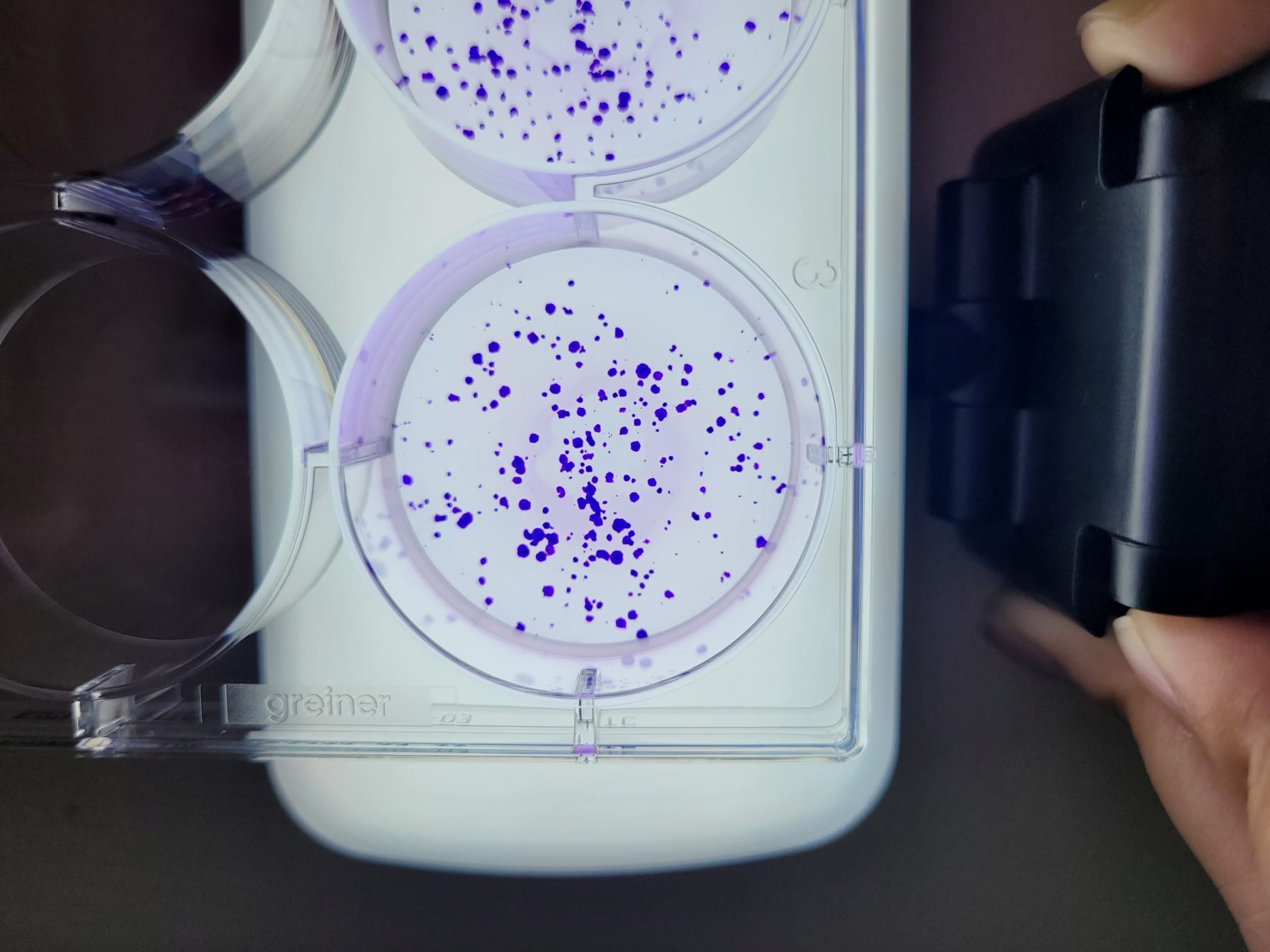

Supplement: Supplementary file 9 — Figure EV8 Source Data [file 44319_2026_739_MOESM9_ESM.zip › Figure EV8 Panel G/sh2_1.jpeg]

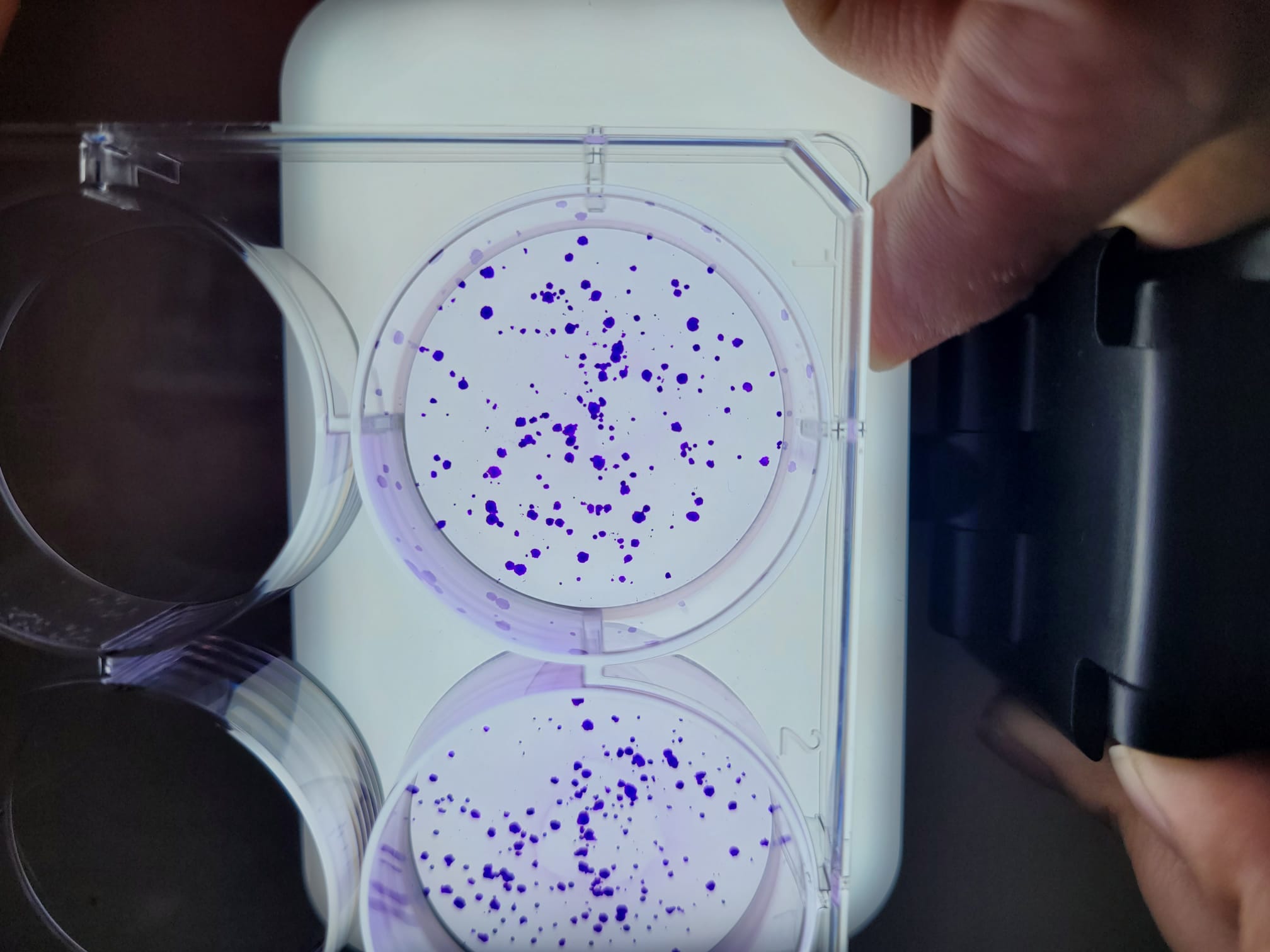

Supplement: Supplementary file 9 — Figure EV8 Source Data [file 44319_2026_739_MOESM9_ESM.zip › Figure EV8 Panel G/sh2_2.jpeg]

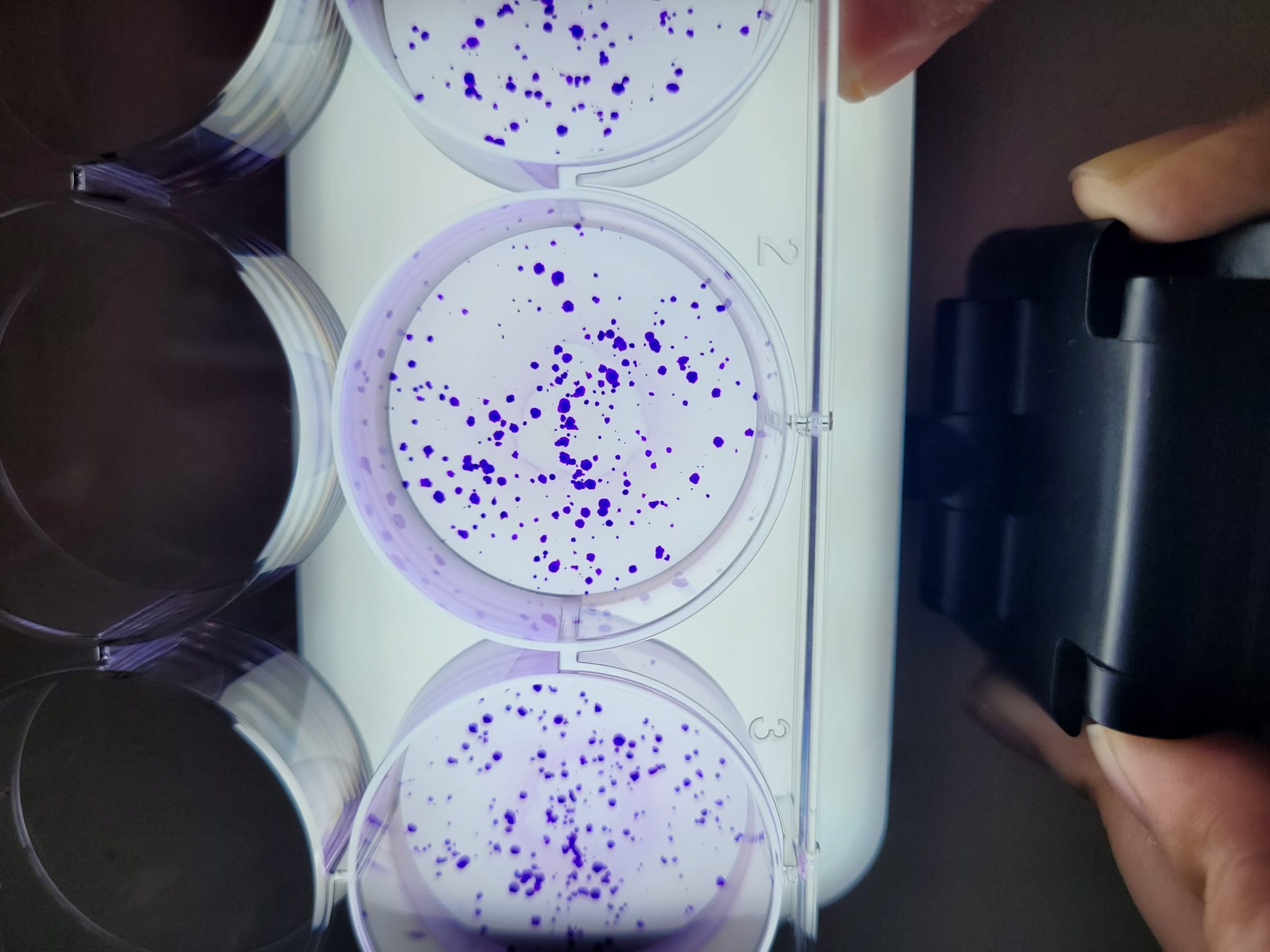

Supplement: Supplementary file 9 — Figure EV8 Source Data [file 44319_2026_739_MOESM9_ESM.zip › Figure EV8 Panel G/sh2_3.jpeg]

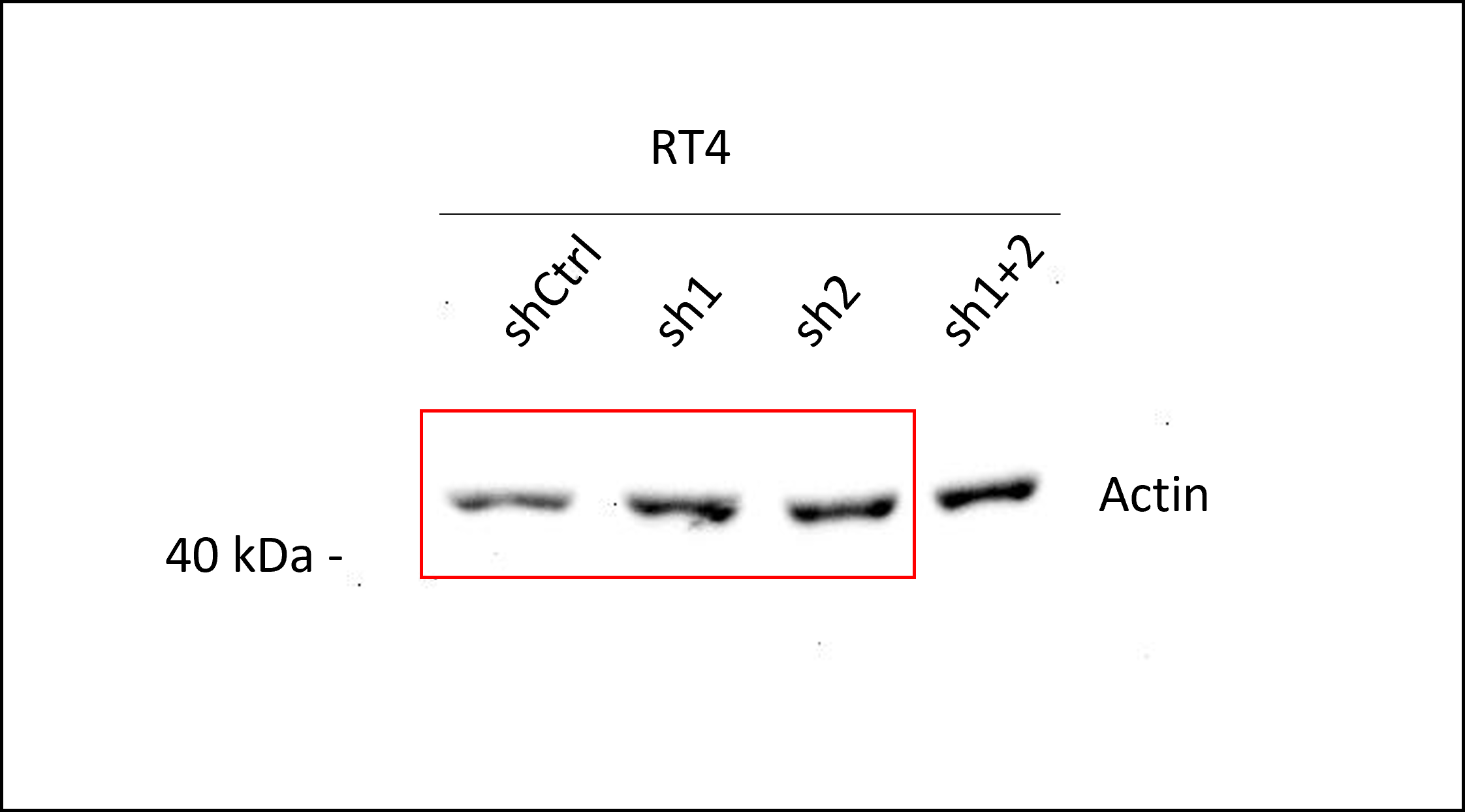

Supplement: Supplementary file 9 — Figure EV8 Source Data [file 44319_2026_739_MOESM9_ESM.zip › Figure EV8 Panel A/Western Actin RT4 annotated.png]

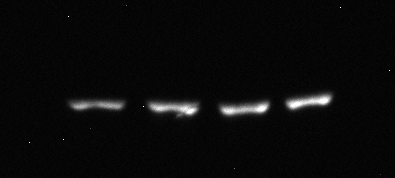

Supplement: Supplementary file 9 — Figure EV8 Source Data [file 44319_2026_739_MOESM9_ESM.zip › Figure EV8 Panel A/Western Actin RT4 Raw TIFF.tif]

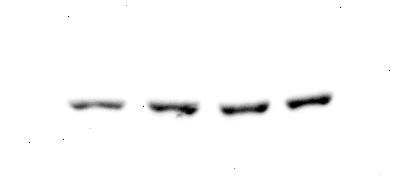

Supplement: Supplementary file 9 — Figure EV8 Source Data [file 44319_2026_739_MOESM9_ESM.zip › Figure EV8 Panel A/Western Actin RT4.jpg]

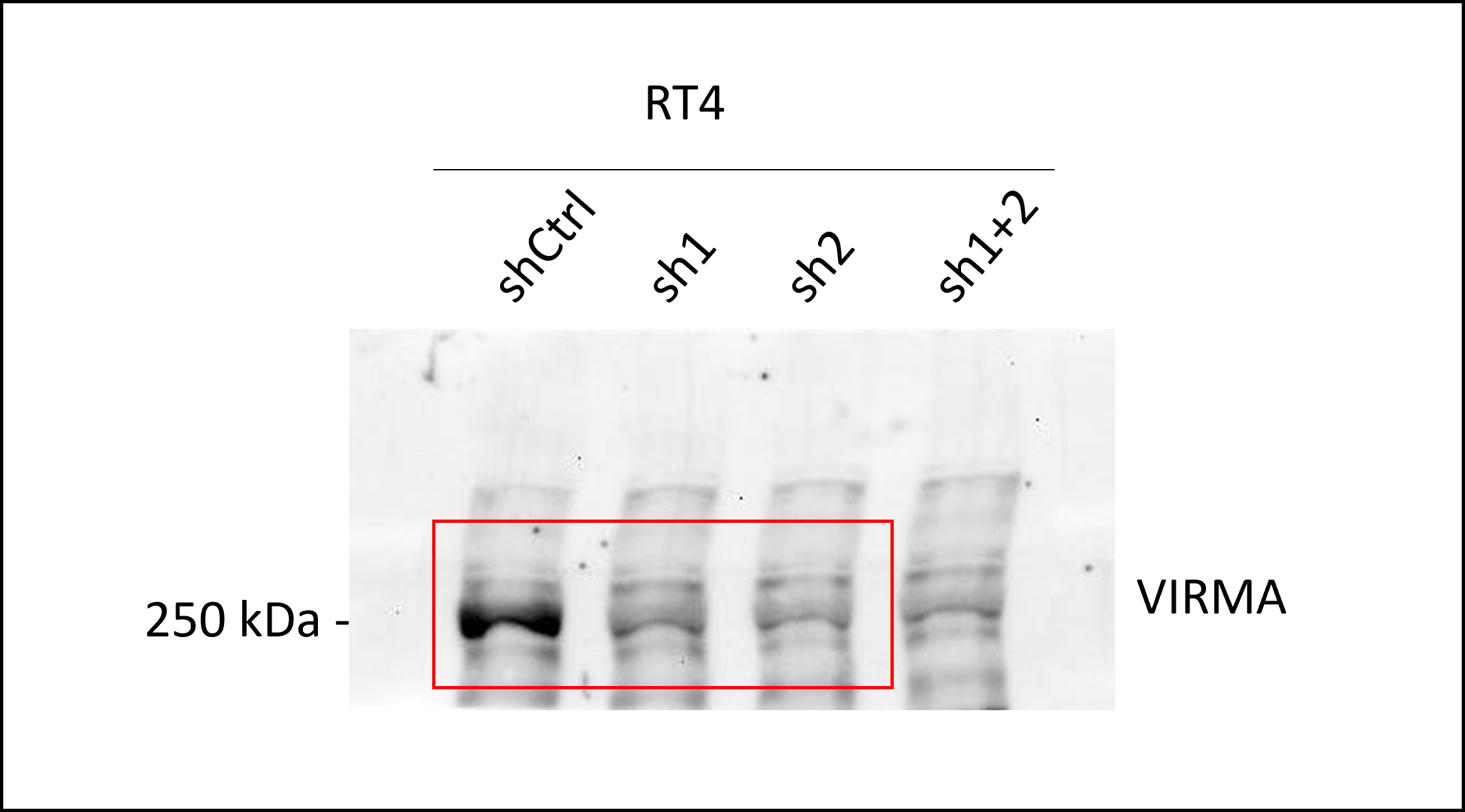

Supplement: Supplementary file 9 — Figure EV8 Source Data [file 44319_2026_739_MOESM9_ESM.zip › Figure EV8 Panel A/Western VIRMA RT4 annotated.png]

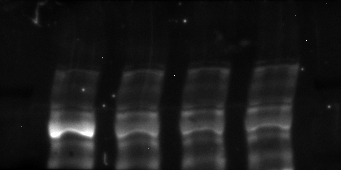

Supplement: Supplementary file 9 — Figure EV8 Source Data [file 44319_2026_739_MOESM9_ESM.zip › Figure EV8 Panel A/Western VIRMA RT4 Raw TIFF.tif]

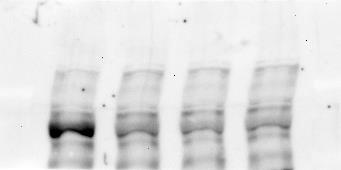

Supplement: Supplementary file 9 — Figure EV8 Source Data [file 44319_2026_739_MOESM9_ESM.zip › Figure EV8 Panel A/Western VIRMA RT4.jpg]
