# Supplementary material for: The bladder cancer m6A landscape is defined by global methylation dilution and focal 3′-UTR hypermethylation
Source: EMBO Rep. 2026 Mar 23;27(8):2118–43. doi: 10.1038/s44319-026-00739-y (PMC13121636; doi:10.1038/s44319-026-00739-y)
Supplement: Supplementary file 10 — Expanded View Figures [file 44319_2026_739_MOESM10_ESM.pdf]

Expanded View Figures

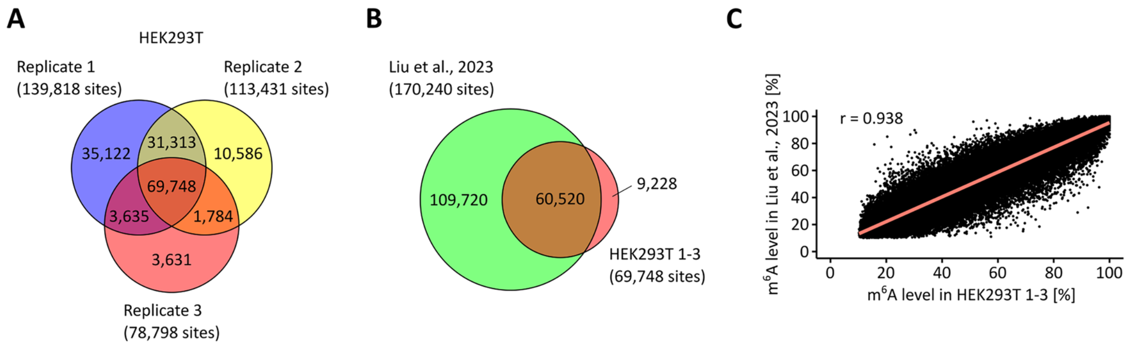

Figure EV1. GLORI allows for the reproducible detection and quantification of m⁶A sites in HEK293T cells.

(**A**) In the intersection of the three HEK293T cell replicates, roughly 70,000 m^6A sites were detected by GLORI. (**B**) The majority (87%) of those 70,000 m^6A sites were also reported by the original study. (**C**) The mean m^6A methylation levels from the 60,000 m^6A sites detected in both studies were comparable.

 © The Author(s)

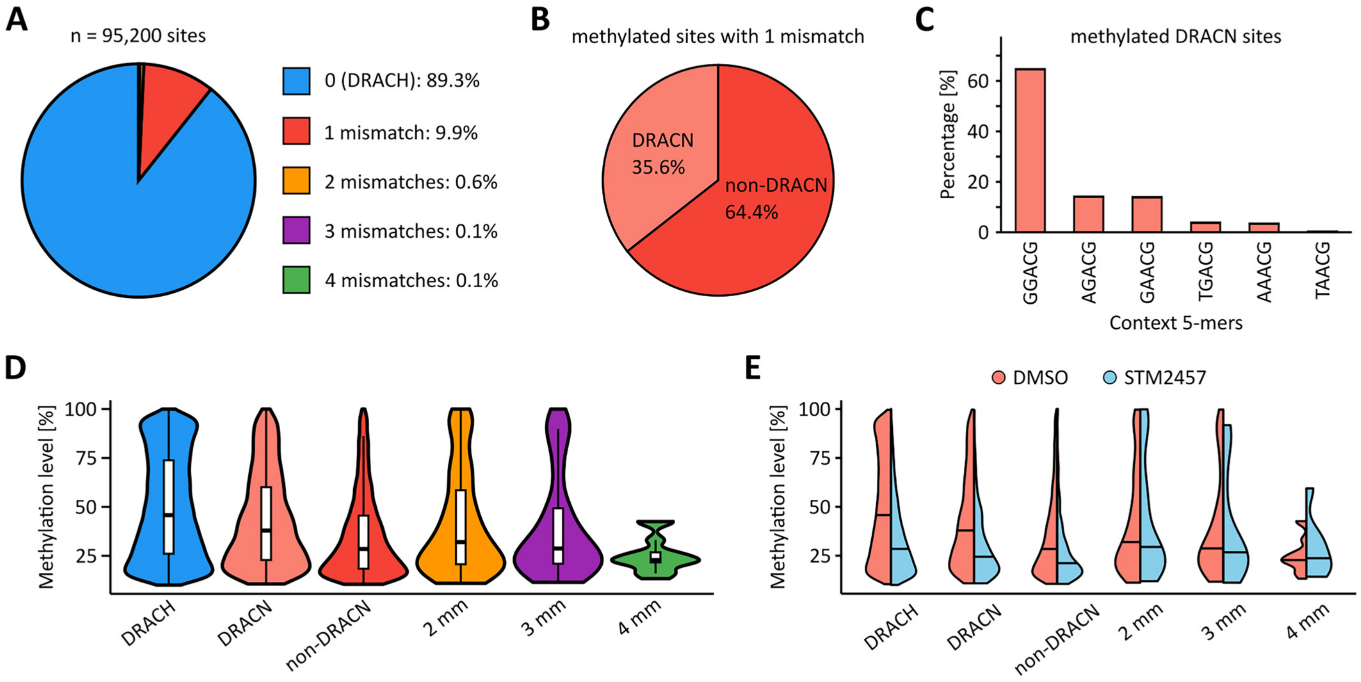

Figure EV2. m^{6}A sites detected in DRAC(H) motifs are the most robust.

(**A**) m^{6}A sites detected in different sequence motifs. Numbers describe mismatches compared to the canonical DRAC(H) motif. (**B**) Distribution of m^{6}A sites detected in 5-mer motifs with one mismatch compared to DRAC(H). In DRACN motifs, the fifth base of the 5-mer is variable, while the first four bases are DRAC(H)-conform. In non-DRACN motifs, the mismatch occurs in one of the first four bases. (**C**) Frequency of DRACN motifs detected in T24 cells. (**D**) Methylation level distribution of m^{6}A sites detected in the different motif categories. $n = 95{,}200$ m^{6}A sites shared among the three T24 replicates were used for sequence motif analyses. Boxplots were generated in R using ggplot2. The centre line indicates the median (50th percentile). The box bounds represent the first and third quartiles (25th and 75th percentiles), with box height equal to the IQR. Whiskers extend to the most extreme values within 1.5 * IQR from the quartiles. (**E**) Methylation level distribution of m^{6}A sites detected in the different motif categories in T24 cells treated with DMSO or the METTL3 inhibitor STM2457. mm = mismatch compared to DRAC(H). $n = 95{,}200$ m^{6}A sites shared among the three T24 DMSO replicates and $n = 17{,}189$ m^{6}A sites shared among the three T24 STM2457 replicates were used for this sequence motif analysis.

© The Author(s)

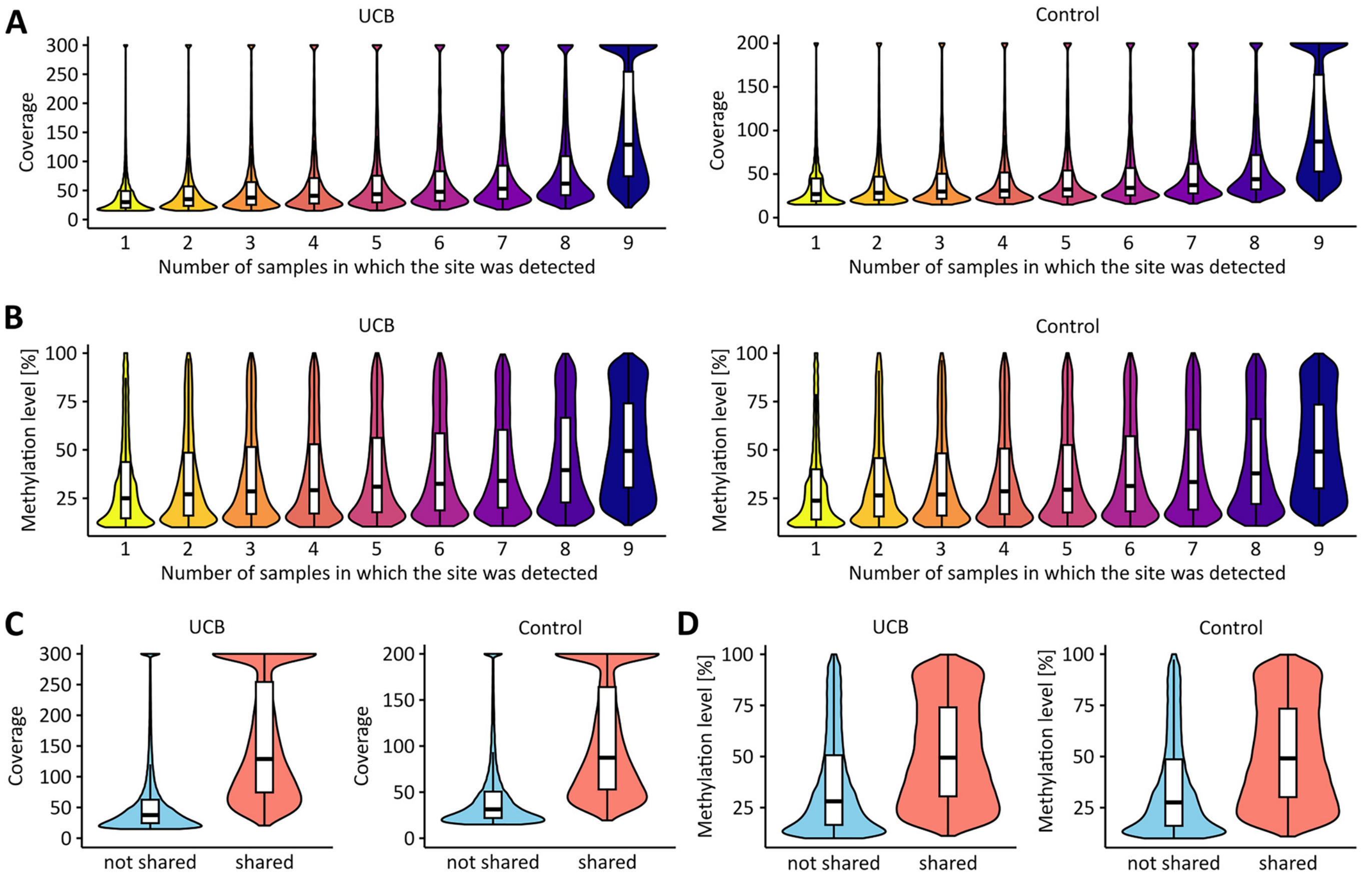

Figure EV3. Shared m⁶A sites are characterized by high coverage and high methylation levels.

(**A**) m⁶A sites were grouped based on the number of samples in which they were detected (from 1 to 9), and the distributions of sequencing coverage are shown for each detection category. (**B**) m⁶A sites were grouped based on the number of samples in which they were detected (from 1 to 9), and the distributions of methylation levels are shown for each detection category. (**C**) m⁶A sites detected in all nine samples (shared) were compared against those detected in only 1–8 samples (not shared), and the distributions of sequencing coverage are shown. (**D**) m⁶A sites detected in all nine samples (shared) were compared against those detected in only 1–8 samples (not shared) and the distributions of methylation levels are shown. $n = 242{,}371$ sites detected in nine UCB tissue samples and $n = 191{,}722$ sites detected in nine control tissue samples were used for these analyses. Boxplots were generated in R using ggplot2. The centre line indicates the median (50th percentile). The box bounds represent the first and third quartiles (25th and 75th percentiles), with box height equal to the IQR. Whiskers extend to the most extreme values within 1.5 * IQR from the quartiles.

 © The Author(s)

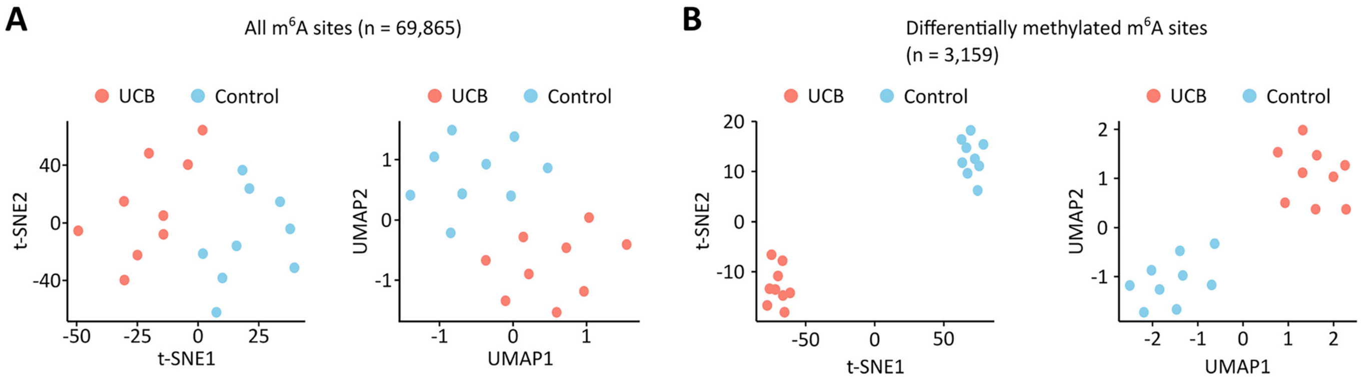

Figure EV4. tSNE and UMAP analyses separating control and UCB tissue samples based on their m^{6}A signatures.

(**A**) Dimensionality reduction analyses considering all m^{6}A sites. (**B**) Dimensionality reduction analyses considering differentially methylated m^{6}A sites. These analyses were performed based on $n = 9$ biological replicates.

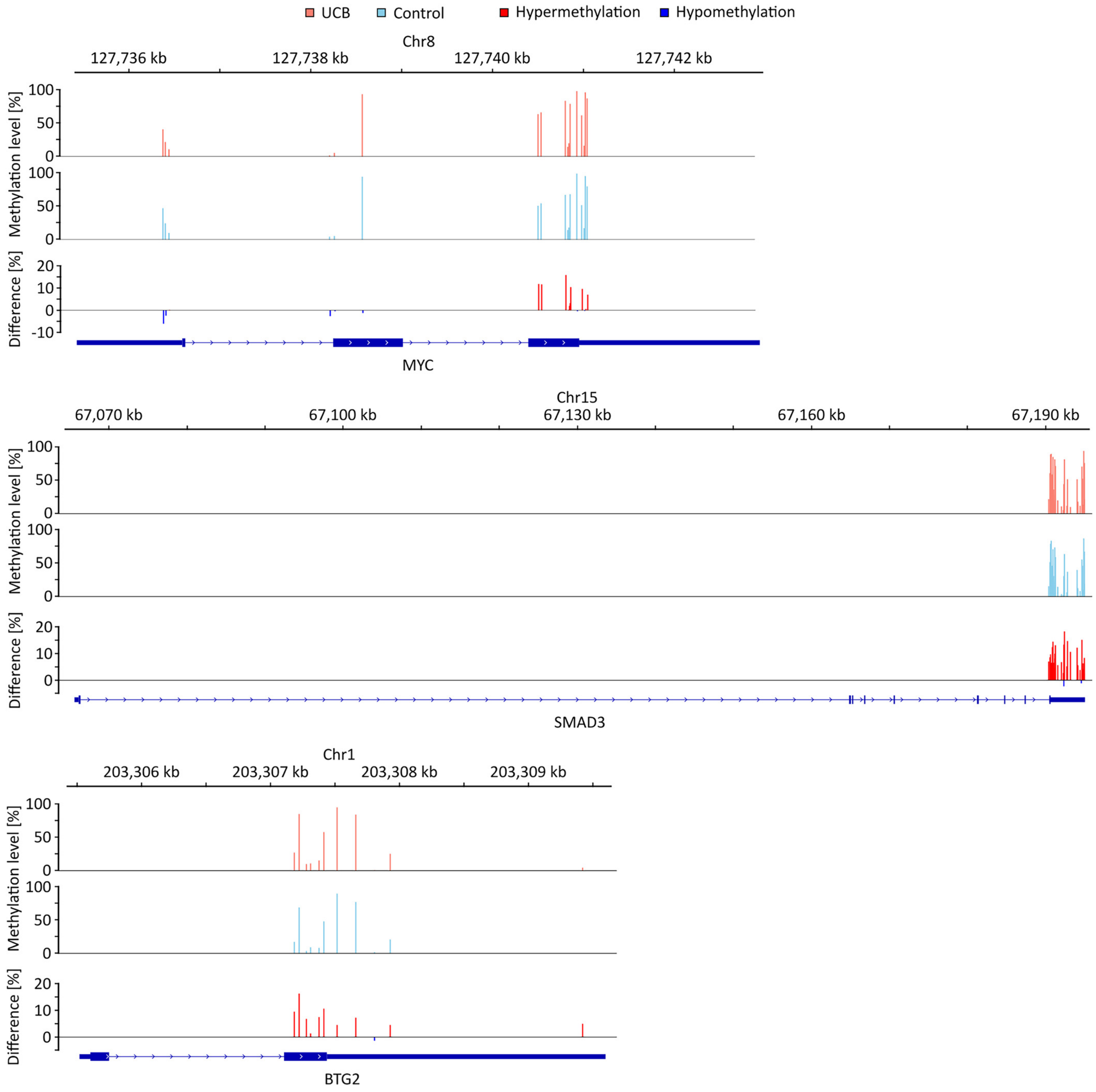

Figure EV5. Methylation level changes in selected transcripts.

IGV browser tracks of MYC, SMAD3, and BTG2 transcripts showing altered methylation levels in m^{6}A sites comparing control and UCB tissues. These analyses were performed based on $n = 9$ biological replicates.

 © The Author(s)

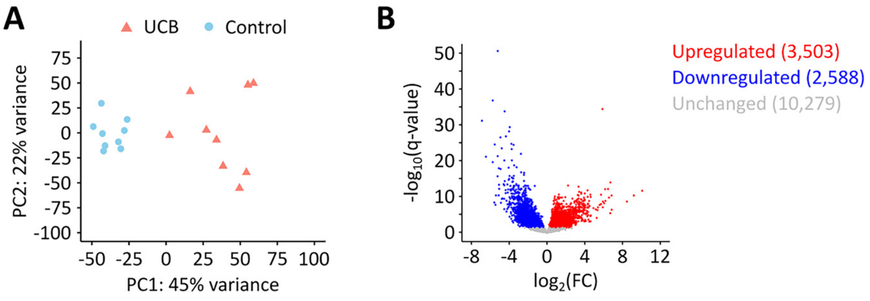

Figure EV6. RNA sequencing analysis of clinical samples.

(**A**) PCA demonstrating that patient samples can be separated based on their gene expression profile. (**B**) Differential gene expression analysis showing global deregulation of genes in UCB. q-value < 0.05. These analyses were performed based on $n = 9$ biological replicates.

© The Author(s)

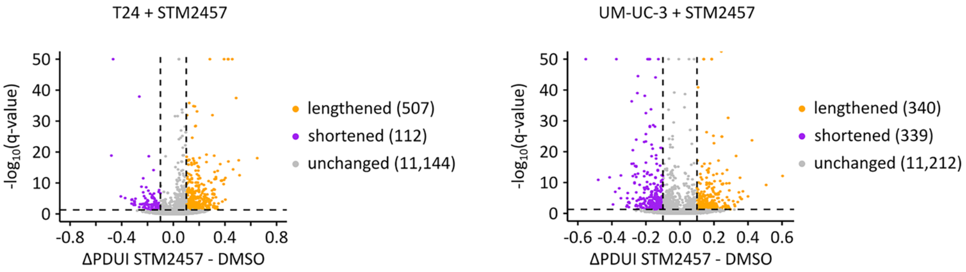

Figure EV7. Detection of alternative polyadenylation events in two UCB cell lines treated with the METTL3 inhibitor STM2457.

In T24 cells, most transcripts were found to be lengthened, while no clear tendency was observed in UM-UC-3 cells. These analyses were performed based on $n = 3$ biological replicates.

 © The Author(s)

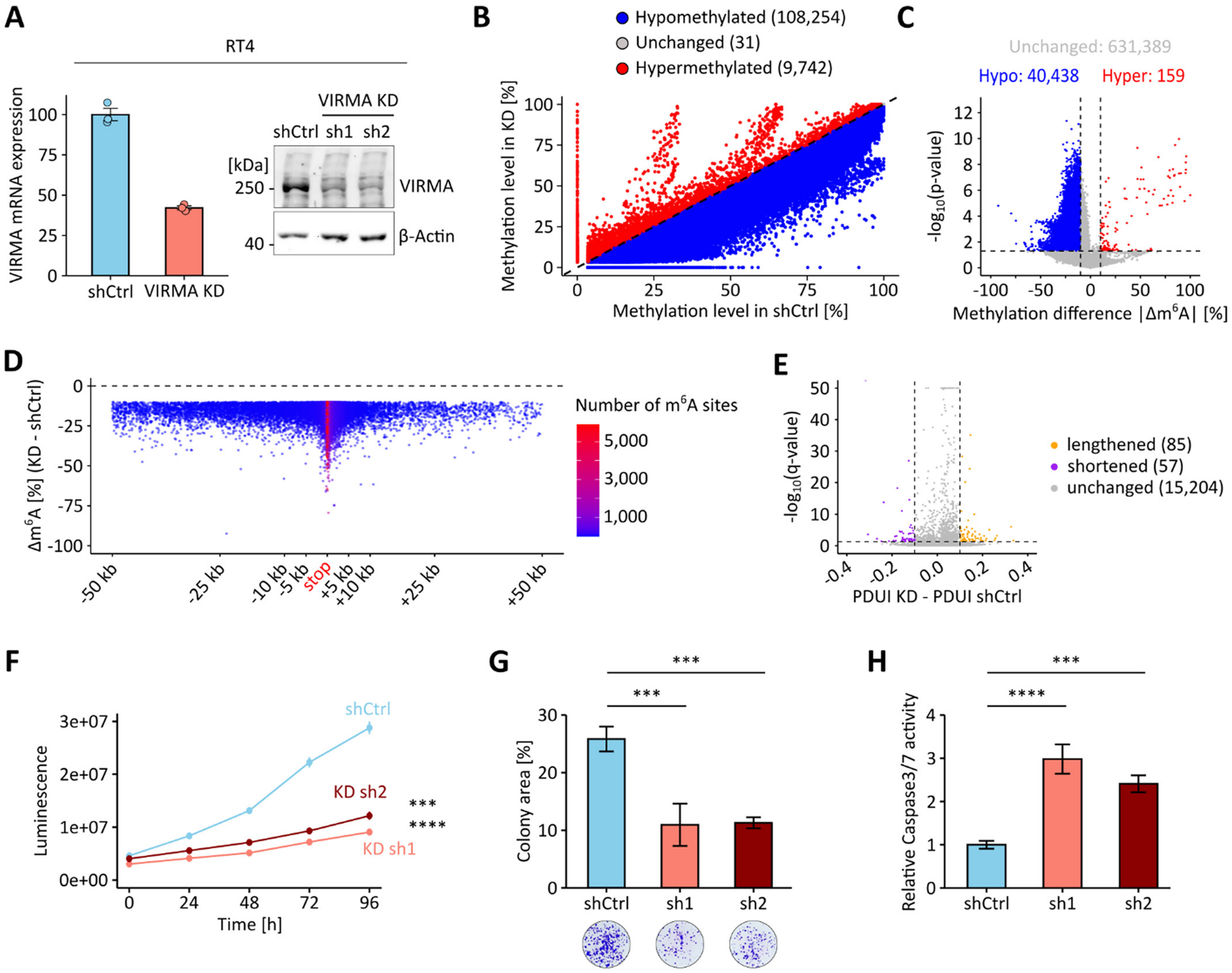

Figure EV8. VIRMA depletion reduces m6A methylation and impairs the oncogenic phenotype of RT4 cells.

(**A**) RNA-seq and Western blot analyses of VIRMA expression levels in RT4 VIRMA KD and shCtrl cells. (**B**) Scatter plot comparing mean m6A levels of methylated DRAC(H) sites between RT4 VIRMA KD and shCtrl cell lines. (**C**) Overview of differentially methylated DRAC(H) sites in VIRMA-depleted RT4 cells, based on |Δmethylation| > 10% and $p < 0.05$ thresholds. Statistical significance was assessed using beta binomial models. Unmethylated DRAC(H) sites are included in this analysis. (**D**) Δm6A levels (RT4 VIRMA KD - shCtrl) for differentially hypomethylated m6A sites were plotted across transcript regions surrounding the stop codon. (**E**) DaPars-based analysis of APA showing ΔPDUI for RT4 VIRMA-depleted cells compared to shCtrl cells. These analyses were performed based on $n = 3$ biological replicates. (**F**) Cell proliferation of RT4 VIRMA KD and shCtrl cells. sh1****$p < 0.0001$, sh2***$p = 0.0008$, two-way analysis of variance. (**G**) Colony formation results from RT4 VIRMA KD and shCtrl cells. ***$p = 0.0007$, two-tailed Student's t test. (**H**) Caspase 3/7 activity measurements in RT4 VIRMA KD and shCtrl cells. sh1****$p < 0.0001$, sh2***$p = 0.0001$, two-tailed Student's t test. Data are represented as mean ± SD; $n = 4$ biological replicates. Source data are available online for this figure.

© The Author(s)

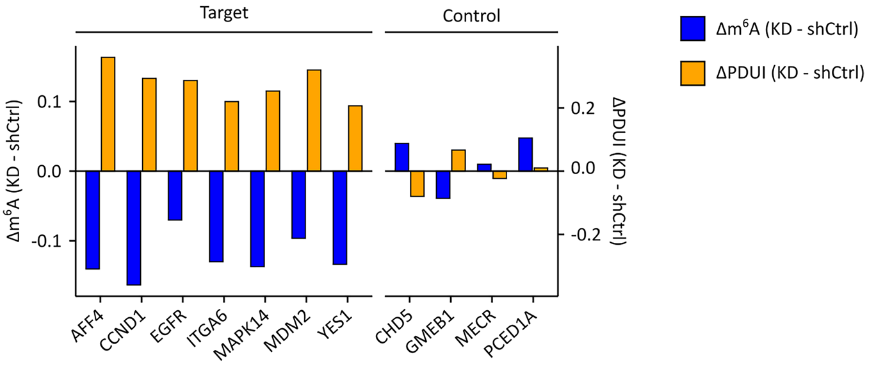

Figure EV9. VIRMA KD-associated changes in m^{6}A methylation and alternative polyadenylation at the transcript level.

Bar plots show changes in m^{6}A methylation (Δm^{6}A; left y-axis) and polyadenylation site usage (ΔPDUI; right y-axis) upon VIRMA KD in UM-UC-3 cells. Δm^{6}A was calculated as the mean m^{6}A methylation level across all identified m^{6}A sites within the terminal exon and 3′-UTR of each transcript. Target genes show reduced m^{6}A methylation accompanied by increased ΔPDUI values, indicative of transcript lengthening. Δm^{6}A and ΔPDUI values are shown relative to shCtrl conditions, $n = 3$ biological replicates.

© The Author(s)
